# Synthesis of Pellet-Based Pd/C Egg-Shell Catalysts for Reversible Hydrogen Storage in Formate/Bicarbonate

**DOI:** 10.1021/acssuschemeng.5c11492

**Published:** 2026-01-02

**Authors:** Carolin A. M. Stein, Jonas Massa, Katja Neubauer, Stephan Bartling, Carsten Kreyenschulte, Hanan Atia, Lorenz Dittrich, Hung Mac, Rui Sang, Volkan Turan, Peter Sponholz, Ali M. Abdel-Mageed, Sebastian Wohlrab, Henrik Junge, Matthias Beller

**Affiliations:** 1 28392 Leibniz-Institut für Katalyse e.V., Albert-Einstein-Straße 29a, 18059 Rostock, Germany; 2 H2APEX, Hans-Adam-Allee 1, 18299 Rostock-Laage, Germany

**Keywords:** Hydrogen storage, Formate bicarbonate interconversion, Pellet catalyst, Egg-shell type catalyst, Heterogeneous catalysis

## Abstract

We report the scalable synthesis of egg-shell Pd/C pellet catalysts for the reversible formate/bicarbonate hydrogen-storage cycle. This system is inherently safe, nontoxic and allows for the easy control of hydrogen storage and release through pressure or temperature. A simple synthesis protocol with oxalic acid as a chelating additive furnishes a uniform and thin Pd shell. The optimized catalyst is active in both half-reactions, delivering hydrogen release rates up to 17.4 L h^–1^ and 80 L of CO-free H_2_ and TON/TOF values comparable to state-of-the-art powder systems while providing the handling advantages of pellets. Insights into catalyst reactivation strategies are provided to improve the stability of the optimal material during repeated use.

## Graphical abstract

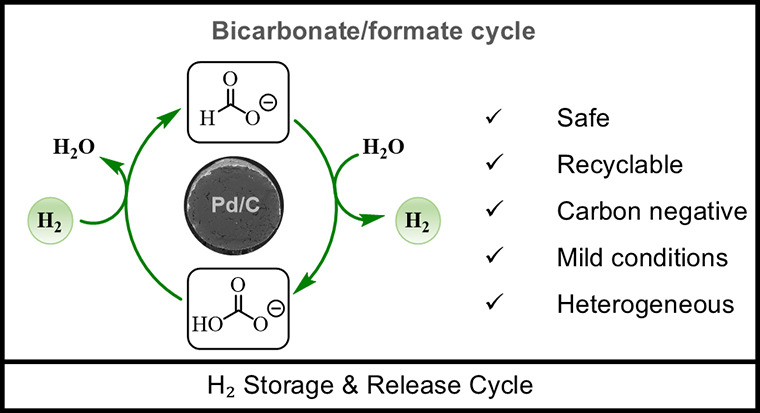

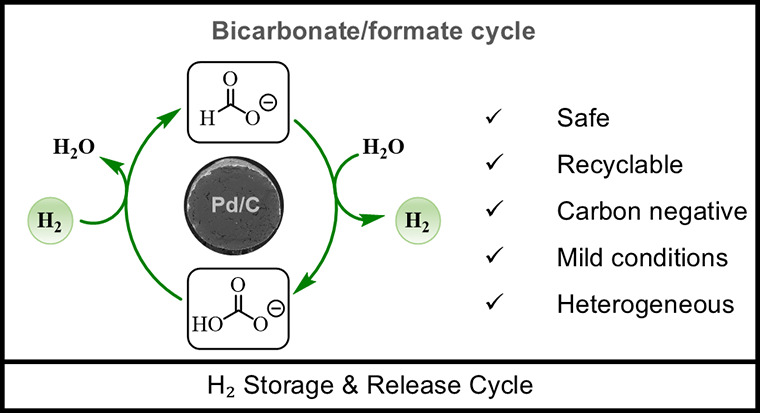

## 1 Introduction

In 2024, fossil fuels accounted for 37.4 billion tons of the global CO_2_ emissions, about 90% of the total and 0.8% higher than the previous year.^1^ Achieving carbon neutrality and mitigating climate change necessitate the decarbonization of the energy sector. Green hydrogen, produced via water electrolysis using renewable energy, offers high energy density and CO_2_ neutrality. However, their volatility and low volumetric energy density pose challenges for storage, transport, and utilization. Besides compression and liquefaction, chemical storage of hydrogen has been proposed using metal hydrides^2,3^ or liquid organic hydrogen carriers (LOHC) like arenes^4,5^ as well as bulk chemicals such as ammonia,^6,7^ formic acid,^8–10^ and methanol.^11,12^ Among these, the formate/bicarbonate redox couple is especially attractive due to its green nature: low toxicity, noncorrosiveness, and formation/consumption of only water. Notably, formate can be produced directly from CO_2_, enabling its integration into a circular carbon economy. In this cycle, bicarbonate is hydrogenated to formate (bicarbonate hydrogenation, BH), and formate is dehydrogenated to release hydrogen (formate dehydrogenation, FD) (Scheme 1).^15^ Both homogeneous and heterogeneous catalysts have been reported for the individual half-reactions (Supporting Information, Table S1).^17–20^ Typically, palladium-based on activated carbon (AC), carbon nitrides (CN), and metal oxides, as well as mixed metal catalysts, constitute the most active systems. For BH, 5 wt % Pd/AC achieved formate yields of 43% and a TON of 782.^21^ Yields up to 85% were reported for mesoporous graphitic CN (2.0 wt % Pd).^22^ For FD, Pd/AC (3 wt %) provided a TOF of 6190 h^–1^ (92% H_2_), and co-doped Pd/N,P-C showed a TOF of up to 3246 h^–1^.^23^

**Table 1:** Properties of Different Chemical Hydrogen Carriers versus Compressed Hydrogen^11,13–16^

hydrogen carrier	toxicity	storage conditions	H_2_ content [wt %]	volumetric energy density [g/L]	gravimetric energy density [MJ/L]
H_2_	-	700 bar	100	41.4	4.97
MgH_2_	toxic	ambient	7.6	110	13.2
perhydrobenzyltoluene/benzyltoluene	toxic	ambient	6.2	56.0	6.72
ammonia	toxic	–33.5 °C, 1 bar	17.6	136	11.5
methanol	toxic	ambient	12.6	150	17.8
formic acid	toxic	ambient	4.4	53.4	5.9
formate^a^/bicarbonate	-	ambient	2.2	45.7	2.0^b^

a Solid potassium formate.

b 10 M solution of potassium formate.

**Scheme 1:**
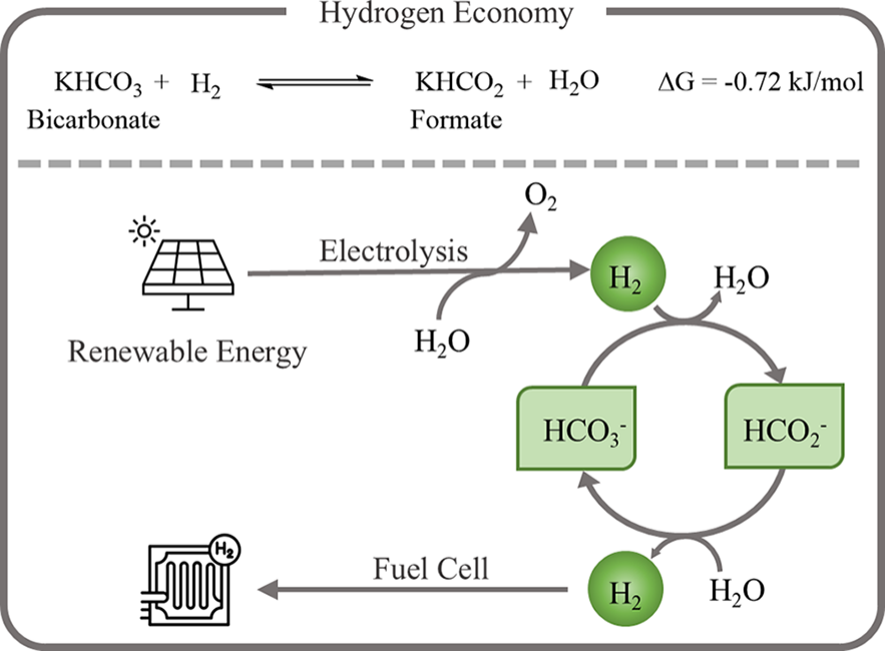
Hydrogen Storage Based on the Formate/Bicarbonate Redox Equilibrium^a^

a Excess electricity is converted into green hydrogen via electrolysis. Hydrogen can be safely stored in formate by hydrogenolysis of bicarbonate. After release, hydrogen can be converted into electricity in a fuel cell.

Recent studies explored recyclable, bifunctional catalysts that operate efficiently in *both* BH and FD. Our group demonstrated a homogeneous Ru catalyst capable of 40 hydrogenation–dehydrogenation cycles lasting over 6 months and still maintaining its activity.^24^ Among heterogeneous catalysts Pd/rGO (reduced graphite oxide), shows outstanding stability and was used for six consecutive charge/discharge cycles without loss of efficiency.^25^ Pd–Au/AC alloys show complementary adsorption properties that enable reversible hydrogen uptake and release with TOFs up to 5820 h^–1^ for BH and 4200 h^–1^ for FD.^26^ This catalyst system was recycled twice for FD. TiO_*x*_-shell Pd–Ag/TiO_2_ systems likewise exhibit high activity in both directions (BH, TON ≈ 820; FD, TOF ≈ 6500 h^–1^) while suppressing CO adsorption. Here both half-reactions were recycled twice.^27^ Despite these advances, catalyst recyclability and long-term stability, as well as the ability to perform storage cycles (BH + FD), need to be improved further for an industrial application.^28,29^

To bridge this gap, we focus on the design of Pd/C catalysts for a reversible formate/bicarbonate hydrogen storage system. Our goal is to develop a recyclable, long-term stable catalyst with high activity in both half-reactions, using a synthesis that is economically viable, scalable, and technically straightforward for large-scale production. Structured pelletized carbon supports are employed to meet industrial requirements.^31^ Carbon-based supports offer high surface area, aid in metal recovery and recycling, and align with sustainable industrial practices.^30^ Pellets provide mechanical robustness, enable efficient heat and mass transfer, and facilitate simple catalyst handling, separation, and recycling. Metal loading was performed via egg-shell impregnation. This method concentrates the active material in a thin outer layer, ensuring rapid product migration and minimizing undesired side reactions while allowing uniform macroscopic distribution of active sites. This combination of pellet geometry and egg-shell architecture offers a practical pathway toward stable, easily recoverable heterogeneous catalysts for long-term hydrogen storage.

## 2 Materials and Methods

### 2.1 Catalyst Preparation

General procedure for carbon support screening: A solution of Na_2_PdCl_4_ (0.40 mmol, 20.0 mL of H_2_O) was added dropwise to 808 mg of carbon support in 40.0 mL of H_2_O. The reaction was stirred for 12 h at room temperature, filtered and washed. The dry sample was reduced with 5% H_2_ (2 h, 200 °C, and 5 °C/min).

General procedure for the synthesis based on AKROS C1: A solution of Na_2_PdCl_4_ (0.08–0.40 mmol, 10.0 mL of H_2_O; if noted: chelating additive) was added dropwise to 808 mg of carbon pellet support AKROS C1 in 50.0 mL of H_2_O at room temperature or 60 °C. The reaction solution was stirred until it cleared up. The solvent was removed via filtration (for room temperature) or via evaporation at 60 °C. If noted, the catalyst was calcinated (2 h, 200 °C). Then the catalyst was reduced with 5% H_2_ (1–4 h, 100–400 °C, and 5 °C/min).

Synthesis of Pd-C1-OA-O_2_: A solution of Na_2_PdCl_4_ (1.00 mmol) and 2.0 equiv of oxalic acid (1.98 mmol) in 30.0 mL of H_2_O was added dropwise to 5 g of AKROS C1 in 95.0 mL of H_2_O at 60 °C. The reaction solution was stirred until it cleared up. The solvent was evaporated (60 °C). The washed and dried catalysts was calcinated (2 h, 200 °C), then reduced with 5% H_2_ (50.0 mL/min, 2 h, 200 °C, 5 °C/min).

### 2.2 Catalyst Performance Test

Bicarbonate hydrogenation (BH) and formate dehydrogenation (FD) were performed to evaluate the catalysts performance. In a typical hydrogenation (BH) 5.40 mmol of potassium bicarbonate and 20 mg of catalyst were place in 12.0 mL vial, evacuated, flushed with Ar and dissolved in 1.5 mL of degassed H_2_O. The vials were positioned in a 300 mL autoclave, flushed with H_2_ three times, and pressurized with 30 bar of H_2_. The reaction mixture was heated at 60 °C for 18 h. The formate yield was determined by ^1^H NMR based on the internal standard DMSO.

FD was performed in a double walled glass reactor connected to a manual buret at ambient pressure. The reaction vessel was loaded with 250.00 mmol of potassium formate, 7.50 mmol of potassium carbonate, and 106 mg of catalyst. The vessel was evacuated, flushed with Ar and filled with 25.0 mL of degassed H_2_O. The reaction was heated to 60 °C and dehydrogenation was performed for 3 h. Yield is based on the evolution of H_2_ (gas constitution verified via GC; CO quantification limit <10 ppm).

### 2.3 Catalyst Characterization

Elementary analysis (EA) was performed with a Leco TruSpec Micro CHNS elemental analyzer for the quantification of C, H, N, and S. Other elements were quantified via atomic emission spectroscopy (ICP-OES) using a Varian/Agilent 715-ES. Pellets were analyzed as powders.

Scanning electron microscopy (SEM) analysis was performed on a Thermo Fisher Scientific (Hillsboro, USA) Quattro S equipped with a Thermo Fisher Scientific UltraDry 60M energy dispersive X-ray spectrometer (EDXS) for elemental identification. A secondary electron (SE) detector (Everhart-Thornley type) and a backscatter electron (BSE) detector (solid-state detector, segmented) were used for image acquisition. The microscope was operated at a 15 kV acceleration voltage. Pellet samples were analyzed as presented.

Scanning transmission electron microscopy (STEM) measurements were performed at 200 kV with a probe aberration-corrected JEM-ARM200F (microscope, JEOL, Japan; corrector, CEOS, Germany) using annular bright field (ABF) and high angle annular dark field (HAADF) detectors. The microscope is equipped with a DRY SD60GV (JEOL) energy-dispersive X-ray spectrometer (EDXS) for chemical analysis. The outer pellet surface has been scraped off the pellet and was deposed on a holey carbon supported Cu-grid (mesh 300) and transferred to the microscope.

X-ray photoelectron spectroscopy (XPS) was performed on an ESCALAB 220iXL (Thermo Fisher Scientific) instrument with monochromated Al Kα radiation (*E* = 1486.6 eV). Pellet samples were prepared on a stainless-steel holder with conductive double-sided adhesive carbon tape. The electron binding energies were obtained without charge compensation leading to a main C 1s peak at around 284.5 eV. For quantitative analysis, the peaks were deconvoluted with Gaussian–Lorentzian curves using the software Unifit 2023. The peak areas were divided by the transmission function of the spectrometer and the element specific sensitivity factor of the Scofield. The depth profiling was performed in the same machine using EX-05 sputter source (Thermo Fisher Scientific) with an argon pressure of 2 × 10^7^ mbar with an acceleration voltage of 2 kV, resulting in a sputter current of approximately 2.0 μA.

In addition, H_2_ chemisorption was performed using a 3Flex apparatus. The catalyst was reduced prior to measurement, then it was subjected to CO pulses (20 CO/He) through a dosing loop until no consumption of the CO at the thermal conductivity detector (TCD) was measured. The peak areas obtained via TCD are used for the calculation of the particle size. A stoichiometry of CO:Pd = 1:1 was assumed.

Diffuse reflectance Fourier transform infrared spectroscopy (DRIFTS) measurements were carried out by using a Praying Mantis high-temperature reaction chamber (Harrick Scientific Products, Inc.). The spectra were recorded with a Nicolet iS 50 FT-IR spectrometer (ThermoFisher) equipped with an MCT detector. The catalyst was diluted with α-Al_2_O_3_ (95 wt % Al_2_O_3_ and 5 wt % catalyst) and then placed in the reaction cell. The cell was purged with Ar before measurements. The background spectra were collected on an inert support under Ar flow

Zeta potential measurements were conducted using a Zetasizer Ultra Red coupled with the MPT-3 multipurpose titrator (Malvern Panalytical Ltd.). The Zetasizer operates on the principle of electrophoretic light scattering (ELS) employing the M3-PALS technique, ensuring high precision in zeta potential measurement. The MPT-3 titrator was utilized to systematically adjust the pH of the solution during the measurement process. Pellets have been analyzed as powders.

## 3 Results and Discussion

Based on our recent results applying homogeneous Ru-based catalysts,^24^ we sought to optimize the formate/bicarbonate hydrogen storage system with a focus on industrial applicability. In our previous work a triglyme/water solvent mixture with Ru-Macho-^*i*^Pr gave formate yields up to 74% (TON = 9650) for BH.^26^ In the present study, we selected water exclusively as a solvent. Although organic cosolvents can enhance catalyst activity, they introduce toxicity, flammability, making them unattractive for large-scale applications.^30^ Despite their high activity and selectivity, homogeneous catalysts remain difficult to separate from products, limiting industrial feasibility. Therefore, the performance of commercially available heterogeneous catalysts was investigated. Among the metals tested, Pd demonstrated the highest activity in BH (up to 49% yield; Supporting Information, Table S2). To improve processability and recyclability, we evaluated pelletized catalysts, which are standard in industry but rarely explored for this hydrogen-storage system.^18^ Pellet supports facilitate efficient separation of the product and improve stability. However, commercially available pellet systems leak in activity during operation (0–2% yield in BH, Supporting Information, Table S2, entries 20–24). To overcome these limitations, we designed shell-impregnated Pd/C pellet catalysts tailored for reversible hydrogen storage and release. Key synthesis parameters, like metal loading, reduction, calcination, and impregnation, were systematically varied to maximize activity and stability. The catalyst performance was evaluated in both half-reactions, bicarbonate hydrogenation (BH) and formate dehydrogenation (FD), under the general conditions shown in Scheme 2.

**Scheme 2:**
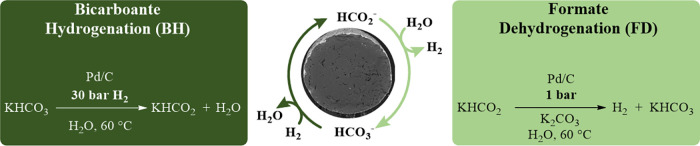
Reaction Conditions for the Testing of Different Catalysts^a^

a BH: 5.40 mmol of KHCO_3_, 20 mg of catalyst, 1.5 mL of H_2_O, 30 bar of H_2_, 60 °C,18 h. FD: 250 mmol of KHCO_2_, 7.50 mmol of K_2_CO_3_, 106 mg of catalyst, 25.0 mL of H_2_O, 60 °C, 3 h.

### 3.1 Investigation of Different Carbon Supports

As a starting point, we modified the approach outlined by the Jiang group.^31^ In the original paper, palladium is deposited from a Na_2_PdCl_4_ precursor and reduced on carbon powder in the presence of citric acid. We adapted this approach and performed the deposition of Pd on several carbon supports in a few optimization steps. To this end, water was added to various commercially available pellets. Under stirring, a solution of the palladium precursor was added dropwise to the support at room temperature and stirred for 12 h. The pellets were filtered, washed, dried, and reduced with H_2_ at 200 °C. The palladium loadings and performances in (de)­hydrogenation reactions are shown in Table 2. The goodness of the catalysts was evaluated by comparing their mass activities calculated based on the actual metal loadings. Due to deviations in the theoretical and measured metal loading, we chose the mass activity based on the maximum available amount of active material (measured metal content) to evaluate the catalytic performance. The results are shown in plot A.

**Table 2:**
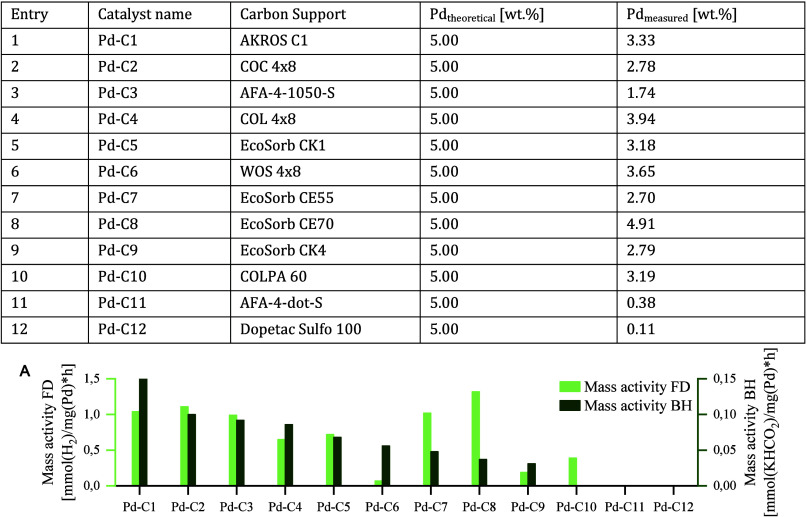
Pd/C Catalysts Synthesized Using Different Carbon Pellet Supports and Their Mass Activity^a^

a Plot A: mass activities based on the measured metal loading. Catalyst synthesis: Na_2_PdCl_4_ (400 μmol, corresponding to 5.00 wt %, 20.0 mL of H_2_O), carbon support (808 mg, 40.0 mL of H_2_O), rt, 12 h, filtered, washed and dried. The samples were reduced with H_2_ (2 h at 200 °C). Catalytic screening, BH: 5.40 mmol KHCO_3_, 20 mg of Pd/C, 1.5 mL of H_2_O, 30 bar of H_2_, 60 °C, 18 h. FD: 250.00 mmol of KHCO_2_, 7.50 mmol of potassium carbonate, 106 mg of catalyst, 25.0 mL of H_2_O, 60 °C, 3 h.

The objective of the catalyst synthesis was to achieve a Pd loading of 5.00 wt %. However, significant variations in the actual metal loading were observed for different carbon supports. Catalysts with Pd contents below 1.00 wt % showed no detectable activity, while those above this threshold exhibited no direct correlation between Pd loading and catalytic performance.

To understand this variation, analytical studies were conducted on specific supports and their corresponding catalysts. The measured metal loadings indicate a higher Pd loading capacity for some of the carbon supports, suggesting different functional groups on the carbon surface with varying adsorption affinities for Pd. The elementary composition of the carbon supports with elementary analysis (EA) and X-ray fluorescence spectroscopy (CHN analysis and XRF; Supporting Information, Tables S20–S22) showed a carbon content ranging from 92 to 72%. Sulfur and chlorine, both predominantly found in mediocre and poorly performing supports (1–2%), often act as catalyst poisons by binding to active metal sites.^32,33^ In addition, promoters such as potassium and calcium^34^ were mainly present in high quantities in poorly performing supports, suggesting that excessive amounts of these elements reverse their positive effect. Zeta potential measurements revealed isoelectric points (IEP; pH of surface electrostatic neutrality) between 1.62 and 3.12 (Supporting Information, Table S28). These values indicate a negatively charged surface above pH ≈ 3, caused by deprotonated oxygen-containing functional groups such as carboxyl.^35^ Since both the BH (pH ≈ 8) and FD (pH ≈ 9) reactions are carried out well above the IEP, the catalyst surface is expected to be negatively charged under reaction conditions, which may influence substrate adsorption and ion transport during the interconversion. Scanning electron microscopy (SEM; Supporting Information, Figures S16–S24) investigations highlighted that catalytic activity correlates with the uniformity of the Pd crust rather than with the total loading. Figure 1 shows backscattered electron images of the surface (top) and cross-section (bottom) of a well-performing (Pd-C2, Figure 1, left, 2.78 wt % Pd) and a mediocre-performing catalyst (Pd-C5, Figure 1, right, 3.18 wt % Pd). Highly active catalysts like Pd-C2 exhibit a relatively uniform metal distribution on the surface (Figure 1, left, and top). The presence of black spots was attributed to crust chipping. Little to no palladium was found on the inside, indicating an eggshell-type catalyst (Figure 1, left, bottom). The cross-section revealed an even thickness of the palladium crust on the outer surface of the support. Conversely, poorly working catalysts like Pd-C5 show an unevenly impregnated surface, although the metal loading is higher (Figure 1, right, top). This disparity is more pronounced upon examination of the cross-section (Figure 1, right, bottom), which reveals a variable thickness of the metal from no palladium to thicker layers.

**Figure 1:**
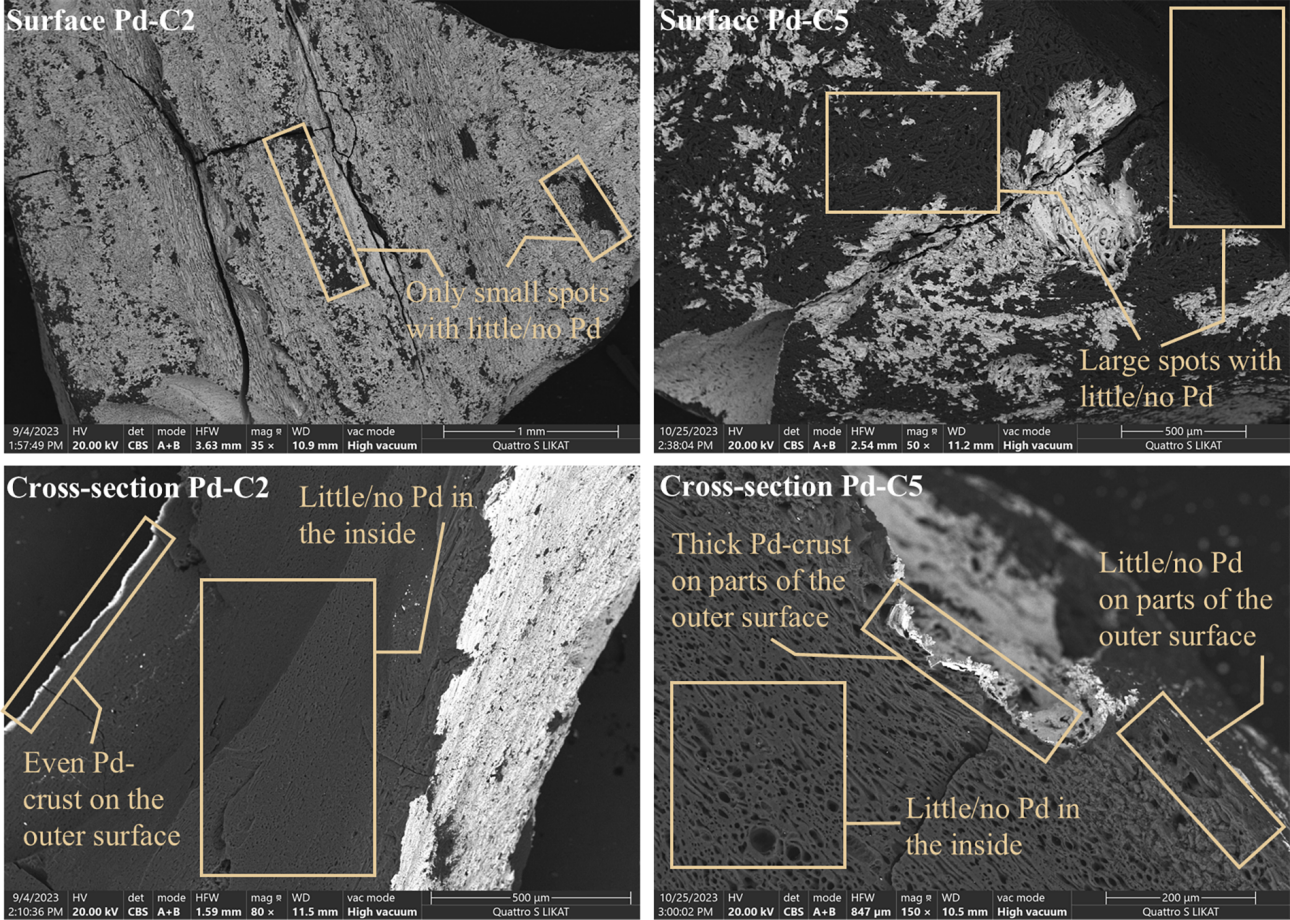
Backscattered electron (BSE) images of well-performing (Pd-C2; left) and mediocre-performing catalysts (Pd-C5; right). Top: surface. Bottom: cross-section. Lighter regions on the outer surface correspond to Pd, while dark regions represent carbon. Further enlargements can be seen in the Supporting Information (Figures S17 and S20).

Next, we analyzed the chemical state of the freshly prepared catalysts via X-ray photoelectron spectroscopy (XPS; Supporting Information, Figures S33–S41). The obtained results indicate the presence of the Pd(0) species at 335.2 eV as well as Pd­(II) around 336.9 eV.^36^ Among the different supports, the ratio of Pd(0) to Pd­(II) varies. Nitrogen sorption using the Brunauer–Emmett–Teller method was used to investigate the external surface area of the catalysts (Supporting Information, Table S25). All of the analyzed catalysts exhibit large external surface areas. These results demonstrate that catalyst activity is not simply dictated by the total Pd loading but by support surface chemistry and the nature and distribution of functional groups. Consistent with recent studies, the performance is largely determined by Pd dispersion, electronic state, and accessibility, which are strongly shaped by metal–support interactions.^37,38^

### 3.2 Further Optimization of the Catalyst Synthesis Based on AKROS C1 Carbon Support

The AKROS C1 support was chosen for further optimization due to its high Pd affinity and effective catalytic activity. A summary of the optimizations is given in Table 3, while the mass activity based on the actual metal loading can be observed in plots B–E.

**Table 3:**
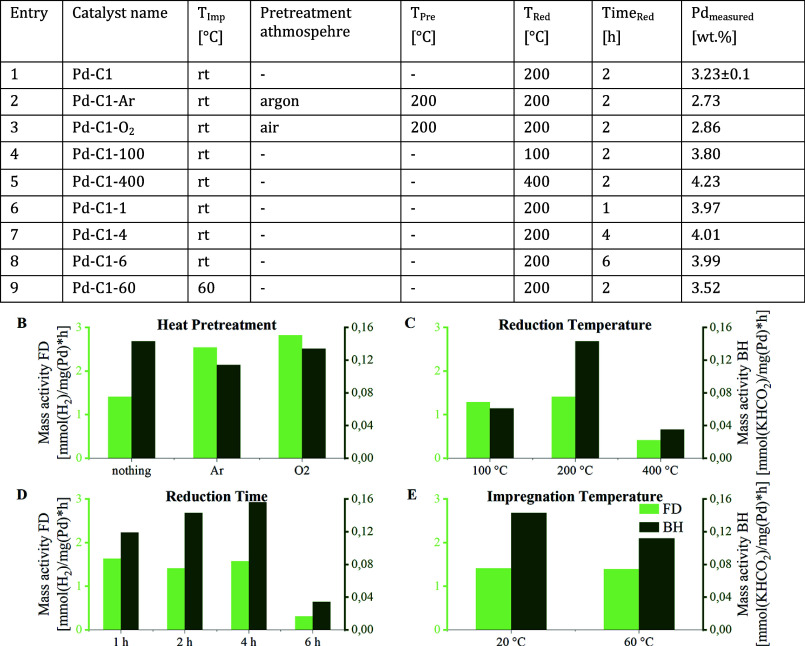
Optimization Steps in the Synthesis of Shell-Impregnated Pd/C Catalysts Based on AKROS C1 and Their Mass Activity in the (De)­Hydrogenation^a^

a The mass activity for BH and FD are presented in plots B–E. Plot B: entries 1–3. Plot C: entries 1, 3, 4. Plot D: entries 1, 6–8. Plot E: entries 1 and 9. Catalyst synthesis: Na_2_PdCl_4_ (400 μmol, 10.0 mL H_2_O) was added dropwise to AKROS C1 (808 mg, 50.0 mL H_2_O) at rt or 60 °C. For reactions at rt, the solvent was removed by filtration. For reactions at 60 °C, the solvent was evaporated. The catalyst was washed, dried, and reduced with H_2_ at the mentioned temperature. Catalytic screening, BH: 5.40 mmol of KHCO_3_, 20 mg of Pd/C, 1.5 mL of H_2_O, 30 bar of H_2_, 60 °C, 18 h. FD: 250 mmol of KHCO_2_, 7.50 mmol of K_2_CO_3_, 106 mg of catalyst, 25.0 mL of H_2_O, 60 °C, 3 h.

First, the reproducibility of Pd-C1 was confirmed (Table 3, entry 1). Screening of different Pd precursors revealed Na_2_PdCl_4_ as the most effective, giving the highest metal loading (Supporting Information, Table S3, entries 5–7), and it was therefore used for all subsequent experiments. Heat treatment procedures play an important role in controlling the catalyst’s morphology, activity, and stability.^39^ As shown in plot B, the heat pretreatment of the impregnated carbon support under argon Pd-C1-Ar or air Pd-C1-O_2_ (Table 3, entries 2 and 3) did not significantly alter the activity. Nevertheless, ICP analysis before and after BH (Supporting Information, Table S6) indicated that calcination improved the stability of the metal on the surface.

Variation of the reduction temperature (Table 3, entries 4 and 5; plot C) clearly showed an optimum temperature for 200 °C (Pd-C1, Table 2, entry 1). An increase of the reduction temperature can lead to a decrease in dispersion of the metal.^40^

A reduction time between 1 and 4 h (Table 2, entries 6 and 7) did not demonstrate a distinct optimum as evident from Plot D. Shorter reduction led to a decline in activity regarding BH, while similar outcomes were observed for FD. A longer reduction exhibited a modest enhancement for BH and FD.

Nevertheless, the reduction for 6 h lowered the activity drastically (Table 3, entry 8), indicating the necessity of a certain amount of Pd­(II). Wet impregnation at elevated temperatures was tested to streamline the synthesis method and mitigate Pd loss (Table 3, entry 9; plot E). While the mass activity based on the metal amount remained constant for Pd-C1-60, this impregnation method enhanced the overall metal loading. Using wet impregnation at elevated temperatures, the addition of chelating agents during impregnation was investigated (Table 4, entries 1–6). Inspired by the positive effect of citric acid as published by Jiangs group,^31^ several multidentate ligands were tested to prevent precursor crystallization by increasing solution viscosity and thus improving Pd dispersion on the surface.^41^ The equivalents of additives were chosen based on their denticity to ensure optimal complexation of the palladium precursor. The mass activity of the resulting catalyst materials in the (de)­hydrogenation reaction can be seen in plot F (Table 4). Among the tested ligands, oxalic acid (entry 6) proved to be effective. It minimized the Pd loss during synthesis, thereby increasing the metal loading of the catalyst. The synthesis yielded smaller, more dispersed particles, which enabled a uniform coating of the pellet surface, demonstrated by SEM (Supporting Information Figure S25). This observation becomes evident when the metal dispersion is compared via CO chemisorption analysis of Pd-C1 and 3.90Pd-C1-OA (Supporting Information Table S27). While the active particle diameter is drastically reduced by the addition of oxalic acid (20.2 nm vs 8.5 nm), the metal dispersion is increased (5.6% vs 13.1%).

**Table 4:**
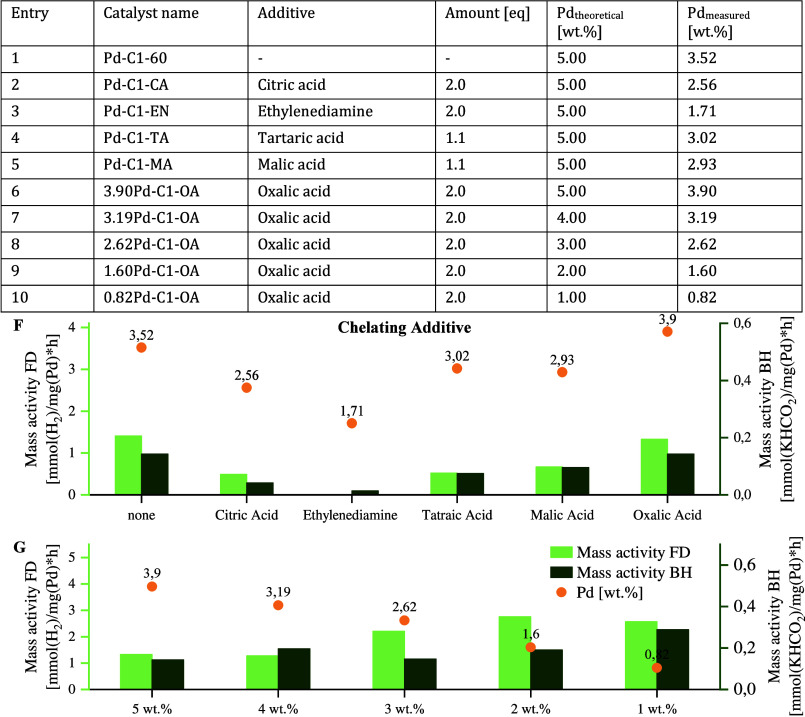
Influence of Different Additives in the Impregnation Step of the Catalyst Synthesis on the Metal Loading and Catalytic Activity in the (De)­Hydrogenation Reactions^a^

a The mass activities for BH and FD are presented in plots F and G. Plot F: entries 1–6. Plot G: entries 6–10. Catalyst synthesis: An aqueous mixture of Na_2_PdCl_4_ and the additive (10.0 mL of H_2_O) was added dropwise to the carbon pellet AKROS C1 (808 mg, 50.0 mL of H_2_O) at 60 °C. The solution was stirred until it cleared up. The solvent was evaporated. The catalyst was washed, dried, and reduced with H_2_ (2 h, 200 °C). Catalytic screening, BH: 5.40 mmol of KHCO_3_, 20 mg of Pd/C, 1.5 mL of H_2_O, 30 bar of H_2_, 60 °C, 18 h. FD: 250 mmol of KHCO_2_, 7.50 mmol of K_2_CO_3_, 106 mg of catalyst, 25.0 mL of H_2_O, 60 °C, 3 h.

Utilizing this knowledge, we varied the theoretical palladium loading while maintaining a high metal dispersion. Therefore, wet impregnation was done at elevated temperatures and 2.0 eq oxalic acid were used. (Table 4, entries 6–10; plot G). In contrast, syntheses without additives required 5.00 wt % Pd to achieve comparable coverage, as lower contents led to strongly inhomogeneous coatings. The addition of oxalic acid lowered the shell thickness by a factor of 10 (Supporting Information, Table S24) while keeping a homogeneous coating of the surface (Supporting Information, Figures S25–S29). The increase in the mass activity from 5.00 to 4.00 wt % indicates that a substantial amount of metal is distributed ineffectively at higher loadings, hindering its participation in the reaction process. Optimal performance was obtained with 2.0 equiv of oxalic acid.

Combining all optimization steps, we scaled up the synthesis with the goal of maximizing the activity-to-price ratio, given the inherent limitations of reactors in terms of size.^42^ Therefore, AKROS C1 pellets were impregnated with a solution of 2.00 wt % Na_2_PdCl_4_ and 2.0 equiv oxalic acid at 60 °C. The solvent was then evaporated, and the resultant dry pellets were calcinated (200 °C, 2 h) and reduced (5% H_2_, 200 °C, 2 h), yielding Pd-C1-OA-O_2_.

### 3.3 Characterization of the Catalysts Based on AKROS C1

Comprehensive structural analyses were performed using a combination of SEM, scanning transmission electron microscopy (STEM), CO chemisorption, and XPS (Supporting Information, sections 4.4, 4.6, 4.7, and 4.8). Figure 2 compares the initial catalyst Pd-C1, the oxalic-acid-modified variant 1.60Pd-C1-OA, and the final optimized material Pd-C1-OA-O_2_. Pd-C1 was prepared by wet impregnation at rt (Pd_theo_, 5.00 wt %; Table 1, entry 1), 1.60Pd-C1-OA by impregnation at 60 °C with Na_2_PdCl_4_ and 2.0 equiv of oxalic acid (Pd_theo_, 2.00 wt %; Table 4, entry 9), and Pd-C1-OA-O_2_ analogously but with an additional calcination step before reduction.

**Figure 2:**
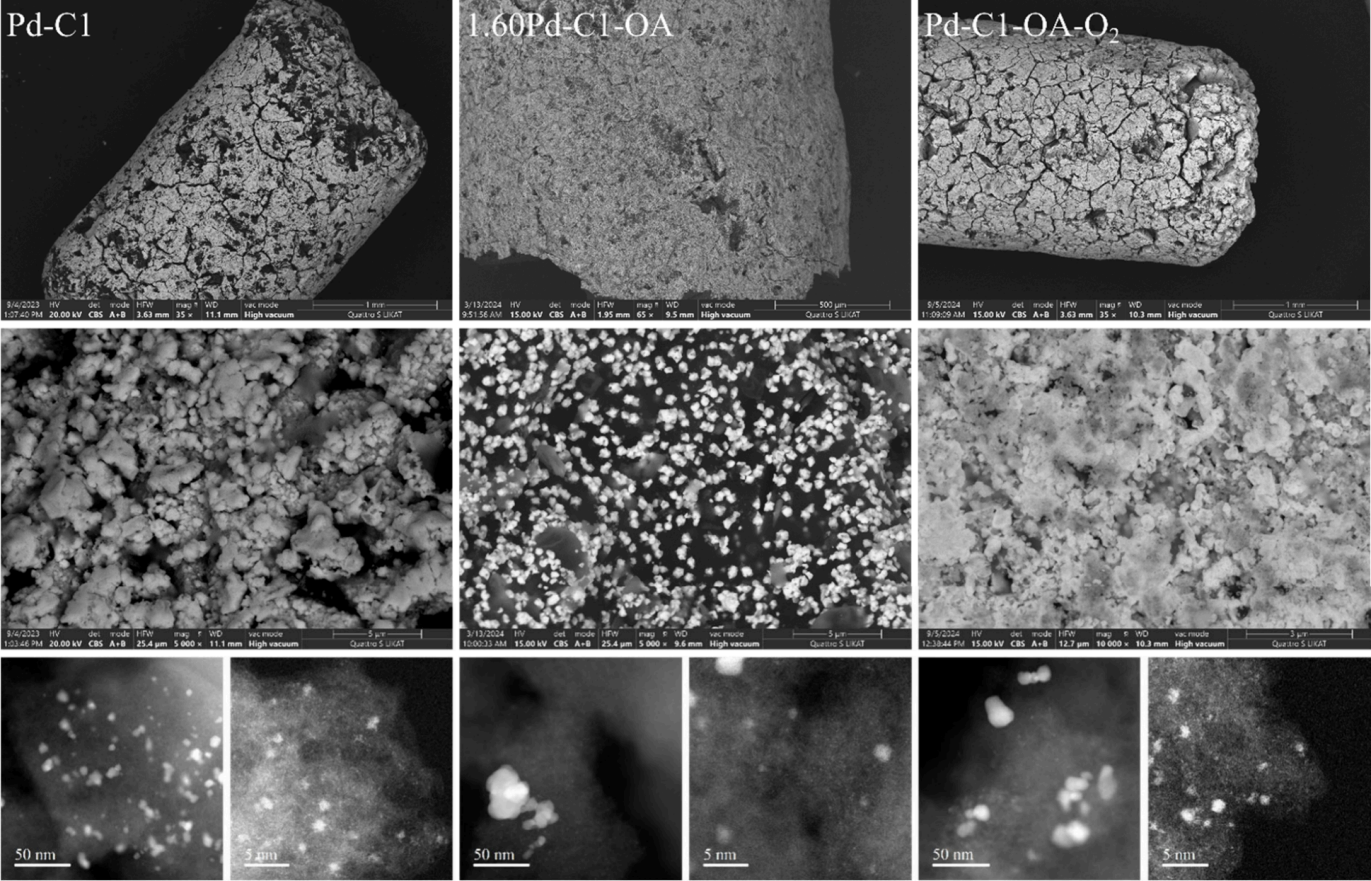
Selected BSE-SEM (whole particle) and HAADF-STEM (powder; active material scraped off from the pellet surface) images of the initial (left column, Pd-C1, 3.33 wt % Pd), an optimized (center column, 1.60Pd-C1-OA, 1.60 wt %), and the final catalyst (right column, Pd-C1-OA-O_2_, 1.81 wt %).

In addition to the larger Pd particle fraction investigated by SEM and CO chemisorption (active particle diameter: 20.1 nm), STEM provided evidence of the wide range of Pd morphology sizes present in the catalyst, scaling down to single atoms, clusters, and small particles not forming crystallites. XPS indicated a Pd(0)/Pd­(II) ratio of ∼10:1, with depth profiling confirming a Pd-rich outer crust and a higher fraction of Pd­(II) inside (Figure 3, left, bottom). It is plausible that the palladium on the surface is bound via oxygen bridges, resulting in an oxidation state of two, while the palladium on the outer crust is connected mainly via metal–metal bonds, explaining the presence of Pd(0).

**Figure 3:**
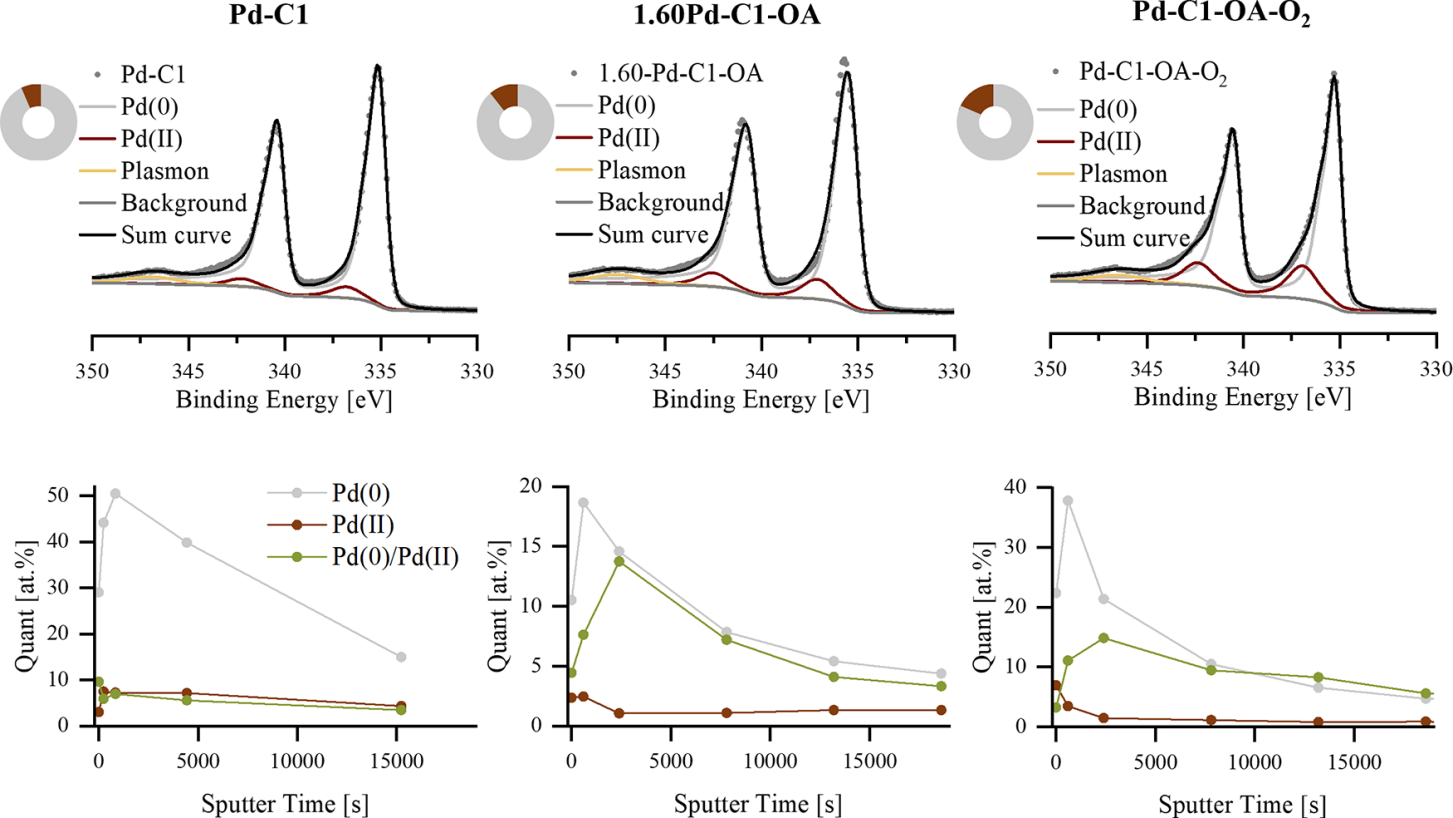
XPS (whole particle) analysis (top) as well as depth profiling experiments using a sputter source (bottom) of the initial (left column, Pd-C1, 3.33 wt % Pd), an optimized (center column, 1.60Pd-C1-OA, 1.60 wt %), and the final catalyst (right column, Pd-C1-OA-O_2_, 1.81 wt %).

Thanks to the addition of 2.0 equiv of oxalic acid in the impregnation step, we were able to reduce the palladium loading while achieving higher metal distribution on the surface (Figure 2, 1.60Pd-C1-OA, center; metal dispersion of 12.9%). SEM/energy dispersive X-ray spectroscopy (EDS) analysis confirmed uniform coverage with particles ranging from 40–550 nm, while STEM showed Pd down to single atoms. CO chemisorption revealed a drastic reduction of the active particle diameter (8.7 nm). Interestingly, XPS (Figure 3, center) showed a lower Pd(0)/Pd­(II) ratio. The data indicate that the ratio is essential, and a specific amount of Pd­(II) is beneficial for the catalytic activity, as published by the Bulusheva group for the dehydrogenation of formic acid.^43^ Depth profiling revealed an initial increase of the ratio Pd(0)/Pd­(II) indicating less metallic palladium on the surface and enrichment of Pd­(II) within the carbon.

SEM of the final catalyst Pd-C1-OA-O_2_ shows a fine, homogeneous distribution of the active material (metal distribution: 20.0%). with minimal black spots, reflecting improved crust stability after calcination. STEM confirmed sizes from single atoms up to ∼200 nm. CO chemisorption gave an active particle diameter of 5.6 nm with no signs of sintering. XPS and depth profiling indicated further increase of Pd­(II), while the microscopic structure remained unchanged (Figure 3, right).

Overall, oxalic acid addition enhanced Pd dispersion, reduced the particle size, enabled uniform coverage at lower Pd loadings, and shifted the Pd(0)/Pd­(II) ratio. The cooperative presence of both species likely contributes to activity, in line with Masuda et al., who proposed electron-deficient and electron-rich sites acting synergistically in catalytic dehydrogenation.^27^ An additional calcination step further increased the metal dispersion and stabilized the Pd crust.

### 3.4 Upscaling of the Half-Cycles

With an optimized catalyst for practical applications, activity and stability for the energy storage system (see Scheme 3). Utilizing Pd-C1-OA-O_2_, 250.00 mmol of the corresponding (loaded) hydrogen carrier, and 25.0 mL of water, we performed both BH and FD at 60 °C for 18 h. BH was monitored by the pressure drop and yielded 23% formate, corresponding to a TON of 2550. FD and gave rise to 1.14 L of gas (92% H_2_, 8% CO_2_, no CO detected) corresponding to a TOF of 3261 h^–1^. CO_2_ derived from partial decomposition of bicarbonate to carbonate and is expected at high formate concentrations.^25^ These half-cycles demonstrate the efficacy of the catalysts in facilitating both the loading and unloading of the carrier molecule, contingent on the availability of excess electricity from renewable energies (and green hydrogen). The activities exhibited by our pellet system are analogous to those previously reported for powder catalysts, including Pd/AC (BH, TON = 1672, 55 bar, 2 h, 20 °C; FD, 5061 h^–1^, 80 °C)^22^ and Pd/NMC-8 (BH, TON = 1598, 60 bar, 2 h, 80 °C; FD: 2416 h^–1^, 80 °C).^44^ The conversion of (de)­hydrogenation can be easily increased by elongation of the reaction time, as we have shown in our recent paper.^24^

**Scheme 3:**
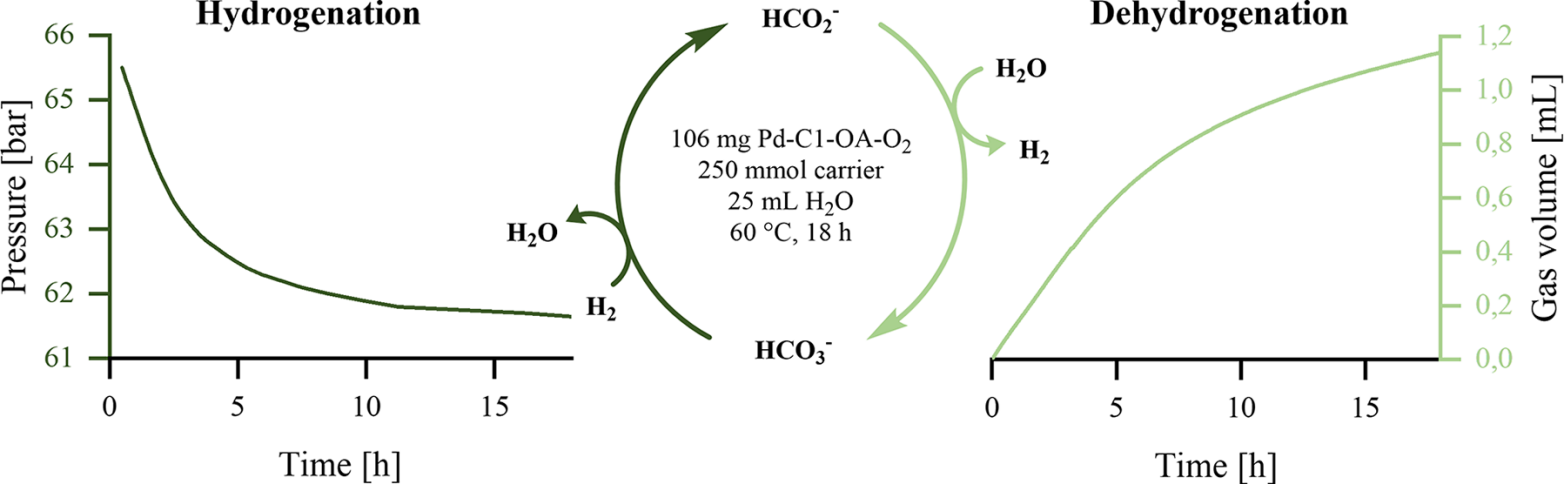
Performance of the Final Catalyst in Both Half-Cycles^a^

a BH: 250.00 mmol of KHCO_3_, 106 mg of Pd-C1-OA-O_2_, 25.0 mL of degassed water, 60 °C, 18 h. Yield was determined with ^1^H NMR using DMSO as internal standard. FD: 250.00 mmol of KHCO_2_, 7.50 mmol of K_2_CO_3_, 106 mg of Pd/C Pd-C1-OA-O_2_, 25.0 mL of degassed water, 60 °C, 18 h. Gas volume was measured with an automatic buret and gas composition verified via GC.

The long-term stability of the catalyst is a critical factor for industrial applicability. To demonstrate the recyclability, Pd-C1-OA-O_2_ was tested in repeated batch reactions. Although the pellet shape is ideal for large-scale applications, on a batch scale, the stirring bar continuously grinds the catalyst. To ensure constant reaction conditions, we ground the catalyst in the following batch reactions.

Recycling was confirmed for BH (Table 5). Here, 200.00 mmol of bicarbonate was hydrogenated with 60 bar of H_2_ for 18 h, applying 200 mg of ground Pd-C1-OA-O_2_. The first run resulted in a TON of 1570. Afterward, the catalyst was filtered, washed, calcinated (2 h, 200 °C), and reduced again (2 h, 200 °C). The second BH step achieved a similar TON of 1650. Without reactivation after the hydrogenation, only little activity was observed.

**Table 5:** Recycling of the Catalyst in BH

run no.	TON_BH_	mass activity BH [mmol(KHCO_2_)/mg(Pd)·h]
1	1570	0.82
2	1650	0.86

Figure 4 shows the results for the recycling of the catalysts in the dehydrogenation. Applying 200 mg of ground Pd-C1-OA-O_2_, the dehydrogenation was performed for 73 h. Then, the catalyst was filtered, washed, dried, and reused for a second dehydrogenation step. A total of 2.93 L of gas was released (first run, 1.36 L, 90% H_2_, 10% CO_2_, 11 ppm of CO; second run, 1.57 L, 93% H_2_, 7% CO_2_, 18 ppm of CO). Interestingly, the catalyst showed a higher activity in the second run demonstrating the positive effect of process conditions for the catalyst activity. Optimal Pd(0)/Pd­(II) ratio might be reached after the first run. A small amount of formed CO can be explained by the decomposition of formate via dehydration.^21^ This pathway should be avoided in an industrial plant because CO is toxic to fuel cells.^45^

**Figure 4:**
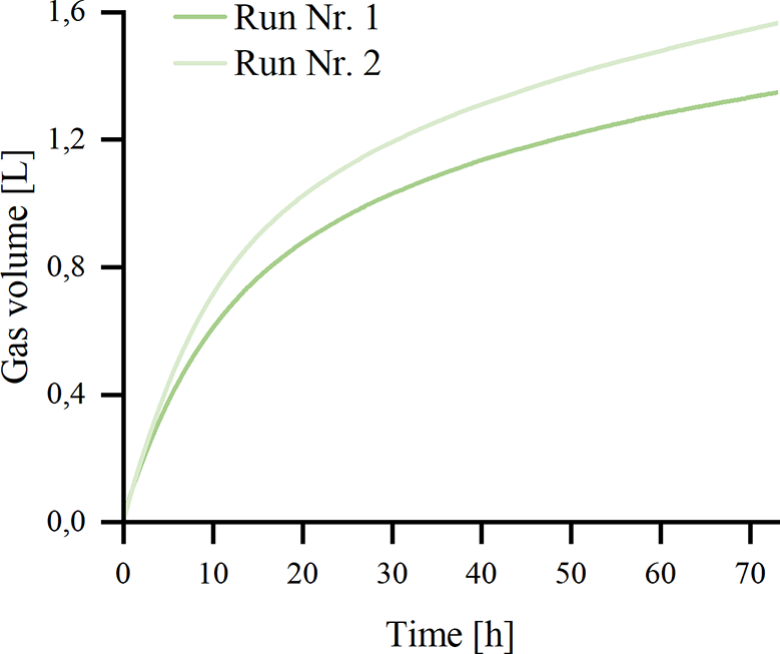
Recycling for FD: 200.00 mmol of KHCO_2_, 15.00 mmol of K_2_CO_3_, 50.0 mL of H_2_O, 60 °C, 73 h. The catalyst was filtered, washed, dried, and reused for run no. 2. Gas volume was measured with an automatic buret and gas composition verified via GC.

Finally, the catalyst was evaluated on a larger scale in a fixed-bed reactor using 10 g of material for FD as depicted in Figure 5. Overall, the catalyst was recycled 10 times and reactivated by washing and drying under air.^46^ With a decrease in activity, additional reactivation steps were assessed. Over 10 h, 71 L of gas (83% H_2_, 17% CO_2;_ Supporting Information Table S18) were released in the first run without detectable CO. Again, a higher activity was observed in the second run (82 L gas, 81% H_2_), whereas a gradual deactivation became evident from the third run onward (53 L, 84% H_2_). Reactivation via calcination, followed by reduction, proved ineffective. Increasing the reaction temperature from 60 to 80 °C neither improved activity nor facilitated product desorption, but it allowed for a more precise comparison of reactivation treatments and was therefore kept from the seventh run. Oxidative washing with diluted H_2_O_2_ resulted in a slight reactivation, and treatment with diluted HNO_3_ further enhanced the performance. ICP and SEM revealed an accumulation of potassium species on the catalyst surface (fresh catalyst, no K detectable; after first run, 1.81 wt % K), suggesting a reversible blockage of active sites by KHCO_3_. Extensive washing or higher temperatures did not completely remove these salts, and drying under a vacuum even promoted crystallization. The comparison of the diffuse reflectance infrared Fourier transform spectroscopy (DRIFT) for the fresh and used catalyst (after run no. 11) let us exclude the buildup of any surface formate or carbonate species as well as surface poisoning of Pd by CO. XPS indicated an increase of the Pd(0) proportion after repeated use (Supporting Information, Table S26). SEM and STEM analyses after one FD run showed no significant changes in particle morphology (Supporting Information, Figure S32), while CO-chemisorption detected an increase in the particle size after the 11th run (fresh, 5.6 nm vs used, 9.0 nm; Supporting Information Table S27). We conclude that deactivation of the catalyst is brought by over-reduction of palladium leading to an agglomeration of the particles.^47^ The changes in Pd oxidation state could be mitigated by oxidative treatments with air, diluted H_2_O_2_, or HNO_3_. Nevertheless, the change in particle size has yet to be addressed.

**Figure 5:**
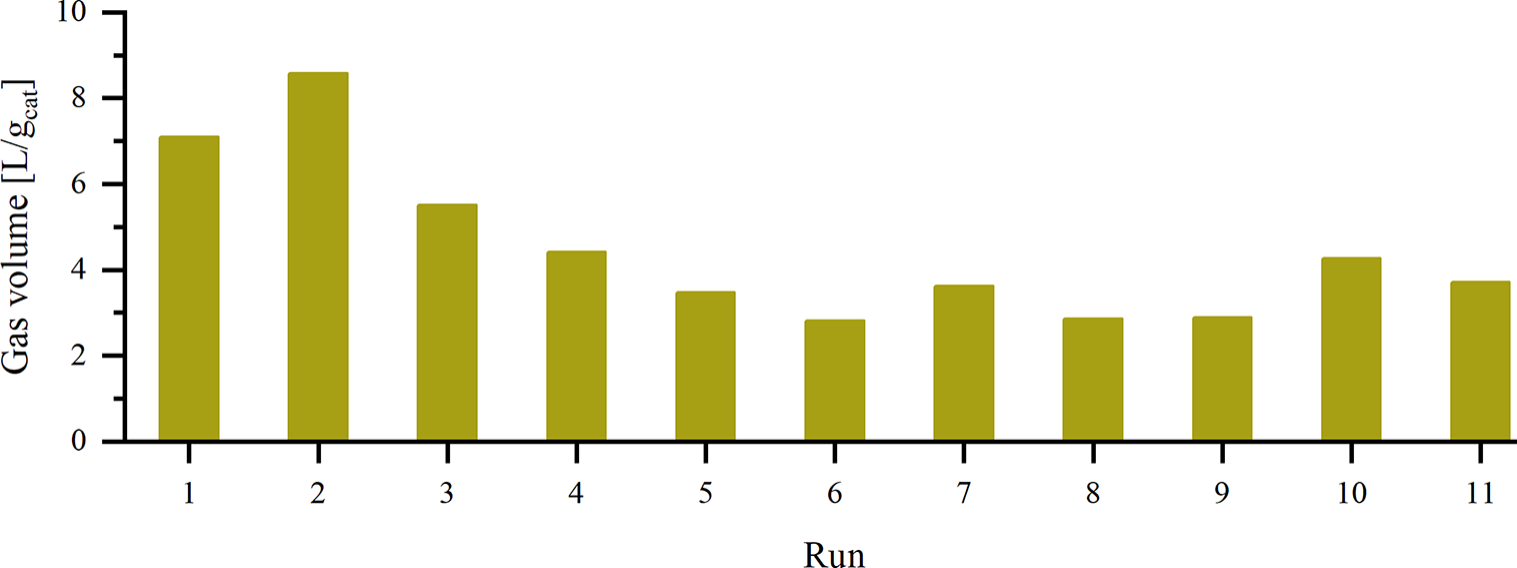
Gas evolution normalized by the amount of catalyst used for the dehydrogenation reaction. Reaction conditions: 10 g of Pd/C, 4 M KHCO_2_ in H_2_O, 0.03 L/min volume flow, 60 °C (runs 1–6) or 80 °C (runs 7–11), 10 h. Gas composition was verified via GC. The catalyst was washed and dried in air after every run. Additional reactivation methods: 6th run, calcination (2 h, 200 °C), then reduction (2 h, 200 °C); 9th run, diluted H_2_O_2_; 10th run, diluted HNO_3_.

## 4 Conclusion

We developed egg-shell Pd/C pellets that meet industrial requirements while maintaining high activity in the formate/bicarbonate hydrogen storage cycle. Oxalic acid is key to performance control: it promotes uniform, thin, highly dispersed metal shells, reduces particle size, and enables comparable activity at only ∼1.8 wt % Pd. This study demonstrates that metal dispersion and the ratio of Pd(0)/Pd­(II) are decisive physicochemical parameters for balancing activity and stability. The optimized catalyst (Pd-C1-OA-O_2_) released 416 L of gas over 11 cycles, reducing the power-specific price from ≈ 4175 €/kW_H2,th_ for the first generation to ≈ 795 €/kW_H2,th_. Deactivation is addressed to Pd over-reduction and particle growth, which can be partially reversed through mild oxidative treatments (air, dilute H_2_O_2_/HNO_3_). Beyond its catalytic performance, this strategy embodies principles of green and sustainable chemistry: it uses water as the solvent, minimizes precious-metal content, and provides a scalable, recyclable, and low-waste process. With oxalic acid as the enabling design element and controlled redox stabilization, this work lays the groundwork for sustainable, reversible hydrogen-storage technologies and the transition of the formate/bicarbonate system from laboratory research to practical energy devices.

## Supplementary Material



## Notes

Supporting Information: Detailed synthesis and experimental procedures, supplementary figures, catalyst characterizations including detailed SEM, XPS, (S)­TEM analysis, NMR spectra, and GC-MS spectra (PDF)
