# Supplementary material for: Synthesis of Pellet-Based Pd/C Egg-Shell Catalysts for Reversible Hydrogen Storage in Formate/Bicarbonate
Source: ACS Sustain Chem Eng. 2026 Jan 2;14(2):1195–206. doi: 10.1021/acssuschemeng.5c11492 (PMC12820954; doi:10.1021/acssuschemeng.5c11492)
Supplement: Supplementary file 1 [file sc5c11492_si_001.pdf]

Supporting Information

Synthesis of Pellet-based Pd/C Egg-Shell Catalysts for Reversible Hydrogen Storage in Formate/Bicarbonate

Carolin A. M. Stein,[1] Jonas Massa,[2] Katja Neubauer,[1] Stephan Bartling,[1] Carsten Kreyenschulte,[1] Hanan Atia,[1] Lorenz Dittrich,[1] Hung Mac,[1] Rui Sang,[1] Volkan Turan,[2] Peter Sponholz,[2] Ali M. Abdel-Mageed,[1] Sebastian Wohlrab,[1] Henrik Junge[1*] and Matthias Beller[1*]

[1]Leibniz-Institut für Katalyse e.V., Albert-Einstein-Str. 29a, 18059 Rostock, Germany.

[2]H2APEX, Hans-Adam-Allee 1, 18299 Rostock-Laage, Germany.

Email: matthias.beller@catalysis.de, henrik.junge@catalysis.de

Supporting information content:

Number of pages: 76

Number of figures: 60

Number of tables: 28

Table of Content

1. GENERAL INFORMATION 2

2. SELECTED STATE-OF-THE-ART CATALYSTS ACTIVE IN (REVERSIBLE) HYDROGEN STORAGE BASED ON BICARBONATE 7

3. SYNTHESIS OF CATALYSTS AND CATALYTIC SCREENING 9

3.1 REACTION SETUPS AND CALCULATIONS 10
3.2 COMPARISON OF COMMERCIAL CATALYST SYSTEMS FOR BH 16
3.3 INITIAL OPTIMIZATION OF PD/C CATALYSTS 17
3.4 REPRODUCTION OF THE CATALYST SYNTHESIS BASED ON AKROS C1 20
3.5 HEAT PRETREATMENT OF THE IMPREGNATED CARBON SUPPORT 21
3.6 VARIATION OF THE REDUCTION PARAMETERS 23
3.7 WET IMPREGNATION AT ELEVATED TEMPERATURE 24
3.8 PRETREATMENT OF THE CARBON SUPPORT 25
3.9 TEST OF DIFFERENT CHELATING AGENTS 26
3.10 VARIATION OF THE PALLADIUM LOADING 27
3.11 AMOUNT OF OXALIC ACID 28
3.12 SYNTHESIS OF THE FINAL CATALYST AND SCALE UP 29

4. ANALYTICAL DATA FOR THE CATALYST AND THE CARBON SUPPORTS 35

4.1 EA DATA FOR THE CARBON SUPPORTS 35
4.2 XRF DATA FOR THE CARBON SUPPORTS 36
4.3 ICP-OES DATA OF THE CATALYSTS BASED ON DIFFERENT CARBON SUPPORTS 41
4.4 SEM DATA FOR THE CARBON SUPPORTS AND CATALYSTS 42
4.5 BET DATA FOR CARBON SUPPORTS AND CATALYSTS 55
4.6 XPS DATA FOR THE CATALYSTS 56
4.7 STEM DATA OF DIFFERENT CATALYSTS 67
4.8 CO CHEMISORPTION FOR THE CATALYSTS 73
4.9 ZETA POTENTIAL AND IEP FOR THE CARBON SUPPORTS 74
4.10 DRIFT DATA FOR THE FINAL CATALYSTS PD-C1-OA-O_2 74

5. REACTION MECHANISMS FOR PD/C CATALYSTS 75

6. REFERENCES 76

1. General Information

All liquid reagents were degassed or distilled prior to use and stored under Ar. Chemicals were purchased from Aldrich, TCI, Alfa, Fisher Chemical, Abcr. Ru-Macho-^{i}Pr was purchased from Stem Chemicals. The heterogeneous catalysts presented in Table S2 were

purchased from Aldrich, Strem and Alfa. Carbon Supports tested in Table 2 and Table S3 were supplied by Carbon activated Europe (COC 4x8, COLPA 60, WOS4x8, COL 4x8), Nova Carbons India Private Limited (EcoSorb CE70, EcoSorb CS55, EcoSorb CK1, EcoSorb CK4), AdFiS products GmbH (AFA-4-dot-S, Dopetac sulfo 100. AFA-4-1050-S) and AKROS GmbH (AKROS C1).

Synthesis of heterogenous catalysts was performed under air. All catalytic experiments on the lab scale were carried out under Ar gas atmosphere with exclusion of air. The glassware was dried under vacuum. Autoclaves were dried and evacuated prior to usage.

Gas GC analysis was performed both on: (a) Agilent Technologies 7890A GC system (HP Plot Q/FID–hydrocarbons, Carboxen/TCD-permanent gases, Ar carrier gas). The CO quantification limit is 78 ppm. (b) Agilent Technologies 7890A GC system (HP Plot Q/FID–hydrocarbons, Carboxen/TCD-permanent gases, He carrier gas). The CO quantification limit is 10 ppm.

The liquid and solid products were characterized by ^{1}H NMR and ^{13}C NMR spectroscopy. The NMR spectra were recorded on Bruker Avance 300 (300 MHz) or 400 (400 MHz) NMR spectrometer. ^{13}C NMR-quant measurements were performed with a Bruker AV 400 MHz spectrometer, and the analysis time for each sample is no less than one and a half hours. MestReNova (version 14.0.1-23559) was used for interpreting and processing the NMR spectra.

Elementary analysis (EA) was performed with a Leco TruSpec Micro CHNS Elemental analyzer for the quantification of C, H, N and S. Other elements were quantified *via* Atomic emission spectroscopy (ICP-OES) using Varian/Agilent 715-ES.

XRD powder pattern were recorded on a Panalytical X'Pert θ/2θ -diffractometer equipped with Xcelerator detector using automatic divergence slits and Cu $k\alpha 1/\alpha 2$ radiation (40 kV, 40 mA; $\lambda = 0.15406$ nm, 0.154443 nm). Cu beta-radiation was excluded using a nickel filter foil. Samples were grinded with a ball mill prior to analysis. Finely pestled samples were mounted on silicon zero background holders and data collection were performed with $0.005°s^{-1}$. Obtained intensities were converted from automatic to fixed divergence slits (0.25°) for further analysis. Peak positions and profile were fitted with Pseudo-Voigt function using the HighScore Plus software package (Panalytical). Phase identification was done by using the PDF-2 database of the International Center of Diffraction Data (ICDD).

X-ray fluorescence data have been collected using a Panalytical Epsilon 1 spectrometer operated with an Ag X-ray source. Powdered samples were weighted into the sample holder which was capped with Mylar Foil towards the source/detector. Samples were grinded with a ball mill prior to analysis.

The surface area and porosity were carried out by the N_2 adsorption isotherm using the Brunauer–Emmett–Teller (BET) method on an ASAP 2020 Micromeritics instrument. Pellet samples have been analyzed as presented and were degassed at 200 °C for 6 h to desorb moisture and impurities from their surfaces.

Scanning Electron Microscope (SEM) analysis was performed on a Thermo Fisher Scientific (Hillsboro, USA) Quattro S equipped with a Thermo Fisher Scientific UltraDry 60M Energy Dispersive X-ray Spectrometer (EDXS) for elemental identification. A secondary electron (SE) detector (Everhart-Thornley type) and a backscatter electron (BSE) detector (solid-state detector, segmented) have been used for image acquisition. The microscope was operated at 15 kV acceleration voltage. Pellet samples have been analyzed as presented. No pretreatment was necessary.

The XPS measurements were performed on an ESCALAB 220iXL (Thermo Fischer Scientific) with monochromated Al Kα radiation (E = 1486.6 eV). Samples are prepared as complete pellet on a stainless-steel holder with conductive double-sided adhesive carbon tape. The electron binding energies were obtained without charge compensation leading to a main C 1s peak at around 284.5 eV. For quantitative analysis the peaks were deconvoluted with Gaussian-Lorentzian curves using the software Unifit 2023, the peak areas were divided by the transmission function of the spectrometer and the element specific sensitivity factor of Scofield. The Pd 3d region is deconvoluted assuming an asymmetric Pd(0) peak with a binding energy around 335.2 eV together with a Pd(II) component around 336.9 eV as well as a plasmon feature around 346.8 eV. The asymmetry of the Pd(0) component complicates the discrimination of the two assumed oxidation states. With the used deconvolution model the ratio of Pd(II) is tendentially overemphasized.

The depth profiling is performed in the same machine using EX-05 sputter source (Thermo Fischer Scientific) with an Argon pressure of $2 \cdot 10^{-7}$ mbar with an acceleration voltage of 2 kV, resulting in a sputter current of approximately 2.0 μA.

Scanning transmission electron microscopy (STEM) measurements were performed at 200kV with a probe aberration-corrected JEM-ARM200F (microscope: JEOL, Japan; corrector: CEOS, Germany) using annular bright field (ABF) and high angle annular dark field (HAADF) detectors. The microscope is equipped with a DRY SD60GV (JEOL) energy-dispersive X-ray-spectrometer (EDXS) for chemical analysis.

The outer pellet surface has been scraped off the pellet and was deposed on a holey carbon supported Cu-grid (mesh 300) without any further pretreatment and transferred to the microscope.

H_2 Chemisorption measurement was done using 3Flex apparatus. 160 mg of the sample was inserted in a quartz reactor. The samples were reduced at 200 °C using 5% H2/Ar. The temperature was hold at 200 °C for 30 min. After then the samples were flushed under Ar (50 ml/min) for 60 min. Then the sample was cooled down up to 50 °C and the sample was subjected to CO pulses (20 CO/He) through a dosing loop until no consumption of the CO at the thermal conductivity detector (TCD) was measured. The peak areas obtained via the thermal conductivity detector are used for the calculation of particle size of the metal. A stoichiometry of CO:Pd = 1:1 was assumed in this case.

Zeta potential measurements for the catalyst samples were conducted using a Zetasizer Ultra Red coupled with the MPT-3 multi-purpose titrator (Malvern Panalytical Ltd.). The Zetasizer operates on the principle of Electrophoretic Light Scattering (ELS) employing the M3-PALS technique, ensuring high precision in zeta potential measurement. The MPT-3 titrator was utilized to systematically adjust the pH of the solution during the measurement process. For sample preparation, 5 mg of catalyst was dispersed in 20 mL of distilled water and then sonicated for 5 min. The suspension was subsequently transferred into the cell of the Zetasizer.

Diffuse reflectance Fourier transform infrared spectroscopy (DRIFTS) measurements were carried out using a Praying Mantis High-Temperature Reaction Chamber (Harrick Scientific Products, Inc.). The spectra were recorded with a Nicolet iS 50 FT-IR spectrometer (ThermoFisher) equipped with an MCT detector. The catalyst was diluted with α-Al_2O_3, resulting in a mixture of 95 wt.% Al_2O_3 and 5 wt.% catalyst and then placed in the reaction cell. The cell was purged with Ar flow at room temperature for 20 min before performance measurements. The background spectra were collected on an inert support under Ar flow.

2. Selected State-of-the-Art Catalysts Active in (Reversible) Hydrogen Storage Based on Bicarbonate

The following table gives an overview over homogeneous and heterogeneous catalyst systems applied in reversible hydrogenation/dehydrogenation for the redox system formate/bicarbonate. The number of cycles performed is listed.

Table S1. Selected catalysts active in (reversible) hydrogen storage based on bicarbonate.

**Entry**	**Catalyst**	**Shape**	**Cation**	**p (H_2/CO_2) [bar]**	**T_{opt} [°C]**	**t [h]**	**TON**	**TOF [h^{-1}]**	**Cycles**	**Lit.**
**1**	[$RuCl_2$ (benzene)]$_2$ + dppm	Soluble powder	Na	80 / - (BH)<br>- (FD)	70 (BH)<br>60 (FD)	2 (BH)<br>3 (FD)	1108 (BH)<br>2000 (FD)	N/A<br>2592 (FD)[a]	1[b]	1
**2**	[$RuCl_2$(*m*TPPMS)$_2$]$_2$	Soluble powder	Na	100 / - (BH)<br>- (FD)	N/A<br>80 (FD)	N/A<br>1 (FD)	N/A<br>120	N/A	3	2
**3**	[Ir_2(Cp*)$_2$(H_2O)$_2$(NN)]	Soluble powder	Na, K	20 / 20 (BH)<br>- (FD)	50 (BH)<br>80 (FD)	8 (BH)<br>12 (FD)	153000 (BH)<br>308000 (FD)	15700 (BH)[a]<br>158000 (FD)[a]	2	3
**4**	0.4 wt.% Pd AC	Insoluble powder	K	10 / - (BH)<br>- (FD)	35 (BH)<br>70 (FD)	2 (BH)<br>1 (FD)	N/A	N/A	4[c]	4
**5**	5 wt.% Pd AC	Insoluble powder	K	10 / - (BH)<br>- (FD)	35 (BH)<br>70 (FD)	2 (BH)<br>1 (FD)	N/A	N/A	21[d]	4
**6**	1 wt.% Pd/r-GO	Insoluble powder	K	40 / - (BH)<br>- (FD)	100 (BH)<br>80 (FD)	32 (BH)<br>2 (FD)	7088 (BH)<br>7025 (FD)	N/A<br>11299 (FD)[a]	6	5
**7**	[NiH(PCP)][g]	Soluble powder	Na	55 / - (BH)<br>- (FD)	150 (BH)<br>80 (FD)	20 (BH)<br>3 (FD)	3038 (BH)<br>626 (FD)	N/A	<1	6
**8**	[Ir(COD)(emim)(*m*TPPMS)]	Soluble powder	Cs	100 / - (BH)<br>- (FD)	80	1/3 (BH)<br>N/A	N/A	1983 (BH)<br>15110 (FD)	3	7
**9**	5 wt.% Pd AC	Insoluble powder	NH_4	28 / - (BH)<br>1 bar N_2 (FD)	20 (BH)<br>80 (FD)	15 (BH)<br>1.5 (FD)	1769 (BH)<br>1698 (FD)	N/A	5[e]	4
**10**	[RuH(BH_4)(PNP)]	Soluble powder	K	40 / - (BH)<br>- (FD)	72 (BH)<br>84 (FD)	5 (BH)<br>1.5 (FD)	1225 (BH)<br>994 (FD)	245 (BH)<br>820 (FD)	6[f]	8
**11**	[$RuCl_2$(*m*TPPTS)$_2$]$_2$ + 2 *m*TPPTS	Soluble powder	Cs	100 / - (BH)<br>- (FD)	80 (BH)<br>80 (FD)	40 (BH)<br>1 (FD)	N/A	N/A	5	9
**12**	10 wt.% Pd PdMC	Insoluble powder	Na	40 / - (BH)<br>- (FD)	80	24 (BH)<br>2.5 (FD)	1625 (BH)<br>N/A (FD)	N/A (BH)<br>2562 (FD)	3	10
**13**	$Pd_{0.5}Au_{0.5}$/PdA-rGO	Insoluble powder	K	50 / - (BH)<br>- (FD)	80	2 (BH)<br><1 (FD)	49 (BH)<br>N/A	25 (BH)<br>1630 (FD)	<1	11

Entry	Catalyst	Shape	Cation	p (H_2/CO_2) [bar]	T_{opt} [°C]	t [h]	TON	TOF [h^{-1}]	Cycles	Lit.
**14**	[Ru(acac)$_3$] + Triphos [h]	Soluble powder	Na	40 / 40 (BH)<br>- (FD)	70 (BH)<br>90 (FD)	18 (BH)<br>2 (FD)	750 (BH)<br>N/A (FD)	N/A<br>1200 (FD)	3	12
**15**	[MnBr(CO)$_2$(PNP)][i]	Soluble powder	K	60 / - (BH)<br>- (FD)	90	12	55000 (BH)<br>N/A (FD)	N/A	5	13
**16**	RuMacho^{i}Pr	Soluble powder	K	50 - (BH)<br>- (FD)	90 (BH)<br>60 (FD)	18 (BH)<br>6 (FD)	263200 (BH)<br>9650 (FD)	N/A	40	14

Values were given for the individually investigated hydrogenation bicarbonate (BH) and the dehydrogenation of formate (FD). In the case of the reaction condition being valid for both, BH and FD, it is only noted once. T_{opt} represents the optimal reaction temperature. t [h] represents the reaction time. The number of cycles performed in the publication was noted under cycles. The reaction conditions of the cycles may vary from the ones of the independently investigated reaction conditions for BH and FD. Values were taken directly from the corresponding references or calculated based on experimental data provided within. [a] TOF from the beginning of the reaction. [b]. the solvent was removed *via* evaporation and exchanged in between every half-cycle. Reactivation of the catalysts: [c] after 2 cycles; [d] after 7th, 12th and 17th run; [e] every half cycle. [f] cycles were performed using CO_2 with NaOH as an additive. [g] $OctNMe_2$ was used as an additive in BH. [h] Al(OTf)$_3$ was used as an additive for BH and FD. [i] Glutamic acid was used as an additive. Abbreviations: AC: activated carbon; r-GO: reduced graphene oxide; PdMC: polyaniline-derived mesoporous N-rich carbon materials.

3. Synthesis of Catalysts and Catalytic Screening

A set of heterogeneous catalysts was synthesized based on commercially available carbon supports (powders and pellets). Initially, the catalyst formation was performed based on the publication of Jiang and coworkers.[15] The protocol was adapted and optimized for pellet supports.

The goodness of the catalysts was evaluated based on the activity in the (de)hydrogenation. Therefore, the mass activity was calculated based on the measured metal loading. For more detailed information on the catalytic activity, see 5. Catalyst screening for FD and BH.

If not noted otherwise, the catalytic tests were performed following these descriptions:

General reaction conditions for bicarbonate hydrogenation (BH):

$$\underset{5.4\ \text{mmol}}{KHCO_3} \xrightarrow[\substack{1.5\ \text{mL}\ H_2O \\ 30\ \text{bar}\ H_2 \\ 60\ °C,\ 18\ \text{h}}]{20\ \text{mg catalyst}} KHCO_2 + H_2O$$

BH was performed in parallel reactions in 12 mL vials positioned in a 300 mL autoclave. 5.4 mmol potassium bicarbonate and 20 mg catalyst were evacuated, flushed with Ar and dissolved in 1.5 mL degassed H_2O. The autoclave was flushed with H_2 for three times and pressurized with 30 bar of H_2. The reaction was heated at 60 °C for 18 h. Possible precipitates were dissolved by the addition of 1.5 mL H_2O. 100 µL DMSO was added and the yield was determined by ^1H NMR based on the internal standard DMSO.

General reaction conditions for formate dehydrogenation (FD):

$$\underset{250\ \text{mmol}}{HCOOK} \xrightarrow[\substack{7.5\ \text{mmol}\ K_2CO_3 \\ 25\ \text{mL}\ H_2O, \\ 60\ °C,\ 3\ \text{h}}]{106\ \text{mg catalyst}} H_2 + KHCO_3$$

FD was performed in a double walled glass reactor connected to a manual burette at ambient pressure. The reaction vessel was loaded with 250 mmol potassium formate, 7.5 mmol potassium carbonate and 106 mg catalyst. The vessel was evacuated, flushed with Ar and filled with 25 mL degassed H_2O. The reaction was heated to 60 °C and dehydrogenation was performed for 3 h. Yield is based on the evolution of H_2 (Gas constitution verified *via* GC. CO quantification limit <10 ppm).

3.1 Reaction Setups and Calculations

Following the used setups and calculations for yields, TONs etc. are presented

Reaction setup for FD in an open burette system:

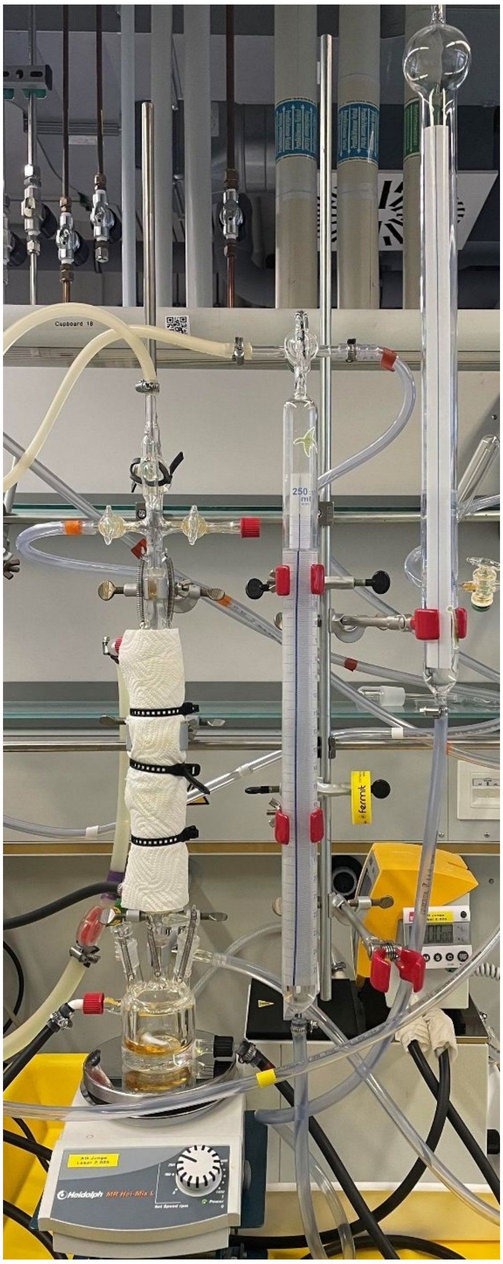

Figure S1. Reaction setup for dehydrogenations. A double-walled reaction vessel is placed on a stirring plate. The vessel is heated with an oil bath and connected to a reflux condenser. The condenser is connected to a burette for manual gas measurements. Gas samples for GC analysis can be taken from the top of the condenser.

Reaction setup for FD in a fixed bed reactor:

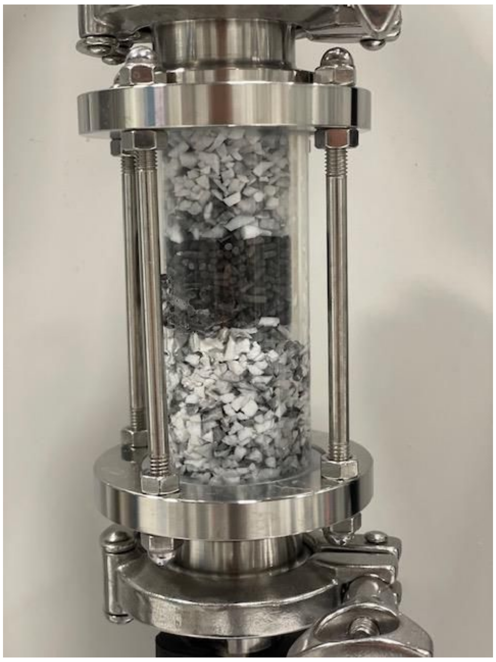

Figure S2. Close up on the reactor packed with PTFE and 10 g of a heterogeneous palladium on carbon catalyst (Pd-C1-OA-O_2). The evolution of gas can be seen on the left-hand side.

Reaction Setup for BH in an autoclave:

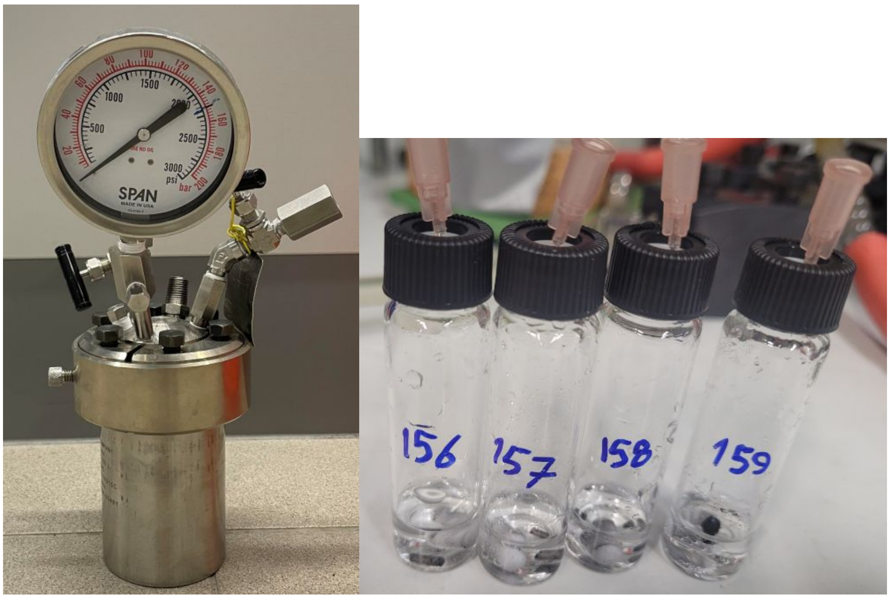

Figure S3. 300 mL autoclaved used for the bicarbonate hydrogenation. Six 12 mL vials can be placed in the autoclave with the help of a sample holder (left). 12 mL vials used for BH in 300 mL autoclave. They are closed with a lid containing a septa. A cannular is punched though the septa to ensure exchange with the gas phase in the autoclave (right).

Calculation of the Gas Production in FD:

Calibrations for the corresponding gases were done with certified commercially available gas mixtures. 5 mL of the collected gas were taken for analysis after the reaction to get the hydrogen percentage (Gc_{H_2}) and carbon dioxide percentage (Gc_{CO_2}) in the total gas.

The amounts of H_2 and CO_2 were calculated based on Eq. (1) and (2):

$$n_{H_2} = \frac{V_{gas} * Gc_{H_2}}{V_{m,H_2,\,25°C}} \quad (1)$$

$$n_{CO_2} = \frac{V_{gas} * Gc_{CO_2}}{V_{m,CO_2,\,25°C}} \quad (2)$$

The turnover number (TON) and turnover frequency (TOF) for the released H_2 were calculated based on Eq. (3) and (4):

$$TON_{H_2} = \frac{n_{H_2}}{n_{cat}} \quad (3)$$

$$TOF_{H_2} = \frac{n_{H_2}}{n_{cat} * t} \quad (4)$$

where V_{gas} is the gas volume corrected by blank volume.

The calculation of $V_{m,H_2,\,25°C}$ and $V_{m,CO_2,\,25°C}$ were carried out using equation (5) and (6):

$$V_{m,H_2,\,25°C} = \frac{RT}{p} + b - \frac{a}{RT} = 24.48\ \frac{L}{mol} \quad (5)$$

$$V_{m,CO_2,\,25°C} = \frac{RT}{p} + b - \frac{a}{RT} = 24.36\ \frac{L}{mol} \quad (6)$$

with:

R: 8.3145 $m^3 \cdot Pa \cdot mol^{-1} \cdot K^{-1}$;

T: 298.15 K;

P: 101325 Pa;

a (H_2): $24.7 \cdot 10^{-3} \cdot Pa \cdot m^6 \cdot mol^{-2}$ and a (CO_2): $36.5 \cdot 10^{-2} \cdot Pa \cdot m^6 \cdot mol^{-2}$;

b (H_2): $26.6 \cdot 10^{-6}\ m^3 \cdot mol^{-1}$ and b (CO_2): $42.7 \cdot 10^{-6}\ m^3 \cdot mol^{-1}$

The $Rate_{gas\ relesae}$ was calculated based on Eq. (7):

$$Rate_{gas\ release} = \frac{V_{gas}}{t} \quad (7)$$

The space-time-yield for FD was calculated based on Eq. (8):

$$\text{space} - \text{time} - \text{yield} = \frac{n_{H_2}}{V_{reactor} * t} \tag{8}$$

where $V_{reactor}$ is the volume of the corresponding reactor.

The mass activity for FD was calculated based on Eq. (9):

$$\text{mass activity FD} = \frac{n_{H_2}}{m_{Pd} * t} \tag{9}$$

Calculation of the Formate Production in BH:

The yield of formate was calculated based on Eq. (10)

$$n_{formate} = n_{DMSO} * Integral_{formate} \tag{10}$$

The mass activity for BH was calculated based on Eq. (11):

$$\text{mass activity BH} = \frac{n_{formate}}{m_{Pd} * t} \tag{11}$$

Calculation of the Power Specific Price (PSP):

The PSP is a purposed indicator for economic analysis. It can be calculated based on Eq. (12):

$$PSP = \frac{m_{cat} * R * T}{p * \dot{V}_{H2} * LHV_{H2} * M} * wt\% * CoP \tag{12}$$

With:

m_{cat}: 0.01 kg;

R: 8.3145 J * $mol^{-1} \cdot K^{-1}$;

T: 293 K;

p: 101325 Pa;

VH_2: 0.006 m^3 * h^{-1};

LHV_{H2}: 33.3 kWh * kg^{-1};

Herein the cost of palladium (CoP) is associated with the hydrogen release performance of the catalyst.

Gas Chromatograms (GC) of the Collected Reaction Gas Mixture in FD:

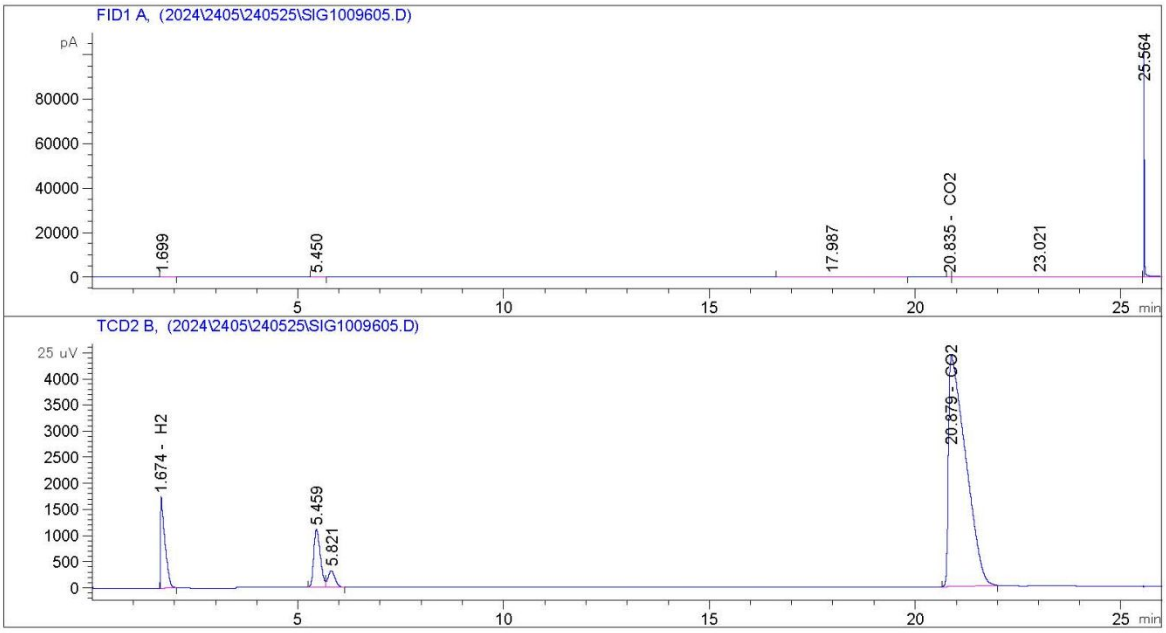


```
=====================================================================
          External Standard Report (Sample Amount is 0!)
=====================================================================

Sorted By             :      Signal
Calib. Data Modified  :      5/22/2024 7:31:35 PM
Multiplier            :      1.0000
Dilution              :      1.0000
Use Multiplier & Dilution Factor with ISTDs

Signal 1: FID1 A,

RetTime  Type     Area      Amt/Area     Amount   Grp   Name
 [min]          [pA*s]                 [Vol. %]
-------|------|----------|----------|----------|--|------------------
  7.644            -          -           -         CO
 14.496            -          -           -         CH4
 20.835 BV       3.70374 5.19459e-2 1.92394e-1     CO2

Totals :                             1.92394e-1

Signal 2: TCD2 B,

RetTime  Type     Area      Amt/Area     Amount   Grp   Name
 [min]         [25 uV*s]               [Vol. %]
-------|------|----------|----------|----------|--|------------------
  1.674 BB    1.23159e4  5.10260e-3   62.84324      H2
  5.000            -          -           -         Ar/N2+O2
  7.644            -          -           -         CO
 14.476            -          -           -         CH4
 20.879 BB    1.28007e5  2.45594e-4   31.43763      CO2

Totals :                               94.28087
```

Figure S4. Example for a GC analysis of the gas collected from FD. CO quantification limit <10 ppm.

^{1}H NMR Spectra of the Reaction Solution in BH:

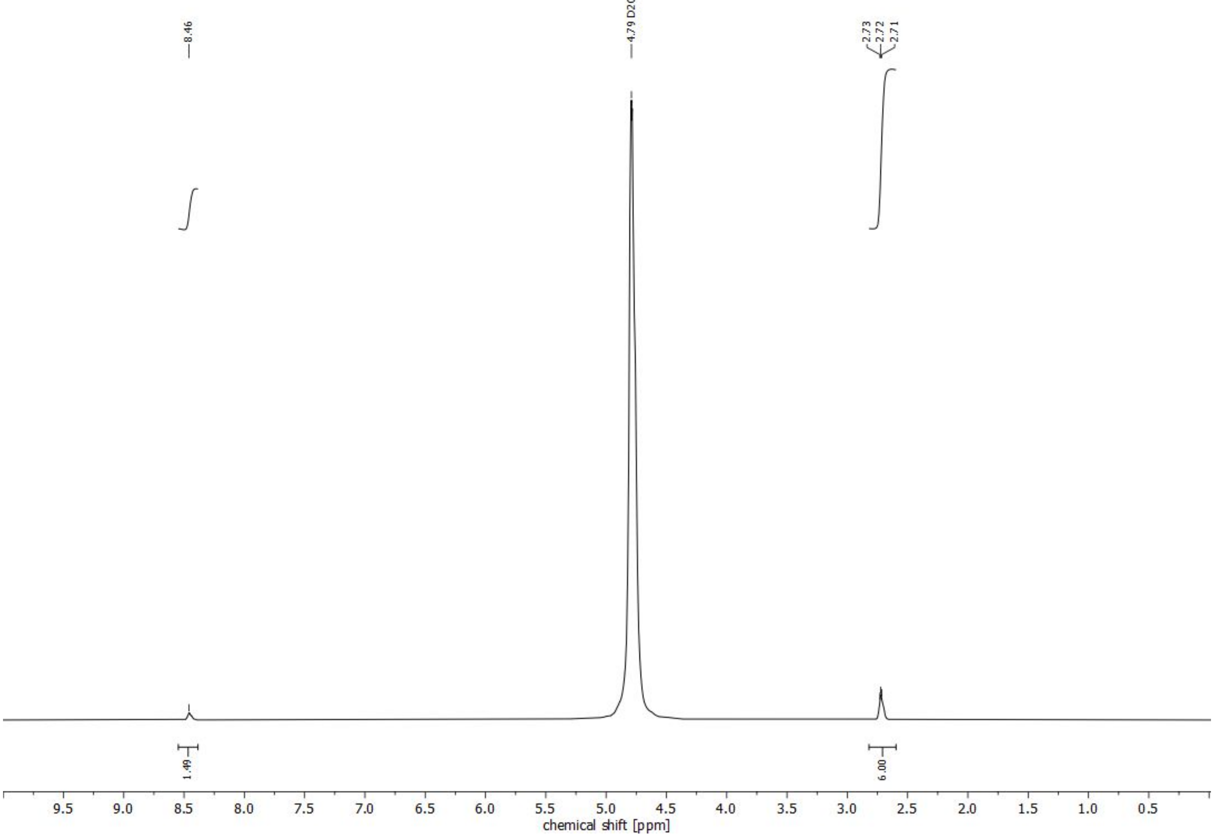

Figure S5. ^{1}H NMR Spectra of a filtered reaction solution. Yields are determined based on the internal standard DMSO.

3.2 Comparison of Commercial Catalyst Systems for BH

A variety of commercially available, homogeneous and heterogeneous catalysts has been tested for BH.

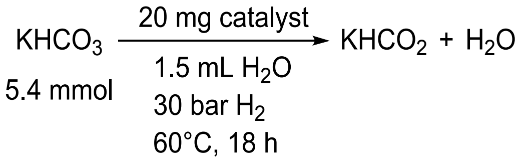

BH was performed in an autoclave equipped with 12 mL vials, if not noted otherwise. 5.4 mmol potassium bicarbonate and 20 mg catalyst were evacuated, flushed with Ar and dissolved in 1.5 mL degassed H_2O. The autoclave was flushed with H_2 for three times and pressurized with 30 bar of H_2. The reaction was heated at the mentioned temperature for 18 h. Possible precipitates were dissolved by the addition of 1.5 mL H_2O. 100 µL DMSO were added and the yield was determined by ^{1}H NMR based on the internal standard DMSO.

Table S2. Screening of commercial homogeneous and heterogeneous catalysts.

**Entry**	**Name**	**Shape**	**Type of catalysis**	**Solvent [mL]**	**T [°C]**	**Yield [%]**	**TON$_{BH}$**
1[a]	Ru-Macho-^{i}Pr	Soluble powder	Homogeneous	triglyme/H_2O (20/5)	90	4	590
2[a]	Ru-Macho-^{i}Pr	Soluble powder	Homogeneous	triglyme/H_2O (15/10)	90	74	9650
3[a]	Ru-Macho-^{i}Pr	Soluble powder	Homogeneous	triglyme/H_2O (12.5/12.5)	90	63	8040
4[a]	Ru-Macho-^{i}Pr	Soluble powder	Homogeneous	triglyme/H_2O (10/15)	90	66	8410
5[a]	Ru-Macho-^{i}Pr	Soluble powder	Homogeneous	triglyme/H_2O (5/20)	90	67	8580
6[a]	Ru-Macho-^{i}Pr	Soluble powder	Homogeneous	triglyme/H_2O (15/10)	75	50	6650
7[a]	Ru-Macho-^{i}Pr	Soluble powder	Homogeneous	triglyme/H_2O (15/10)	60	39	4540
8[b]	Ru-Macho-^{i}Pr	Soluble powder	Homogeneous	H_2O (1.5)	60	20	32
9[b]	5% Ru/C #1	Insoluble powder	Heterogeneous	H_2O (1.5)	60	2	25
10[b]	5% Ru/C #2	Insoluble powder	Heterogeneous	H_2O (1.5)	60	1	10
11[b]	5% Ru/Al_2O_3	Insoluble powder	Heterogeneous	Triglyme/H_2O (1.6/0.8)	90	2	25
12[b]	5% Pt/C #1	Insoluble powder	Heterogeneous	H_2O (1.5)	90	2	22
13[b]	5% Pt/C #2	Insoluble powder	Heterogeneous	H_2O (1.5)	90	0	0
14[b]	5% Pt/C #3	Insoluble powder	Heterogeneous	H_2O (1.5)	90	1	7
15[b]	5% Pd/C #1	Insoluble powder	Heterogeneous	H_2O (1.5)	60	49	1640
16[b]	5% Pd/C #2	Insoluble powder	Heterogeneous	H_2O (1.5)	60	49	1640
17[b]	10% Pd/C #1	Insoluble powder	Heterogeneous	H_2O (1.5)	60	39	1298
18[b]	10% Pd/C #2	Insoluble powder	Heterogeneous	H_2O (1.5)	60	42	1355
19[b]	10% Pd/C #3	Insoluble powder	Heterogeneous	H_2O (1.5)	90	89	1184
20[b]	1% Pd/C #1	Insoluble pellet	Heterogeneous	H_2O (1.5)	60	2	150
21[b]	1% Pd/C #2	Insoluble pellet	Heterogeneous	H_2O (1.5)	60	2	45
22[b]	1% Pd/C #3	Insoluble pellet	Heterogeneous	H_2O (1.5)	60	2	75
23[b]	1% Pd/C #4	Insoluble pellet	Heterogeneous	H_2O (1.5)	60	0	0
24[b]	4% Pd/C #1	Insoluble pellet	Heterogeneous	H_2O (1.5)	60	0	0

General conditions: [a] 135 mmol $KHCO_3$, 10 µmol catalyst, x mL co-solvent, y mL H_2O, 50 bar H_2, T °C, 18 h. Yields and TON values were determined by ^{1}H NMR using DMSO as an internal standard. [b] 5.4 mmol $KHCO_3$, 50 bar H_2, 18 h.

3.3 Initial Optimization of Pd/C catalysts

The first generation of heterogeneous palladium on carbon catalysts were synthesized accordingly.

Initial *in situ* protocol based on the publication of Jiang and coworkers.[15]:

A solution of Na_2PdCl_4 (47.00 µmol) in 10.00 mL deionized water was heated to the mentioned impregnation temperature in a reactor attached to a burette. 95.00 mg of carbon support was added to the solution. After 5 min of stirring at 500 rpm, 5.00 mL of an aqueous solution of citric acid (150.00 µmol, 3 eq), sodium formate (4.20 mmol, 89 eq) and formic acid (5.30 mmol) was added. The reaction vessel was closed, and the evolving gas measured for 3 h. The *in situ* formed catalysts was filtered off, washed with deionized water and dried on air. The dry catalysts were then analyzed by ICP-OES and tested in the hydrogenation, too.

Wet impregnation:

808.00 mg of the support were dried under vacuum for multiple hours, then 40.00 mL degasses water was added. A solution of Na_2PdCl_4 (40.00 µmol, correspond to 5 wt.%, 20.00 mL degassed water) was added dropwise to the support, while stirring at 500 rpm. The reaction was stirred for 12 h at room temperature, filtered, washed three times with water and dried. The dry sample was reduced with 5% H_2 for 2 h at 200 °C (heating 5 °C/min, 2.50 mL H_2 in Ar). Following, the catalysts was analyzed with ICP-OES and tested in the (de)hydrogenation.

Table S3: Initial optimization of the catalyst synthesis using different reductants and carbon supports as well as palladium precursors. The activity in BH and FD is listed based on the measured metal loading.

Entry	Carbon Support	Shape	Catalyst name	Pd Precursor	T_{Imp} [°C]	Synthesis method	Pd_{meas} [wt.%]	Yield BH [%]	TON_{BH}	Mass activity BH [mmol($KHCO_2$)/ mg(Pd)*h]	Gas volume [mL]	H_2:CO_2 [%]	Mass activity FD [mmol(H_2)/ mg(Pd)*h]
1	Vulkan CX-72	Powder	Pd-C13	Na_2PdCl_4	r.t.	*In situ*	4.78	7	240	0.12	23	n.d.	0.31
2	Vulkan CX-72	Powder	6.50Pd-C13	Na_2PdCl_4	60	*In situ*	6.50	7	480	0.27	61	n.d.	1.08
3	AKROS C1	Pellet	3.85Pd-C1	Na_2PdCl_4	60	*In situ*	3.85	4	150	0.09	74	n.d.	0.23
4	AKROS C1	Pellet	3.35Pd-C1	Na_2PdCl_4	60	*In situ*	3.35	13	310	0.06	99	n.d.	0.39
5	AKROS C1	Pellet	1.63Pd-C1	$Pd(NO_3)*2H_2O$	r.t.	Wet imp	1.63	2.5	250	0.02	n.d.	n.d.	n.d.
6	AKROS C1	Pellet	0.83Pd-C1	$Pd(OAc)_2$	r.t.	Wet imp	0.83	0	0	0	35	99:1	0.55
7	AKROS C1	Pellet	Pd-C1	Na_2PdCl_4	r.t.	Wet imp	3.33	30.5	250	0.15	265	99:1	1.04
8	EcoSorb CK1	Pellet	Pd-C5	Na_2PdCl_4	r.t.	Wet imp	3.18	17	130	0.07	175	99:1	0.72
9	EcoSorb CK4	Pellet	Pd-C9	Na_2PdCl_4	r.t.	Wet imp	2.79	5	60	0.03	40	99:1	0.19
10	COL 4x8	Pellet	Pd-C4	Na_2PdCl_4	r.t.	Wet imp	3.94	27	165	0.07	195	99:1	0.65
11	COLPA60	Pellet	Pd-C10	Na_2PdCl_4	r.t.	Wet imp	3.19	0	0	0	95	99:1	0.39
12	WOS 4x8	Pellet	Pd-C6	Na_2PdCl_4	r.t.	Wet imp	3.65	12	105	0.06	19	99:1	0.07
13	COC 4x8	Pellet	Pd-C2	Na_2PdCl_4	r.t.	Wet imp	2.78	18.5	190	0.10	235	99:1	1.11
14	EcoSorb CE55	Pellet	Pd-C7	Na_2PdCl_4	r.t.	Wet imp	2.70	48	90	0.05	389	99:1	1.02
15	EcoSorb CE70	Pellet	Pd-C8	Na_2PdCl_4	r.t.	Wet imp	4.91	44.5	70	0.04	495	99:1	1.32
16	AFA-4-dot-S	Pellet	Pd-C11	Na_2PdCl_4	r.t.	Wet imp	0.38	0	0	0	0	-	0
17	Dopetac Sulfo	Pellet	Pd-C12	Na_2PdCl_4	r.t.	Wet imp	0.11	0	0	0	3	-	0
18	AFA-4-1050-S	Pellet	Pd-C3	Na_2PdCl_4	r.t.	Wet imp	1.74	15	175	0.09	132	99:1	0.99

Measured metal content (Pd_{meas}) is listed; General reaction conditions: BH: 5.40 mmol $KHCO_3$, 20 mg palladium on carbon, 1.50 mL H_2O, 30 bar H_2, 60 °C, 18 h. Yield is determined by ^1H NMR with DMSO as internal standard; FD: 250.00 mmol $KHCO_2$, 7.50 mmol potassium carbonate, 106.00 mg catalyst, 25.00 mL H_2O, 60 °C, 3 h. Yield is based on the evolution of H_2 (Gas constitution verified *via* GC). No CO was detected (CO quantification limit <10 ppm). n.d. = not determined; Imp = impregnation.

The catalyst from Entry 4 (3.35Pd-C1) was tested in a long-term experiment for FD. The experiment was performed in a manual burette. The reaction vessel was loaded with 250 mmol potassium formate, 7.5 mmol potassium carbonate and 106 mg catalyst. The vessel was evacuated, flushed with Ar and filled with 25 mL degassed H_2O. The reaction was heated to 60 °C and dehydrogenation was performed for 64 days. After 32 days, 250 mmol potassium formate were added.

Table S4 Test for long term stability in FD.

**Entry**	**Time [days]**	**Volume [mL]**	**H_2 [%]**	**CO_2 [%]**	**CO [ppm]**
1	3 h	85	> 99	1	n.d.
2	10	1925	99	1	25
3	20	3550	98	2	11
4	24	4005	97	3	17
5	30	4743	96	4	14
6	44	6328	98	2	< 10
7	54	7115	97	3	< 10
8	63	7856	96	4	15

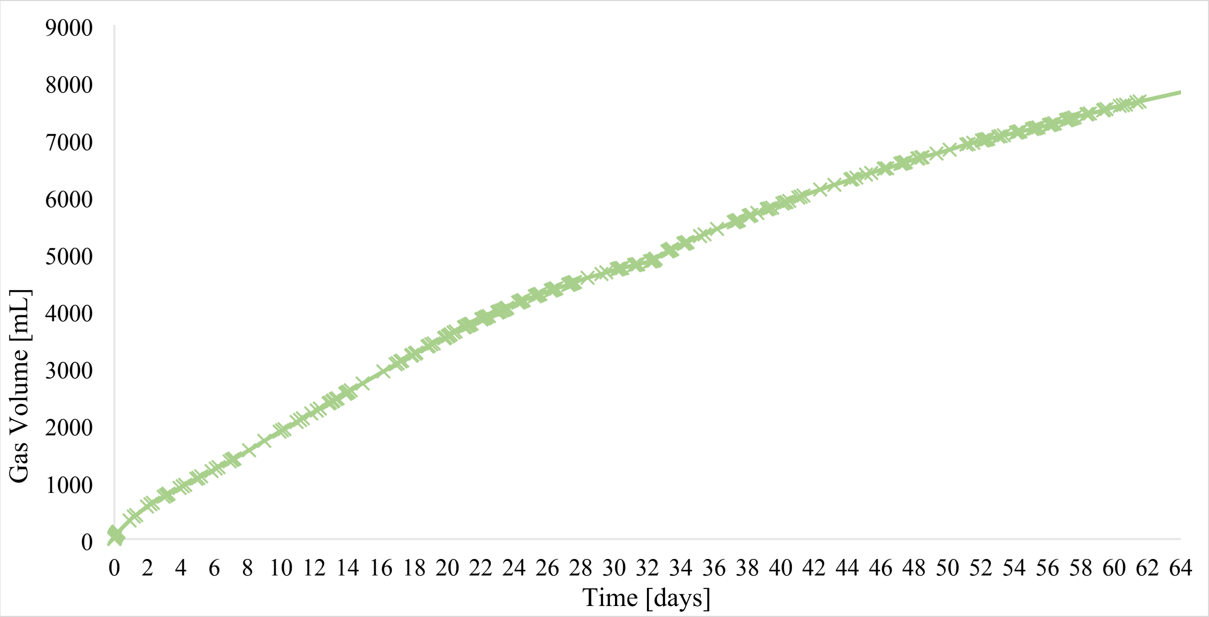

Figure S6. Long term experiment for FD with 3.35Pd-C1.

3.4 Reproduction of the Catalyst Synthesis Based on AKROS C1

General procedure for the synthesis:

808.00 mg of the carbon pellet AKROS C1 were dried under vacuum for multiple hours, then 40.00 mL degasses water was added. A solution of Na_2PdCl_4 (40.00 µmol, correspond to 5 wt.%, 20.00 mL degassed water) was added dropwise to the support, while stirring at 500 rpm. The reaction was stirred for 12 h at room temperature, filtered, washed three times with water and dried. The dry sample was reduced with 5 % H_2 for 2 h at 200 °C (heating 5 °C/min, 2.50 mL H_2 in Ar). Following, the catalysts was analyzed with ICP-OES and tested in the (de)hydrogenation.

Table S5. Reproduction of the initial catalyst Pd-C1. The activity in BH and FD is listed based on the measured metal loading.

Entry	Pd_{meas} [wt.%]	Average Pd [wt.%]	Mass activity BH [mmol($KHCO_2$)/ mg(Pd)*h]	Average Mass activity BH [mmol($KHCO_2$)/ mg(Pd)*h]	Mass activity FD [mmol(H_2)/ mg(Pd)*h]	Average Mass activity FD [mmol(H_2)/ mg(Pd)*h]
1	3.33	3.23	0.15	0.14	1.04	1.41
2	3.14		0.14		0.96	
3	3.23		0.14		2.24	

Measured metal content (Pd_{meas}) is listed; General reaction conditions: BH: 5.40 mmol $KHCO_3$, 20 mg palladium on carbon, 1.50 mL H_2O, 30 bar H_2, 60 °C, 18 h. Yield is determined by ^1H NMR with DMSO as internal standard; FD: 250.00 mmol $KHCO_2$, 7.50 mmol potassium carbonate, 106.00 mg catalyst, 25.00 mL H_2O, 60 °C, 3 h. Yield is based on the evolution of H_2 (Gas constitution verified *via* GC). No CO was detected (CO quantification limit <10 ppm).

3.5 Heat Pretreatment of the Impregnated Carbon Support

General procedure for the synthesis of the second generation of catalyst:

2.42 g carbon pellet support AKROS C1 were stirred in 150.00 mL degassed, deionized water at room temperature. A solution of Na_2PdCl_4 (1.20 mmol, 5 wt.%) in 30.00 mL of degassed, deionized water was prepared. The solution was added dropwise the support over 5 min. The reaction solution was stirred until it cleared up. The impregnated carbon was removed *via* filtration, was washed three times with deionized water and dried at room temperature. Following, 1/3 of the catalysts was calcinated and 1/3 was annilated. After the heat treatment, the catalysts were reduced in a reduction oven with 5 % H_2/Ar (50.00 mL/min) at 200 °C for 2 h (heating rate 5 °C/min). All catalysts were analyzed by ICP-OES.

Table S6. Catalysts based on different heat pretreatments and their (de)hydrogenation results.

Entry	Catalyst name	$T_{Anneling}$ (Ar) [°C]	$T_{Calcination}$ (O_2) [°C]	Pd_{meas} [wt.%]	Pd_{meas} (after 18 h BH) [wt.%]	Pd loss [%]	Yield BH [%]	TON_{BH}	Mass activity BH [mmol($KHCO_2$)/ mg(Pd)*h]	Gas volume [mL]	H_2:CO_2 [%]	Mass activity FD [mmol(H_2)/ mg(Pd)*h]
1	Pd-C1-Ar	200	-	2.73	1.86	32	23	281	0.11	530	> 99:1	2.54
2	Pd-C1-O_2	-	200	2.86	2.33	19	28	316	0.13	615	> 99:1	2.82

Measured metal content (Pd_{meas}) is listed; General reaction conditions: BH: 5.40 mmol $KHCO_3$, 20 mg catalyst, 1.50 mL H_2O, 30 bar H_2, 60 °C, 18 h. Yield is determined by ^1H NMR with DMSO as internal standard; FD: 250.00 mmol $KHCO_2$, 7.50 mmol potassium carbonate, 106.00 mg catalyst, 25.00 mL H_2O, 60 °C, 3 h. Yield is based on the evolution of H_2 (Gas constitution verified *via* GC). No CO was detected (CO quantification limit <10 ppm).

The catalyst from Entry 2 Pd-C1-O_2 was tested in a long-term experiment for FD. The experiment was performed in a manual burette. The reaction vessel was loaded with 250 mmol potassium formate, 7.5 mmol potassium carbonate and 106 mg catalyst. The vessel was evacuated, flushed with Ar and filled with 25 mL degassed H_2O. The reaction was heated to 60 °C and dehydrogenation was performed for 3 days. After 3 days, no palladium was found in the reaction solution meaning that there is no metal leaching.

Table S7. Test for long term stability in FD.

**Entry**	**Time [days]**	**Gas volume [mL]**	**H_2 [%]**	**CO_2 [%]**	**CO [ppm]**
1	1	2030	89	11	< 10
2	2	2665	88	12	< 10
3	3	3235	87	13	16

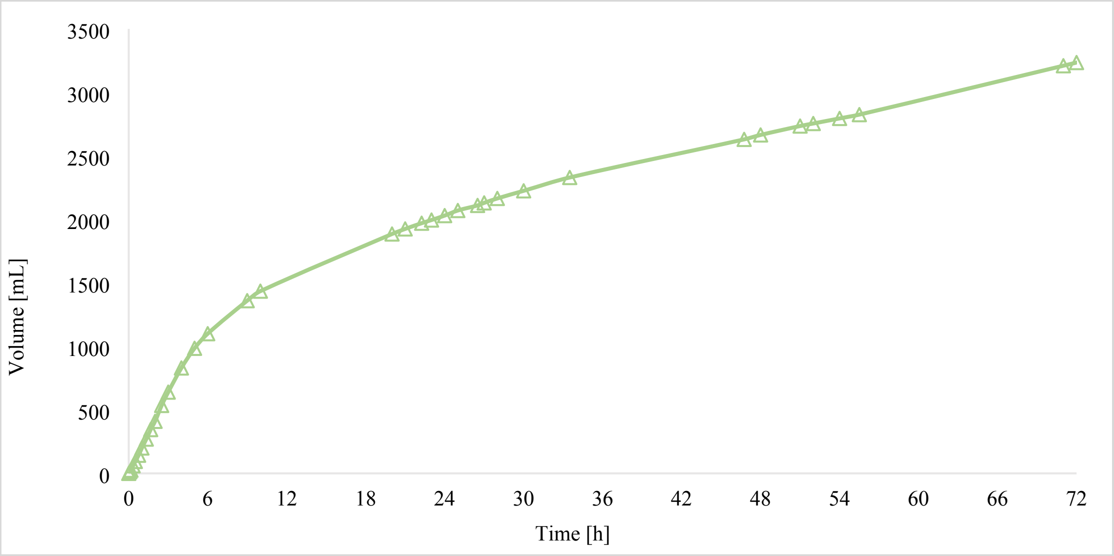

Figure S7. Long term experiment for FD utilizing the calcinated catalyst Pd-C1-O_2.

3.6 Variation of the Reduction Parameters

General procedure for the synthesis of the third generation of catalyst:

808.00 mg carbon pellet support AKROS C1 were stirred in 50.00 mL degassed, deionized water at room temperature. A solution of Na_2PdCl_4 (0.40 mmol, 5 wt.%) was prepared in 10.00 mL of degassed, deionized water. The reaction solution was stirred until it cleared up. The impregnated carbon was removed *via* filtration, washed three times with deionized water and dried at room temperature. Following, the catalysts was reduced in a reduction oven with 5 % H_2/Ar (50.00 mL/min) at the mentioned temperature for a certain time (heating rate 5 °C/min). All catalysts were analyzed by ICP-OES.

Table S8. Catalysts based on different reduction conditions and their (de)hydrogenation results.

Entry	Catalyst name	T_{Red} [°C]	$Time_{Red}$ [h]	Pd_{meas} [wt.%]	Yield BH [%]	TON_{BH}	Mass activity BH [mmol($KHCO_2$)/mg(Pd)*h]	Gas volume [mL]	H_2:CO_2 [%]	Mass activity FD [mmol(H_2) mg(Pd)*h]
1	Pd-C1-600	600	2	3.99	12	90	0.04	96	88:12	0.31
2	Pd-C1-4	200	4	4.01	40	300	0.16	480	99:1	1.57
3	Pd-C1	200	2	3.33	31	250	0.15	265	99:1	1.04
4	Pd-C1-1	200	1	3.97	28	230	0.12	493	>99:1	1.63
5	Pd-C1-100	100	2	3.80	14	115	0.06	375	99:1	1.29
6	Pd-C1-400	400	2	4.23	10	70	0.04	129	99:1	0.41

Measured metal content (Pd_{meas}) is listed; General reaction conditions: BH: 5.40 mmol $KHCO_3$, 20 mg catalyst, 1.50 mL H_2O, 30 bar H_2, 60 °C, 18 h. Yield is determined by ^{1}H NMR with DMSO as internal standard; FD: 250.00 mmol $KHCO_2$, 7.50 mmol potassium carbonate, 106.00 mg catalyst, 25.00 mL H_2O, 60 °C, 3 h. Yield is based on the evolution of H_2 (Gas constitution verified *via* GC). No CO was detected (CO quantification limit <10 ppm).

3.7 Wet Impregnation at Elevated Temperature

General procedure for the synthesis of the fourth generation of catalyst:

808.00 mg carbon pellet support AKROS C1 were stirred in 50.00 mL degassed, deionized water at 60 °C. A solution of Na_2PdCl_4 (0.40 mmol, 5 wt.%) was prepared in 10.00 mL of degassed, deionized water. The solution was added dropwise the support over of 5 min. The reaction solution was stirred until it cleared up. The solvent was evaporated at 60 °C. The catalyst was washed three times with deionized water and dried at room temperature. Following, the catalysts was reduced in a reduction oven with 5 % H_2/Ar (50.00 mL/min) at 200 °C for 2 h (heating rate 5 °C/min). All catalysts were analyzed by ICP-OES.

Table S9. Catalyst synthesized at a different impregnation temperature and the corresponding (de)hydrogenation results.

Catalyst name	T_{Imp} [°C]	Pd_{meas} [wt.%]	Yield BH [%]	TON_{BH}	Mass activity BH [mmol($KHCO_2$)/ mg(Pd)*h]	Gas volume [mL]	H_2:CO_2 [%]	Mass activity FD [mmol(H_2)/ mg(Pd)*h]
Pd-C1-60	60	3.52	25.5	210	0.11	373	99:1	1.39

Measured metal content (Pd_{meas}) is listed; General reaction conditions: BH: 5.40 mmol $KHCO_3$, 20 mg catalyst, 1.50 mL H_2O, 30 bar H_2, 60 °C, 18 h. Yield is determined by ^1H NMR with DMSO as internal standard; FD: 250.00 mmol $KHCO_2$, 7.50 mmol potassium carbonate, 106.00 mg catalyst, 25.00 mL H_2O, 60 °C, 3 h. Yield is based on the evolution of H_2 (Gas constitution verified *via* GC). No CO was detected (CO quantification limit <10 ppm).

3.8 Pretreatment of the Carbon Support

General procedure for the synthesis of the fifth generation of catalyst:

Carbon pretreatment:

Prior to impregnation, the carbon pellet support AKROS C1 was added to a 2 M solution of the mentioned chemical and stirred for 3 days. After that, the support was filtered, washed with deionized water till the washing solution was neutral again and dried on air.

Alternatively, the dry support was calcinated or reduced for 2 h at 200 °C and directly used for impregnation.

Catalyst synthesis:

808.00 mg pretreated carbon support were stirred in 50.00 mL degassed, deionized water at 60 °C. A solution of Na_2PdCl_4 (0.40 mmol, 5 wt.%) was prepared in 10.00 mL of degassed, deionized water. The solution was added dropwise the support over5 min. The reaction solution was stirred until it cleared up. The solvent was evaporated at 60 °C. The catalyst was washed three times with deionized water and dried at room temperature. Following, the catalysts was reduced in a reduction oven with 5 % H_2/Ar (50.00 mL/min) at 200 °C for 2 h (heating rate 5 °C/min). All catalysts were analyzed by ICP-OES.

Table S10. Catalysts based on pretreated AKROS C1 support and their (de)hydrogenation results.

Entry	Catalyst name	Pd_{meas} [wt.%]	Pretreatment of the support	Yield BH [%]	TON_{BH}	Mass activity BH [mmol($KHCO_2$)/mg(Pd)*h]	Gas volume [mL]	H_2:CO_2 [%]	Mass activity FD [mmol(H_2)/mg(Pd)*h]
1	3.12Pd-C1	3.12	Reduction	14	110	0.06	173	> 99:1	0.72
2	3.10Pd-C1	3.10	Oxidation	11	105	0.06	153	99:1	0.64
3	4.21Pd-C1	4.21	H_2O_2	35	220	0.11	350	> 99:1	1.09
4	5.53Pd-C1	5.53	HNO_3	25	150	0.08	528	> 99:1	0.78
5	4.72Pd-C1	4.72	NaOH	49	315	0.17	525	98:2	1.46
6	3.95Pd-C1	3.95	H_3PO_4	31	235	0.12	453	98:2	1.50

Measured metal content (Pd_{meas}) is listed; General reaction conditions: BH: 5.40 mmol $KHCO_3$, 20 mg catalyst, 1.50 mL H_2O, 30 bar H_2, 60 °C, 18 h. Yield is determined by ^{1}H NMR with DMSO as internal standard; FD: 250.00 mmol $KHCO_2$, 7.50 mmol potassium carbonate, 106.00 mg catalyst, 25.00 mL H_2O, 60 °C, 3 h. Yield is based on the evolution of H_2 (Gas constitution verified *via* GC). No CO was detected (CO quantification limit <10 ppm).

3.9 Test of Different Chelating Agents

General procedure for the synthesis of the sixth generation of catalyst:

808.00 mg carbon pellet support AKROS C1 were stirred in 50.00 mL degassed, deionized water at 60 °C. A solution of Na_2PdCl_4 (0.40 mmol, 5 wt.%) and the mentioned amount of additive was prepared in 10.00 mL of degassed, deionized water. The solution was added dropwise the support over 5 min. The reaction solution was stirred until it cleared up. The solvent was evaporated at 60 °C. The catalyst was washed three times with deionized water and dried at room temperature. Following, the catalysts was reduced in a reduction oven with 5 % H_2/Ar (50.00 mL/min) at 200 °C for 2 h (heating rate 5 °C/min). All catalysts were analyzed by ICP-OES.

Table S11. Catalysts based on different additives and their (de)hydrogenation results.

**Entry**	**Catalyst name**	**Additive**	**Amount [eq]**	**Pd_{meas} [wt.%]**	**Yield BH [%]**	**TON_{BH}**	**Mass activity BH [mmol/mg(Pd)*h]**	**Gas volume [mL]**	**H_2:CO_2 [%]**	**Mass activity FD [mmol/mg(Pd)*h]**
1	Pd-C1-CA	Citric Acid	2	2.56	7	80	0.04	95	99:1	0.49
2	Pd-C1-EDTA	EDTA (+ NaOH)	1.1	0.10	4	1345	0.70	40	99:1	5.24
3	3.90Pd-C1-OA	Oxalic Acid	2	3.90	33	275	0.14	395	>99:1	1.33
4[a]	Pd-C1-OA-RT	Oxalic Acid	2	3.98	32	215	0.11	380	99:1	1.25
5	Pd-C1-EN	Ethylenediamine	2	1.71	2	25	0.01	0	-	0
6	Pd-C1-TA	Tartaric Acid	1.1	3.02	15	145	0.08	120	99:1	0.52
7	Pd-C1-MA	Malic Acid	1.1	2.93	19	185	0.10	150	99:1	0.67

Measured metal content (Pd_{meas}) is listed; General reaction conditions: BH: 5.40 mmol $KHCO_3$, 20 mg catalyst, 1.50 mL H_2O, 30 bar H_2, 60 °C, 18 h. Yield is determined by ^{1}H NMR with DMSO as internal standard; FD: 250.00 mmol $KHCO_2$, 7.50 mmol potassium carbonate, 106.00 mg catalyst, 25.00 mL H_2O, 60 °C, 3 h. Yield is based on the evolution of H_2 (Gas constitution verified *via* GC). No CO was detected (CO quantification limit <10 ppm). [a] Impregnation performed at room temperature.

3.10 Variation of the Palladium Loading

General procedure for the synthesis of the seventh generation of catalyst:

808.00 mg AKROS C1 were stirred in 50.00 mL degassed, deionized water at 60 °C. A solution of Na_2PdCl_4 (1-5 wt.%) and oxalic acid (2 eq) was prepared in 10.00 mL of degassed, deionized water. The solution was added dropwise the support over 5 min. The reaction solution was stirred until it cleared up. The solvent was evaporated at 60 °C. The catalyst was washed three times with deionized water and dried at room temperature. Following, the catalysts was reduced in a reduction oven with 5 % H_2/Ar (50.00 mL/min) at 200 °C for 2 h (heating rate 5 °C/min). All catalysts were analyzed by ICP-OES.

Table S12. Catalysts based on different metal loadings and their (de)hydrogenation results.

**Entry**	**Catalyst name**	**Na_2PdCl_4 [µmol]**	**Pd_{theo} [wt.%]**	**Pd_{meas} [wt.%]**	**Pd deviation [%]**	**Yield BH [%]**	**TON_{BH}**	**Mass activity BH [mmol($KHCO_2$)/mg(Pd)*h]**	**Gas volume [mL]**	**H_2:CO_2 [%]**	**Mass activity FD [mmol/mg(Pd)*h]**
1	3.90Pd-C1-OA	400	5.00	3.90	22	33	275	0.14	395	>99:1	1.33
2	3.19Pd-C1-OA	320	4.00	3.19	20	40	375	0.20	313	>99:1	1.28
3	2.62Pd-C1-OA	240	3.00	2.62	13	24	285	0.15	443	>99:1	2.21
4	1.60Pd-C1-OA	160	2.00	1.60	20	20	365	0.19	338	>99:1	2.76
5	0.82Pd-C1-OA	80	1.00	0.82	20	14	550	0.29	161	>99:1	2.57

Theoretical metal content (Pd_{theo}) and measured content (Pd_{meas}) are listed; General reaction conditions: BH: 5.40 mmol $KHCO_3$, 20 mg catalyst, 1.50 mL H_2O, 30 bar H_2, 60 °C, 18 h. Yield is determined by ^1H NMR with DMSO as internal standard; FD: 250.00 mmol $KHCO_2$, 7.50 mmol potassium carbonate, 106.00 mg catalyst, 25.00 mL H_2O, 60 °C, 3 h. Yield is based on the evolution of H_2 (Gas constitution verified *via* GC).No CO was detected (CO quantification limit <10 ppm).

3.11 Amount of Oxalic Acid

General procedure for the synthesis of the eight generation of catalyst:

808.00 mg carbon pellet support AKROS C1 were stirred in 50.00 mL degassed, deionized water at 60 °C. A solution of Na_2PdCl_4 (0.16 mmol, 2 wt.%) and the mentioned amount of oxalic acid was prepared in 10.00 mL of degassed, deionized water. The solution was added dropwise the support over 5 min. The reaction solution was stirred until it cleared up. The solvent was evaporated at 60 °C. The catalyst was washed three times with deionized water and dried at room temperature. Following, the catalysts was reduced in a reduction oven with 5 % H_2/Ar (50.00 mL/min) at 200 °C for 2 h (heating rate 5 °C/min). All catalysts were analyzed by ICP-OES.

Table S13. Catalysts based on different amounts of additive and their (de)hydrogenation results.

**Entry**	**Catalyst name**	**Oxalic acid [eq]**	**Pd_{theo} [wt.%]**	**Pd_{meas} [wt.%]**	**Yield BH [%]**	**TON_{BH}**	**Mass activity BH [mmol($KHCO_2$)/mg(Pd)*h]**	**Gas volume [mL]**	**H_2:CO_2 [%]**	**Mass activity FD [mmol(H_2)/mg(Pd)*h]**
1	1.21Pd-C1-OA	0	2.00	1.21	16	380	0.20	130	>99:1	0.58
2	1.31Pd-C1-OA	1	2.00	1.31	30	645	0.34	395	99:1	3.95
3	1.60Pd-C1-OA	2	2.00	1.60	24	285	0.15	443	>99:1	2.21
	1.42Pd-C1-OA	4	2.00	1.42	29	605	0.32	400	99:1	3.69

Theoretical metal content (Pd_{theo}) and measured content (Pd_{meas}) are listed; General reaction conditions: BH: 5.40 mmol $KHCO_3$, 20 mg catalyst, 1.50 mL H_2O, 30 bar H_2, 60 °C, 18 h. Yield is determined by ^{1}H NMR with DMSO as internal standard; FD: 250.00 mmol $KHCO_2$, 7.50 mmol potassium carbonate, 106.00 mg catalyst, 25.00 mL H_2O, 60 °C, 3 h. Yield is based on the evolution of H_2 (Gas constitution verified *via* GC). No CO was detected (CO quantification limit <10 ppm).

The pH was only slightly affected by the addition of oxalic acid to the impregnation solution. A solution of Na_2PdCl_4 (0.16 mmol, correspond to 2 wt.%) in 10.00 mL of deionized water has a pH of 3-4. Pure oxalic acid in 10.00 mL of water corresponds to a pH of 2-3. The combination of 2 wt.% Palladium and 2 eq of oxalic acid results in a pH of 2-3.

3.12 Synthesis of the Final Catalyst and Scale Up

General procedure for the synthesis of the ninth generation of catalyst:

5.00 g carbon pellet support AKROS C1 were stirred in 95.00 mL degassed, deionized water at 60 °C. A solution of Na_2PdCl_4 (1.00 mmol, 2 wt.%) and 2 eq oxalic acid (1.98 mmol) was prepared in 30.00 mL of degassed, deionized water. The solution was added dropwise the support over 20 min. The reaction solution was stirred until it cleared up. The solvent was evaporated at 60 °C. The catalyst was washed three times with deionized water and dried at room temperature. Following, the catalysts was calcinated for 2 h at 200 °C, then reduced in a reduction oven with 5 % H_2/Ar (50.00 mL/min) at 200 °C for 2 h (heating rate 5 °C/min).

Table S14. Catalysts based on different amounts of additive and their (de)hydrogenation results.

Catalyst name	Pd_{meas} [wt.%]	Yield BH [%]	TON_{BH}	Mass activity BH [mmol($KHCO_2$)/ mg(Pd)*h]	Gas volume [mL]	H_2:CO_2 [%]	Mass activity FD [mmol(H_2)/ mg(Pd)*h]
Pd-C1-OA-O_2	1.81	35	570	0.30	445	97:3	3.22

Measured metal content (Pd_{meas}) is listed; General reaction conditions: BH: 5.40 mmol $KHCO_3$, 20 mg catalyst, 1.50 mL H_2O, 30 bar H_2, 60 °C, 18 h. Yield is determined by ^{1}H NMR with DMSO as internal standard; FD: 250.00 mmol $KHCO_2$, 7.50 mmol potassium carbonate, 106.00 mg catalyst, 25.00 mL H_2O, 60 °C, 3 h. Yield is based on the evolution of H_2 (Gas constitution verified *via* GC). No CO was detected (CO quantification limit <10 ppm).

General procedure for the scale up:

10.00 g carbon pellet support AKROS C1 were stirred in 190.00 mL degassed, deionized water at 60 °C. A solution of Na_2PdCl_4 (4.95 mmol, 2 wt.%) and oxalic acid (2 eq) was prepared in 60.00 mL of degassed, deionized water. The solution was added dropwise to the support over 15 min. The reaction solution was stirred until it cleared up. The solvent was evaporated at 60 °C. The catalyst was washed three times with deionized water and dried at room temperature. Following, the catalysts was calcinated for 2 h at 200 °C, then reduced in a reduction oven with 5 % H_2/Ar (50.00 mL/min) at 200 °C for 2 h (heating rate 5 °C/min). The synthesis was repeated multiple times and the different catalyst batches were combined. ICP-OES resulted in a meal loading of 1.56-1.59 wt% Pd.

Experiments on a Batch Scale:

The final catalyst has been tested for (de)hydrogenation on a bigger scale. First of all, both half cycles have been performed on the same scale. Namely, 106 mg Pd-C1-OA-O_2, 250 mmol of the corresponding (loaded) carrier, 25.0 mL of degassed water. Both reactions were carried out at 60 °C for 18 h. The catalyst was grinded for the batch reactions.

BH was monitored *via* the pressure drop during the reaction. Possible precipitates were dissolved by the addition of 25.0 mL H_2O. 1000 µL DMSO were added and the yield was determined by ^{1}H NMR based on the internal standard DMSO. The pressure drop can be seen below in Figure S8. The results are shown in Table S15.

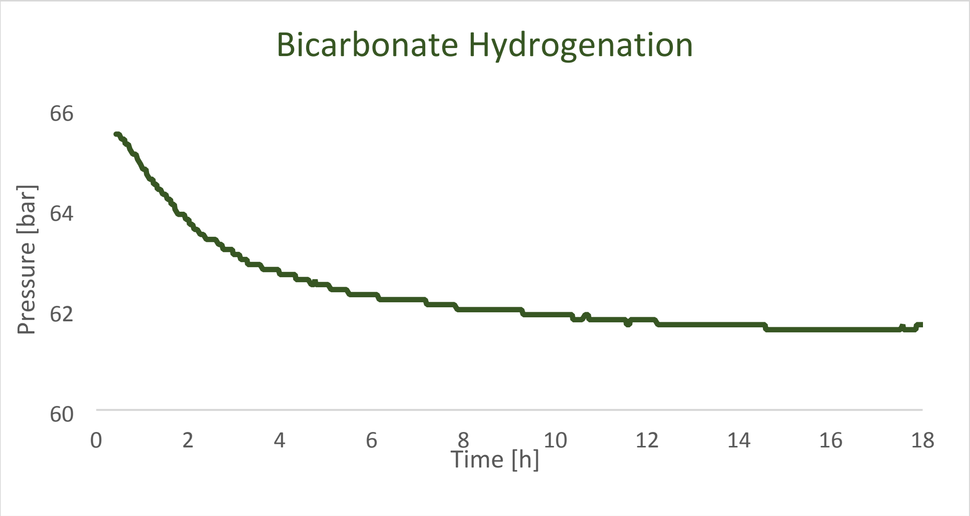

Figure S8. BH for 18 h monitored *via* pressure drop utilizing catalyst Pd-C1-OA-O_2.

FD was performed in an automatic burette. The reaction vessel was loaded with 250.0 mmol potassium formate, 7.5 mmol potassium carbonate and 106 mg catalyst. The vessel was evacuated, flushed with Ar and filled with 25 mL degassed H_2O. The reaction was heated to 60 °C and dehydrogenation was performed for 18 h. Yield is based on the evolution of H_2 (Gas constitution verified *via* GC). No CO was detected (CO quantification limit <10 ppm). The gas evolution can be seen below in Figure S9. The results are shown in Table S15.

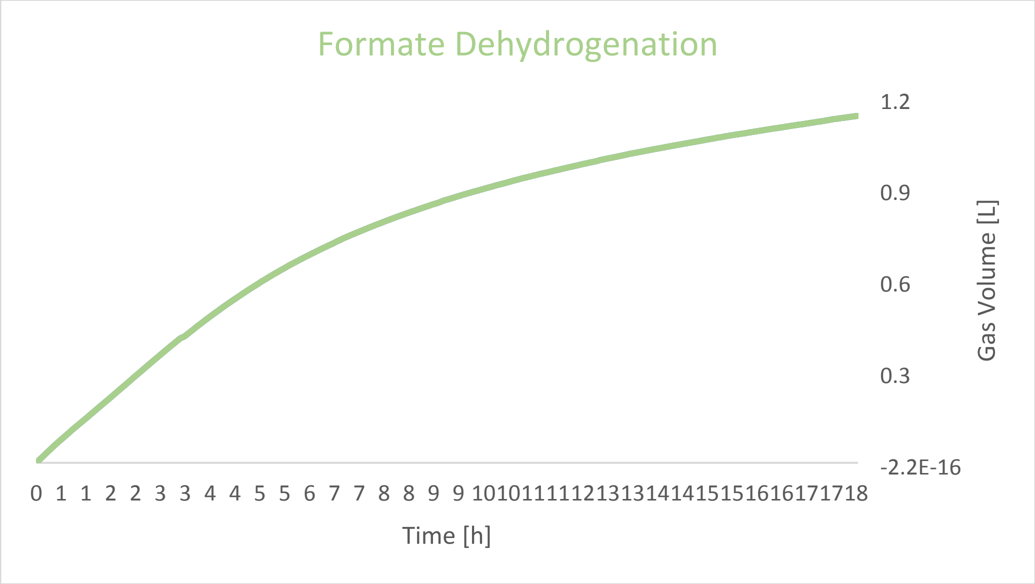

Figure S9. FD for 18 h monitored *via* gas evolution in a manual burette utilizing catalyst Pd-C1-OA-O_2. Gas constitution verified *via* GC.

Table S15. Results for (de)hydrogenation with the final catalyst Pd-C1-OA-O_2 on a bigger scale.

**Pd_{meas} [wt.%]**	**Yield BH [%]**	**TON_{BH}**	**Mass activity BH [mmol($KHCO_2$)/ mg(Pd)*h]**	**Gas volume [L]**	**H_2:CO_2 [%]**	**TOF_{FD} [h^{-1}]**	**Mass activity FD [mmol(H_2)/ mg(Pd)*h]**
1.81	23.02	2550	1.67	1.15	92:8	3261	1.39

.

Next, the recycling of the catalysts was investigated for FD in an automatic burette. Therefore, the reaction vessel was loaded with 200 mg Pd-C1-OA-O_2 (grinded), 200.0 mmol potassium formate, 15.0 mmol potassium carbonate and 50.0 mL of degassed water. The reaction was heated to 60 °C and dehydrogenation was performed for 73 h. The catalyst was filtered, washed, dried and reused for a second dehydrogenation step. Yield is based on the evolution of H_2 (Gas constitution verified *via* GC). The gas evolution can be seen below in Figure S10. The results are shown in Table S16.

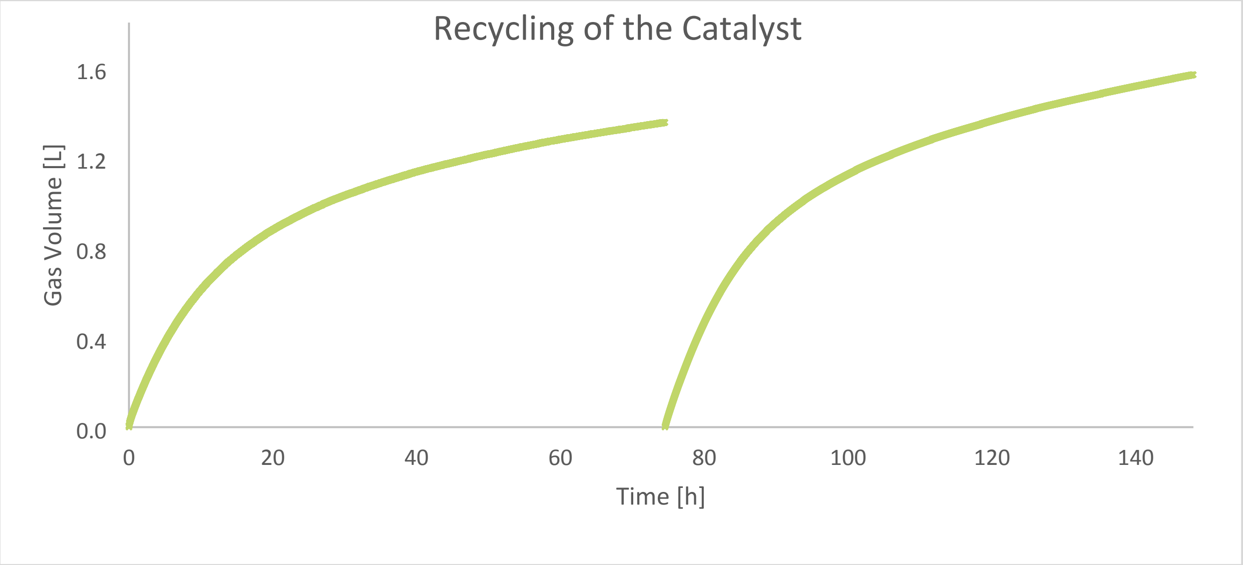

Figure S10. Recycling of the catalysts Pd-C1-OA-O_2 in FD. Each experiment was performed for 73 h. Gas constitution verified *via* GC.

Table S16. Recycling of the catalyst in FD.

**Run No.**	**Gas volume [L]**	**H_2:CO_2 [%]**	**CO [ppm]**	**Mass activity FD [mmol(H_2)/mg(Pd)*h]**
1	1.36	90:10	11	0.21
2	1.57	93:7	18	0.25

Recycling was investigated for BH, too. Therefore, a 300 mL autoclave was loaded with 200 mg Pd-C1-OA-O_2 (grinded), 200.0 mmol potassium bicarbonate and 50.0 mL of degassed water. The reaction was pressurized with 60 bar H_2 and heated to 60 °C for 18 h. The catalyst was filtered, washed, dried and calcinated for 2 h at 200 °C. Then, the catalyst was reduced (5 % H_2, 2 h, 200 °C) and reused for a second hydrogenation step.

Table S17. Recycling of the catalyst in BH.

**Run No.**	**Yield BH [%]**	**TON_{BH}**	**Mass activity BH [mmol($KHCO_2$)/ mg(Pd)*h]**
1	24	1570	0.82
2	14	1650	0.86

FD in a fixed bed reactor:

For FD in a fixed bed reactor, 10 g of catalyst were used. A 4 M $KHCO_2$ was constantly pumped from bottom to top though the reactor with a volume flow of 0.03 L/min. The reactor has an inner diameter of 35 mm. The solution was heated to 60 °C (run 1-6) or 80 °C (run 7-11) and the reaction was performed for 10 h. Gas constitution was verified via GC every two hours. No CO was detected (CO quantification limit <10 ppm) except at the end of the 10th run. After 10 h the catalyst was washed with water at 60 °C and dried on air. Starting with the 6th run additional reactivation methods were tested. Metal leaching was analyzed via ICP after every run and no metal was found in the reaction solution.

The following reactivation methods were tested:

- before the 6th run: calcination (2 h, 200 °C), then reduction (2 h, 200 °C)
- during the 7th run: higher reaction temperature
- before the 9th run: additional washing with diluted H_2O_2
- before the 10th run: additional washing diluted HNO_3.

The gas evolution of the separate runs is shown in Figure S11. The gas analysis is shown in Table S18 and the ICP-OES in Table S19:

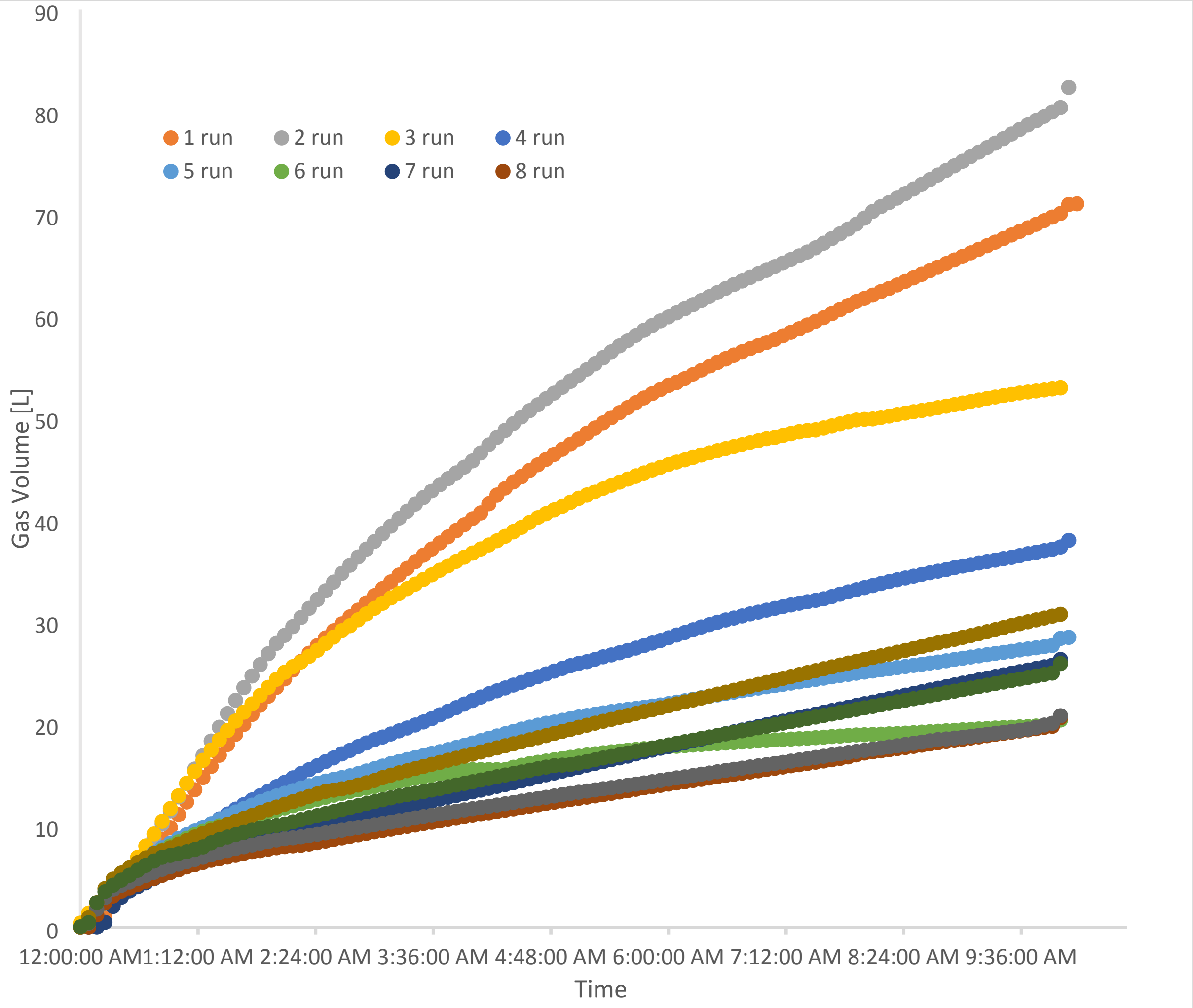

Figure S11. FD with Pd-C1-OA-O_2 in a fixed bed reactor. 10 g of catalyst were initially used. A 4 M $KHCO_2$ was constantly pumped from bottom to top though the reactor with a volume flow of 0.03 L/min. The solution was heated to 60 °C (run 1-6) or 80 °C (run7-11) and the reaction was performed for 10 h. Gas constitution was verified via GC. No CO was detected (CO quantification limit <10 ppm) except at the end of run 10. After 10 h the catalyst was washed with water and dried on air.

Table S18. Gas analysis for FD in a fixed bed reactor.

Entry	Run	Time [h]	H_2 [%]	CO_2 [%]	CO [ppm]
1	1	2	89	11	n.d.
2	1	4	86	14	n.d.
3	1	6	85	15	n.d.
4	1	8	84	16	n.d.
5	1	10	83	17	n.d.
6	2	2	89	11	n.d.
7	2	4	85	15	n.d.
8	2	4	84	16	n.d.
9	2	6	80	20	n.d.
10	2	8	82	18	n.d.
11	2	10	81	19	n.d.
12	3	1	91	9	n.d.
13	3	2	87	13	n.d.
14	3	4	83	17	n.d.
15	3	6	82	18	n.d.
16	3	8	82	18	n.d.
17	3	10	84	16	n.d.
18	4	2	87	13	n.d.
19	4	4	83	17	n.d.
20	4	6	82	18	n.d.
21	4	8	82	18	n.d.
22	4	10	81	19	n.d.
23	5	2	83	17	n.d.
24	5	4	81	19	n.d.
25	5	6	81	19	n.d.
26	5	8	82	18	n.d.
27	5	10	82	18	n.d.
28	6	2	84	16	n.d.
29	6	4	83	17	n.d.
30	6	6	84	16	n.d.
31	6	8	86	14	n.d.
32	6	10	85	15	n.d.
33	7	2	80	20	n.d.
34	7	4	79	21	n.d.
35	7	6	80	20	n.d.
36	7	8	79	21	n.d.
37	7	10	80	20	n.d.
38	8	2	76	24	n.d.
39	8	4	80	20	n.d.
40	8	6	79	21	n.d.
41	8	8	71	29	n.d.
42	8	10	80	20	n.d.
43	9	2	78	22	n.d.
44	9	4	80	20	n.d.
45	9	6	80	20	n.d.
46	9	8	81	19	n.d.
47	9	10	70	30	n.d.
48	10	2	80	20	n.d.
49	10	5	79	21	n.d.
50	10	10	77	23	255
51	11	2	77	23	n.d.
52	11	5	80	20	n.d.
53	11	10	79	21	n.d.

Table S19. Elementary analysis of fresh and used catalysts as well as reaction solution

Entry	Sample	Pd	K	Unit
1	fresh catalyst	1.56	-	[wt.%]
2	catalyst after 10h FD	1.72	1.81	[wt.%]
3	reaction solution after 10h FD	-	153.47	[mg/L]
4	catalyst after second FD	1.57	1.73	[wt.%]
5	reaction solution after second FD	-	179.48	[mg/L]
6	catalyst after third FD	1.61	-	[wt.%]
7	reaction solution after third FD	-	119.55	[mg/L]

After 10 h the catalyst was washed with water and dried. Ca 200 mg of the dried, used catalyst were utilized for ICP analysis. After the third run the catalyst was washed, then was soaked in water over night, filtered and dried on air.

4. Analytical Data for the Catalyst and the Carbon Supports

Following the performed analytical results for the optimization of the synthesis of shell coated palladium on carbon catalysts are listed according to the used method. Depending on the method, catalysts have been analyzed as pellets or grinded by ball mill and analyzed as powders. Information about the pretreatment is given for every analytical method.

4.1 EA Data for the Carbon Supports

EA data for selected pellet carbon supports for the catalysts shown in Table 2 (or Table S3). They are sorted in the same manner as Table 2 according to the activity of the corresponding catalysts in the (de)hydrogenation. All catalysts have been grinded with a ball mill prior to measurement and analyzed as powders.

Table S20. Elementary analysis of selected carbon pellet supports presented in Table 2.

**Support**	**C [%]**	**H [%]**	**N [%]**
AKROS C1	91.375	0.2679	0
COC 4 x 8	88.4	0.1771	0
AFA-4-dot S	77.13	0.16915	0.2807
COL 4 x 8	74.755	0.1621	0.0855
EcoSorb CK1	91.675	0.12395	0
WOS 4 x 8	78.45	0.56175	0.3193
EcoSorb CE55	91.06	0.13525	0
EcoSorb CE70	90.255	0.1535	0
EcoSorb CK4	91.69	0.1356	0
COLPA 60	72.335	0.2066	0.05485
AFA-4-1050-S	89.68	0.10795	0.03085

4.2 XRF Data for the Carbon Supports

XRF data for selected pellet carbon supports for the catalysts shown in Table 2. They are sorted in the same manner according to the activity of the corresponding catalysts in the (de)hydrogenation. All catalysts have been grinded with a ball mill prior to measurement and analyzed as powders. The results are presented in Table S21 and S22.

Table S21. XRF analysis of selected carbon supports. All elements were analyzed as oxides.

**Carbon Support**	**AKROS C1**	**COC 4x8**	**AFA-4-1050-S**	**COL 4 x 8**	**EcoSorb CK1**	**WOS 4 x 8**
Compound	*Conc [%]*	*Conc [%]*	*Conc [%]*	*Conc [%]*	*Conc [%]*	*Conc [%]*
**C**	91	88.4	89.68	74.755	92	78.45
**H**	0.268	0.177	0.108	0.162	0.124	0.562
**N**	0.000	0	0.031	0.086	0.000	0.319
**O**	0.000	0	0	0	0.000	0
**Mg**	0	0	0	0	0	0
**Al**	0	0	1.302	6.797	0	0
**Si**	3	1.032	3.071	14.093	2	1.685
**P**	4.5	0.406	0.131	0.21	0.434	0.489
**S**	0.266	0.199	1.436	2.087	0.161	0.733
**Cl**	0	0.482	0	0	0.121	0.993
**K**	0.09686	7.649	0.03868	0.43	5	1.937
**Ca**	0.157	0.289	1.125	0.177	0.353	12.878
**Sc**	0.00093	0	0	0	0	0
**Ti**	0.08171	0.805	0.496	0.625	0.238	0.94
**V**	0.00221	0	0.00786	0.01751	0	0
**Cr**	0.01567	0.00087	0	0.00538	0	0
**Mn**	0.0359	0.00876	0.01957	0.00235	0.00806	0.0637
**Fe**	0.356	0.157	2.055	0.35	0.142	0.4
**Co**	0	0	0	0.00676	0	0
**Ni**	0.0066	0	0.00991	0.0156	0	0.00129
**Cu**	0.0048	0.08465	0.02589	0.02423	0.03243	0.07621
**Zn**	0.0202	0.05278	0.02353	0.01656	0.02897	0.05211
**Ga**	0	0	0.00407	0.00205	0	0
**Ge**	0	0	0.00155	0.00115	0	0
**As**	0	0	0.00161	0.00152	0	0
**Br**	0	0.00415	0	0	0	0.03309
**Se**	0	0	0.00088	0	0	0
**Rb**	0	0.02983	0.00049	0.00521	0.03445	0.01055
**Sr**	0	0.00568	0.04479	0.01503	0.00803	0.243
**Y2**	0	0	0.0049	0.00883	0.0018	0
**Zr**	0.09987	0.196	0.359	0.08536	0.155	0.131
**Ba**	0	0	0	0	0	0
**Ce**	0	0.01832	0.02183	0.02015	0	0
**Hf**	0	0.00339	0	0	0	0.00376
**Pb**	0	0	0	0	0	0

Table S22. XRF analysis of selected carbon supports. All elements were analyzed as oxides.

**Carbon Support**	**EcoSorb CE55**	**EcoSorb CE70**	**EcoSorb CK4**	**COLPA 60**	**AFA-4-dot S**
Compound	*Conc [%]*	*Conc [%]*	*Conc [%]*	*Conc [%]*	*Conc [%]*
**C**	91.06	90.255	91.69	72.335	77.13
**H**	0.135	0.154	0.136	0.207	0.169
**N**	0	0	0	0.055	0.281
**O**	0	0	0	0	0
**Mg**	0	0.322	0	0	0
**Al**	0	0	0	0	3.059
**Si**	0.918	1.292	1.419	0.876	3.989
**P**	0.144	0.229	0.437	0.334	0.435
**S**	0.593	0.743	0.242	0.981	1.579
**Cl**	0.06448	0.113	0.15	0.596	0.644
**K**	1.852	2.355	4.99	16.181	7.611
**Ca**	1.168	0.468	0.353	7.193	1.873
**Sc**	0	0	0	0	0
**Ti**	1.622	2.757	0.179	0.248	0.245
**V**	0.02896	0	0.00152	0.00122	0.01846
**Cr**	0.08646	0.00212	0	0	0
**Mn**	0.01914	0.00783	0.00607	0.297	0.03887
**Fe**	1.195	0.435	0.127	0.415	2.35
**Co**	0	0	0.00131	0	0
**Ni**	0.00756	0.00223	0.00147	0	0.00894
**Cu**	0.673	0.2	0.02121	0.02191	0.02457
**Zn**	0.371	0.12	0.02205	0.02015	0.01471
**Ga**	0	0.00182	0	0	0.00109
**Ge**	0	0.00679	0	0	0.00055
**As**	0.003	0.00803	0	0	0.00084
**Br**	0.00143	0.00182	0.00112	0.00203	0.00766
**Se**	0	0	0	0	0
**Rb**	0.00752	0.00679	0.03096	0.04856	0.01635
**Sr**	0.01554	0.00803	0.0045	0.03272	0.185
**Y2**	0	0	0.00188	0.00083	0.0078
**Zr**	0.03477	0.446	0.186	0.08153	0.138
**Ba**	0	0	0	0.07198	0.166
**Ce**	0	0.06963	0	0	0.00583
**Hf**	0	0.00919	0	0	0
**Pb**	0	0.00231	0	0	0

PXRD Data for the Catalysts

Catalysts at different stages of the synthesis optimization have been analyzed with PXRD. All catalysts have been grinded with a ball mill prior to measurement.

PXRD Data of the Catalysts from the Carbon Support Screening

Following are the PXRD plots for the catalysts based on different carbon pellet supports shown in Table 2. They are sorted in the same manner according to their activity in the (de)hydrogenation.

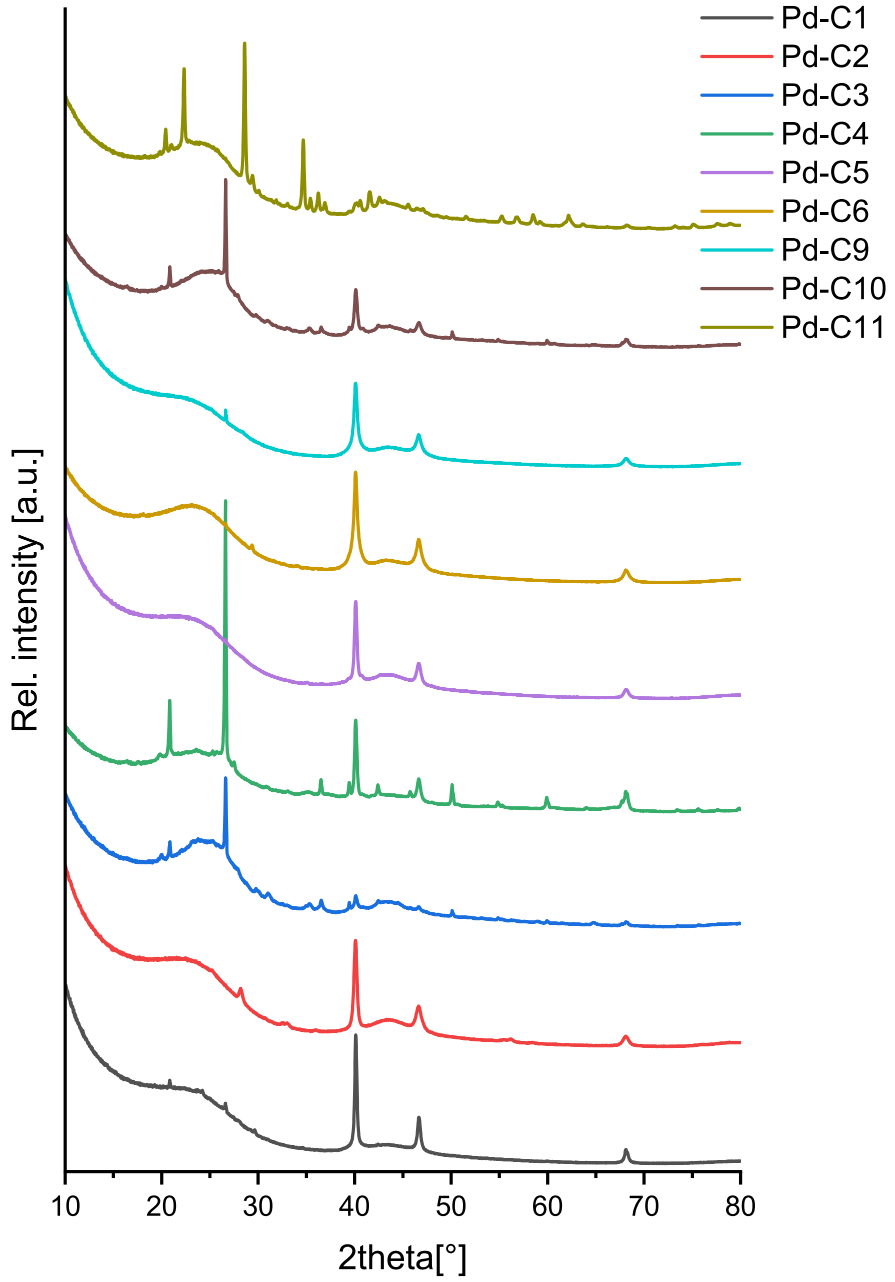

Figure S12. Plot of PXRD reflexes for palladium on carbon supports.

Plot of PXRD reflexes for Pd-C1, 3.33 wt.% Pd, AKROS C1: The Peaks correspond to cubic palladium (Joint Committee on Powder Diffraction Standards (JCPdS) card number 00-046-1043) and silicon oxide (JCPdS) card number 01-089-1961).

PXRD patterns of Pd-C2, 2.78 wt.% Pd, Support COC 4x8: The Peaks correspond to cubic palladium (JCPdS) card number 00-046-1043) and orthorhombic dicalcium silicate (JCPdS) card number 01-086-0399.

PXRD patterns of Pd-C3, 1.74 wt.% Pd, support AFA-4-1050-S: The Peaks correspond to cubic palladium (JCPdS) card number 00-046-1043), hexagonal silicon oxide (JCPdS card number 01-075-8322), tetragonal barium calcium iron oxide (JCPdS card number 01-079-7188) and monoclinic calcium aluminum iron(III) oxide (JCPdS card number 01-070-7252).

PXRD patterns of Pd-C4, 3.94 wt.% Pd, support COL 4x8: The Peaks correspond to cubic palladium (JCPdS card number 00-005-0681), silicon oxide (JCPdS card number 01-087-2096), rhombohedral iron(III) oxide (JCPdS card number 01-089-0596) and cubic iron oxide (JCPdS card number 01-078-6086).

PXRD patterns of Pd-C5 3.18 wt.% Pd, support EcoSorb CK1: The Peaks correspond to cubic palladium (JCPdS card number 03-065-2867), tetragonal sulfide (JCPdS card number 01-073-1387), hexagonal carbon (JCPdS card number 00-026-1077) and silicon oxide (JCPdS card number 01-089-1961).

PXRD patterns of Pd-C6 3.65 wt.% Pd, support WOS 4x8: The Peaks correspond to cubic palladium (JCPdS card number 00-046-1043) and rhombohedral calcium carbonate (JCPdS card number 01-080-9775).

PXRD patterns of Pd-C9 2.79 wt.% Pd, support EcoSorb CK4: The Peaks correspond to cubic palladium (JCPdS card number 03-065-2867) and rhombohedral carbon (JCPdS card number 01-075-2078).

PXRD patterns of Pd-C10 3.19 wt.% Pd, support COLPA 60: The Peaks correspond to cubic palladium (JCPdS card number 00-005-0681), hexagonal silicon oxide (JCPdS card number 01-075-8322), rhombohedral iron(III) oxide (JCPdS card number 01-071-5088), cubic iron titanium oxide (JCPdS card number 01-077-8401) and hexagonal barium iron titanium oxide (JCPdS card number 01-089-4609).

PXRD patterns of Pd-C11, 0.38 wt.% Pd, support COLPA 60: The Peaks correspond to cubic palladium (JCPdS card number 00-005-0681), hexagonal potassium aluminum silicate (JCPdS card number 01-078-3949), rhombohedral calcium carbonate (JCPdS card number 01-078-4614) and cubic triiron tetraoxide (JCPdS card number 01-089-0688).

PXRD Data of the Final Catalysts

PXRD of the fresh and used catalyst Pd-C1-OA-O_2 be seen below:

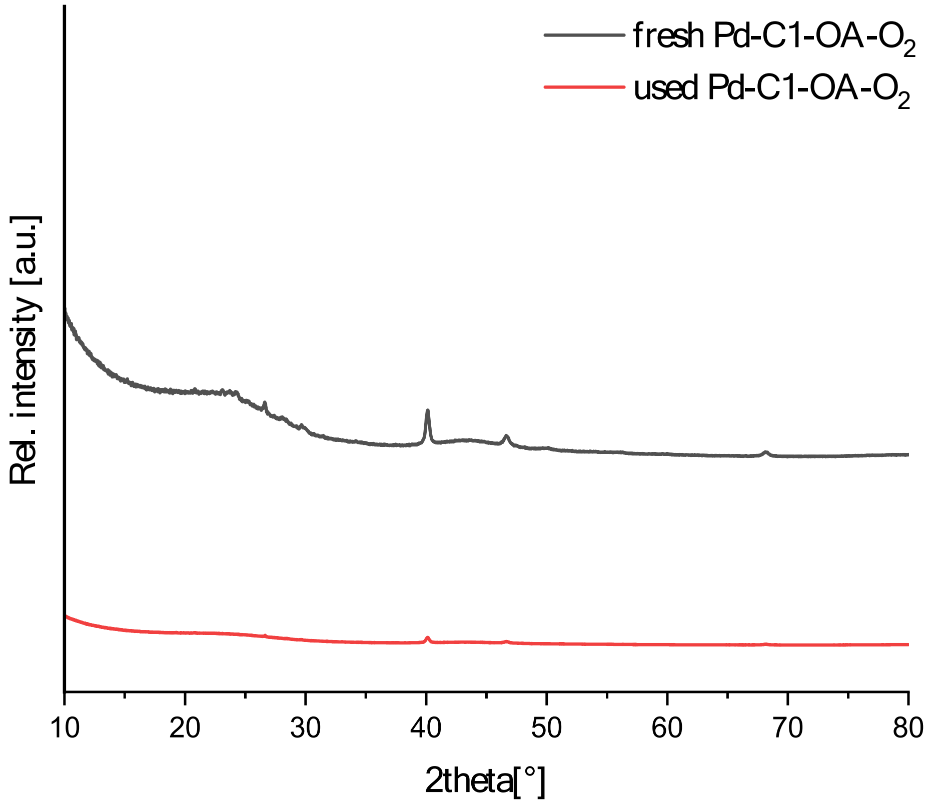

Figure S13. Plot of PXRD reflexes for fresh and used Pd-C1-OA-O2. The catalyst Pd-C1-OA-O_2 was used for FD for 10 h in a fixed-bed reactor.

PXRD patterns of fresh Pd-C1-OA-O2, 1.81 wt.% Pd, Support AKROS C1: The Peaks correspond to cubic palladium (JCPdS card number 00-046-1043), hexagonal silicon oxide (JCPdS card number 03-065-0466), monoclinic zirconium oxide (JCPdS card number 01-086-8527) and tetragonal zirconium cerium oxide (JCPdS card number 01-088-2397).

PXRD patterns of used Pd-C1-OA-O2, 1.81 wt.% Pd, Support AKROS C1: The Peaks correspond to cubic palladium (JCPdS card number 00-005-0681), hexagonal silicon oxide (JCPdS card number 01-087-2096;).

4.3 ICP-OES Data of the Catalysts Based on Different Carbon Supports

A detailed elementary analysis of the catalysts shown in Table 2 is shown below. The results are sorted in the same manner according to their activity in the (de)hydrogenation. All catalysts have been grinded with a ball mill prior to measurement and analyzed as powders.

Table S23. ICP-OES data for catalyst from the carbon pellet support screening.

**Catalyst name**	**Support**	**Pd [%]**	**P [%]**	**S [%]**	**Na [%]**	**K [%]**	**Ca [%]**	**Fe [%]**	**Ti [%]**	**Mn [%]**	**Sr [%]**
Pd-C1	AKROS C1	3.33	0.57	-	-	-	0.014	0.064	0.004	0.005	
Pd-C2	COC 4 x 8	2.78	-	0.046	-	-	-	0.012	-	-	-
Pd-C3	AFA-4-1050-S	1.75	-			-	0.481	0.900	0.068	0.011	0.018
Pd-C4	COL 4 x 8	3.94	0.037	0.319	0.684	0.394	0.059	0.189	0.161	0.024	0.009
Pd-C5	EcoSorb CK1	3.18	-	0.066				0.008		0.008	
Pd-C6	WOS 4 x 8	3.65	0.074	0.273	-	-	0.451	0.013	-	0.042	0.123
Pd-C7	EcoSorb CE55	2.70	-	-	0.055	-	-	0.123	-	0.003	-
Pd-C8	EcoSorb CE70	4.91	0.057		0.107			0.197	0.015	0.005	-
Pd-C9	EcoSorb CK4	2.79		0.076	-	-	0.017	0.009	-	0.013	-
Pd-C10	COLPA 60	3.19	0.074	0.273	0.900	-	0.421	0.989	0.081	0.018	0.032
Pd-C11	AFA-4-dot S	0.38	0.083		0.230	1.710		0.588	0.040	0.013	0.037
Pd-C12	Dopetac Sulfo 100	0.11	-			-	1.77	0.078	0.024	0.077	0.006

4.4 SEM Data for the Carbon Supports and Catalysts

SEM images have been used to determine the quality of the shell coating of catalyst. Following different carbon pellet supports and catalysts are shown. All data has been collected from the surface of an intact pellet catalysts. For the analysis of the cross-section, the pellet has been cut with a scalpel an analyzed accordingly.

SEM Data for the Carbon Supports

The carbon supports of the two best working catalysts from Table 2 haven been studied independently from the shell impregnated samples. The SEM images can be seen below:

<u>AKROS C1 (support for Pd-C1)</u>

Surface Cross-section

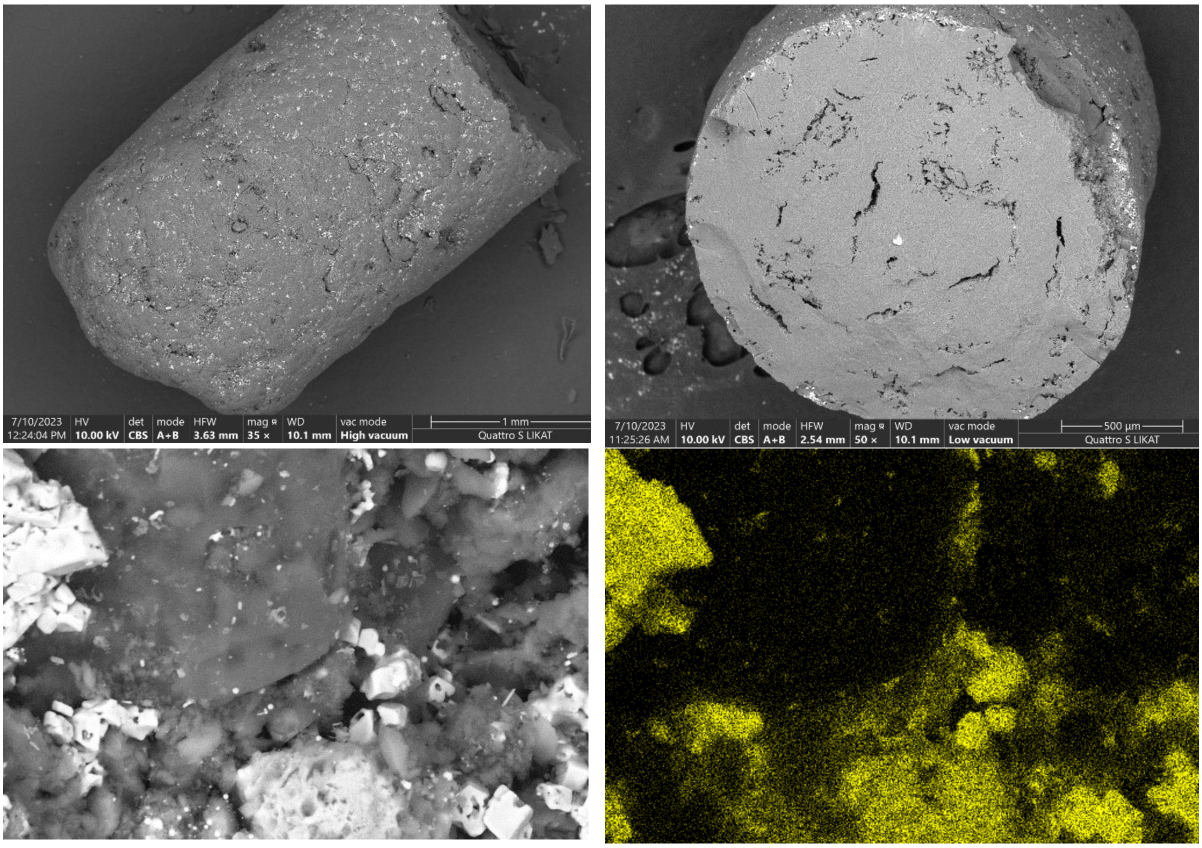

Figure S14. Backscattered electron images of the carbon pellet support AKROS C1 (top) and EDX scan of the cross-section (bottom).

EDS analysis shows the presence of phosphorus, which is in agreement with the elementary analysis.

COC 4x8 (Support for Pd-C2)

Surface Cross-section

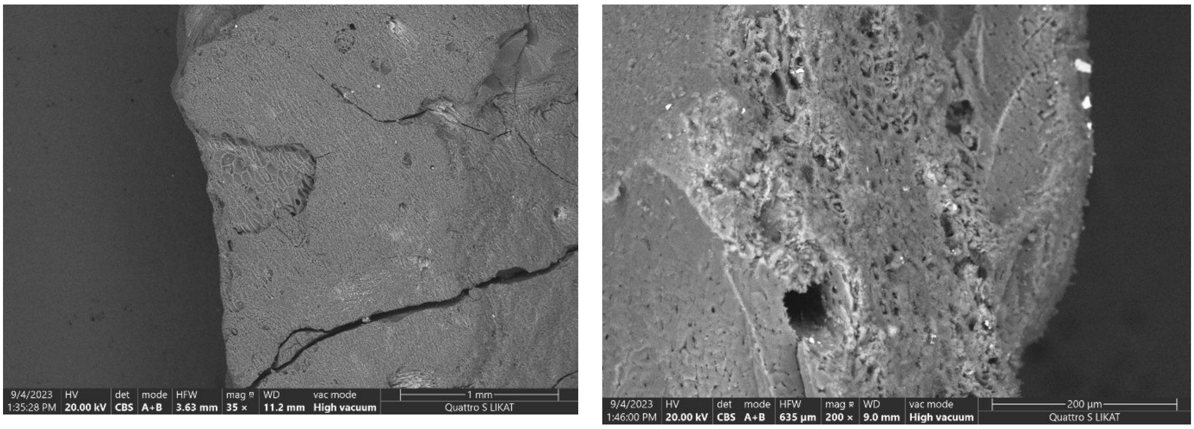

Figure S15. Backscattered electron images of the carbon support COC 4x8.

SEM Data for the Catalysts from the Carbon Support Screening

Following are the SEM images of the fresh catalysts shown in Table 2. They are sorted in the same manner according to their activity in the (de)hydrogenation.

Pd-C1 (3.33 wt.% Pd):

Surface Cross-section

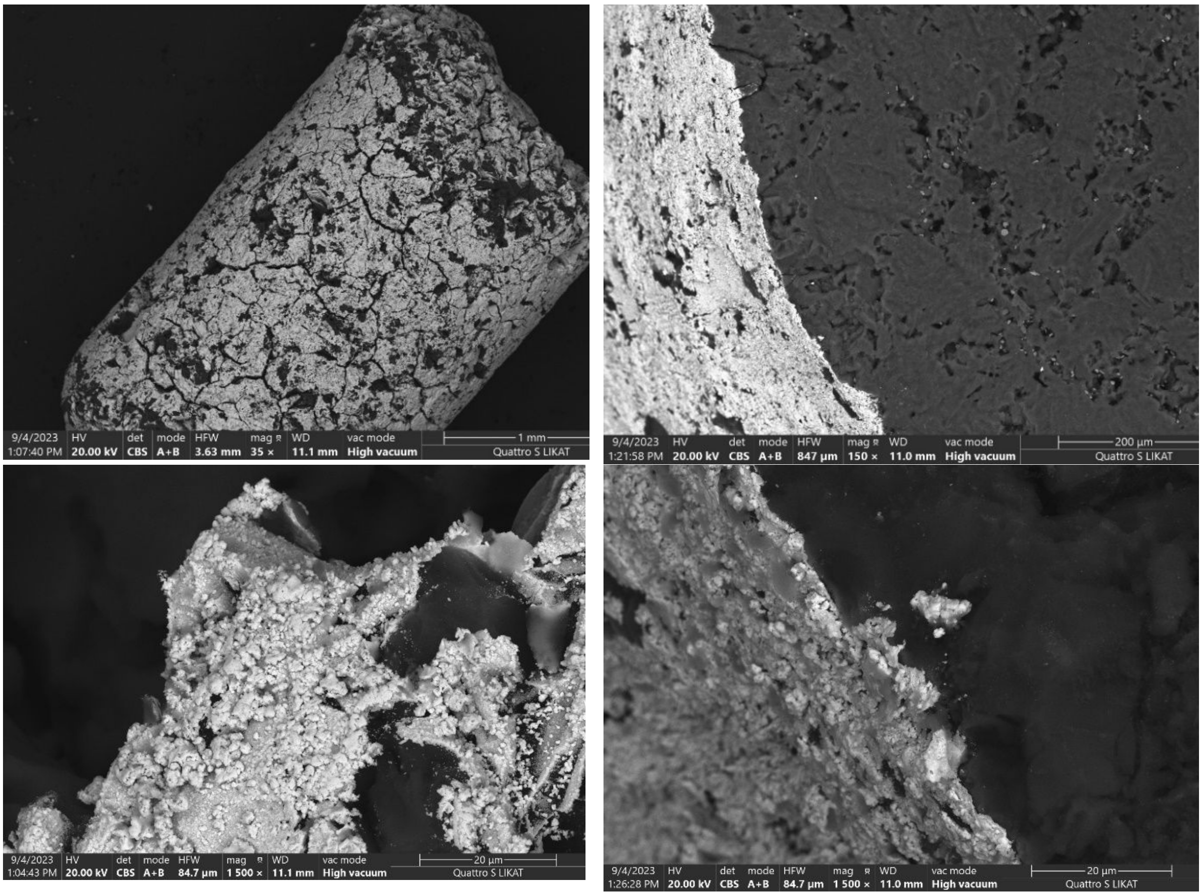

Figure S16. Backscattered electron images of the fresh catalyst Pd-C1. The surface (left) and the cross-section (right) are presented from a wider angel (top) and a close-up (bottom).

For further details see Figure 3.

Pd-C2 (2.78 wt.% Pd):

Surface Cross-section

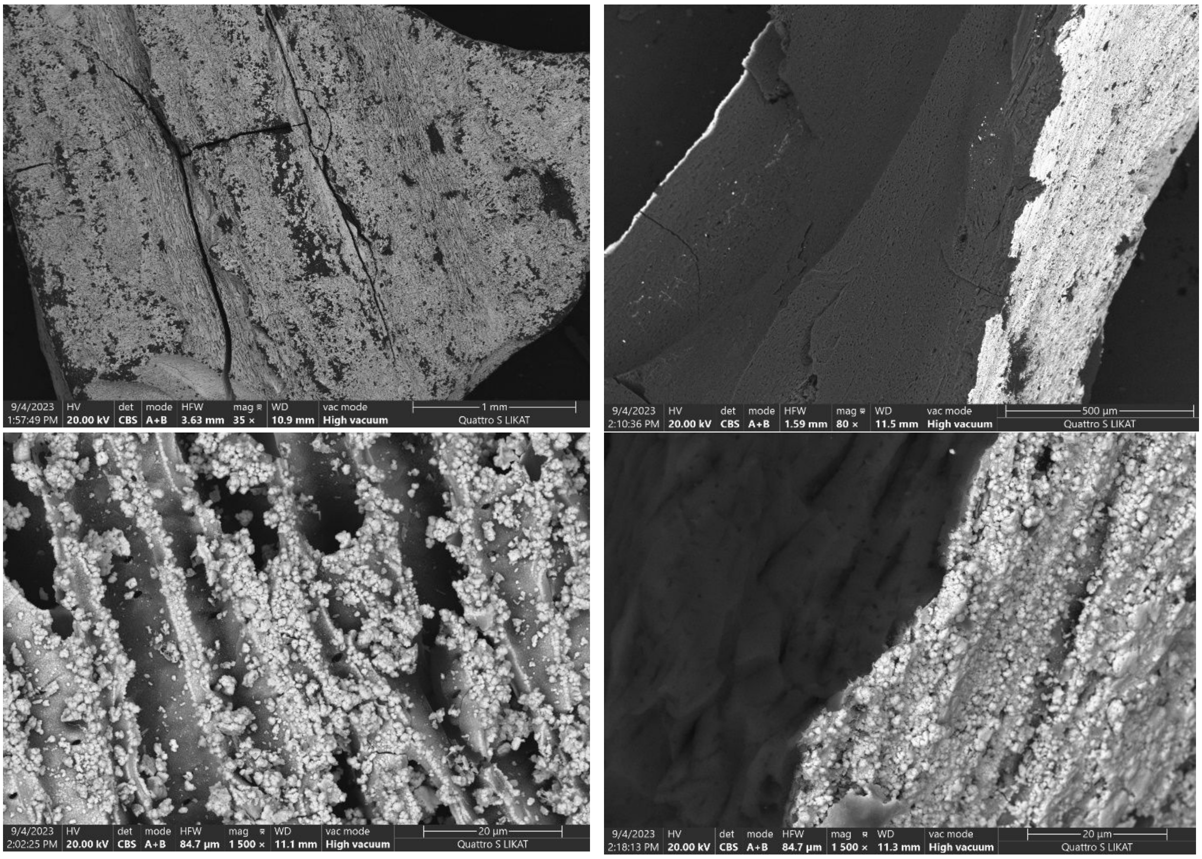

Figure S17. Backscattered electron images of the fresh catalyst Pd-C2. The surface (left) and the cross-section (right) are presented from a wider angel (top) and a close-up (bottom).

The close-up of the cross-section (right, bottom) shows an even thickness of the palladium crust on the surface of the pellet. For further details see Figure 1.

Pd-C3 (1.74 wt.% Pd):

Surface

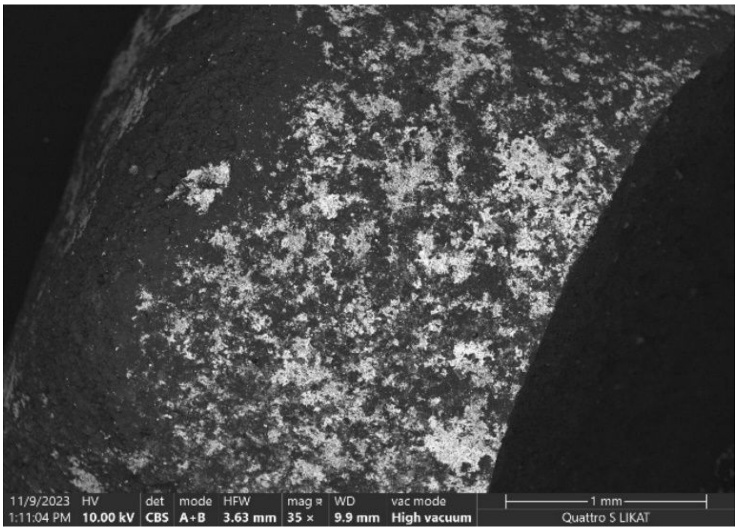

Figure S18. Backscattered electron image of the fresh catalyst Pd-C3.

Pd-C4 (3.94 wt.% Pd):

Surface

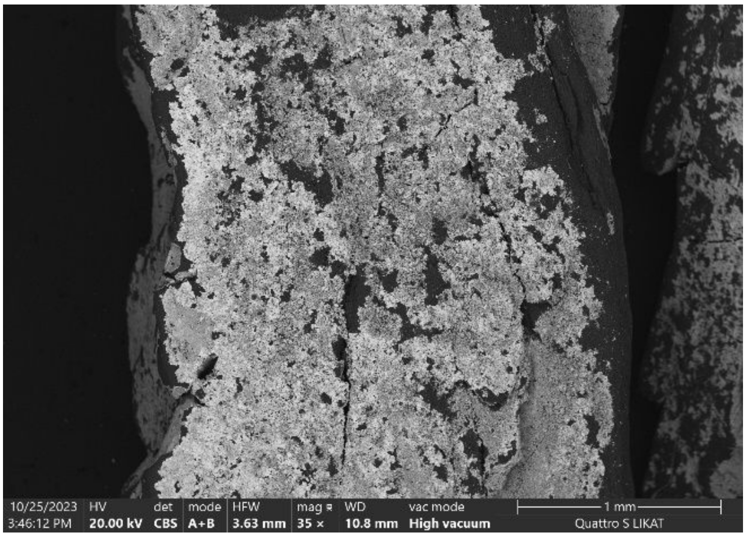

Figure S19. Backscattered electron images of the fresh catalyst Pd-C4.

Pd-C5 (3.18 wt.% Pd):

Surface Cross-section

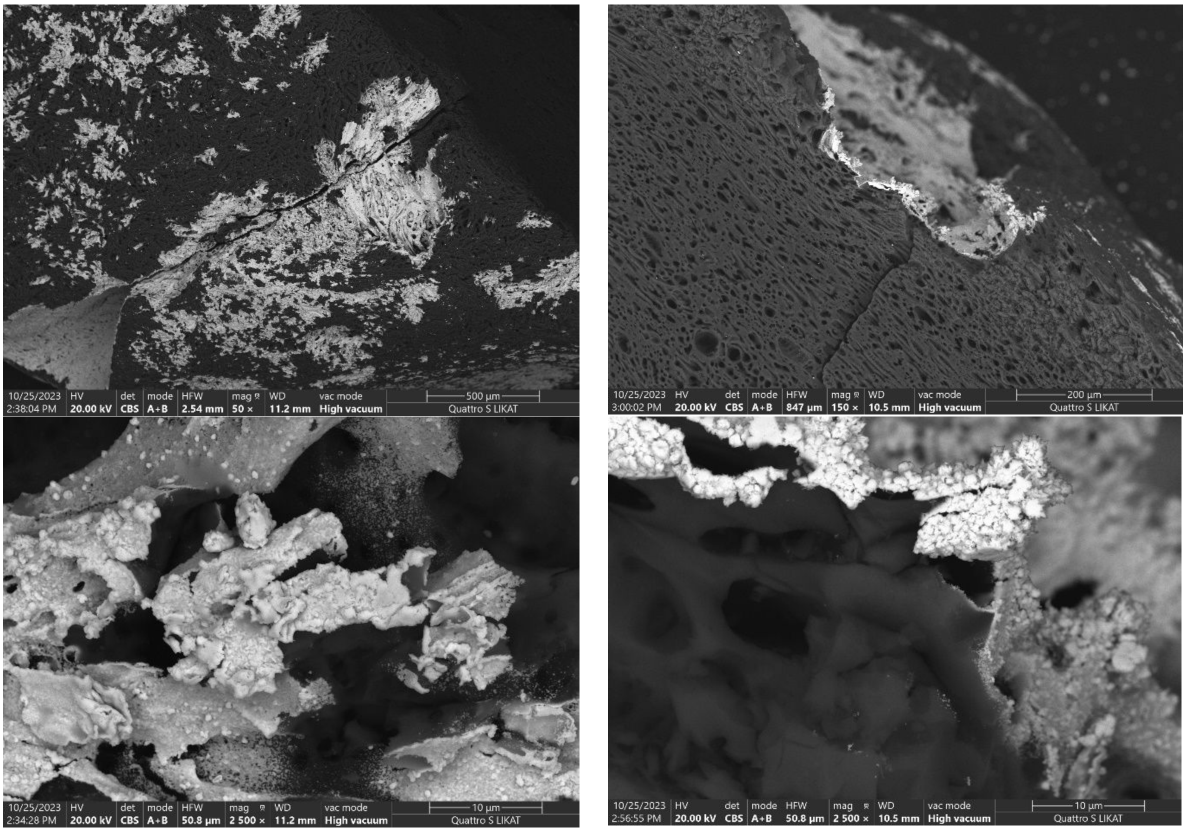

Figure S20. Backscattered electron images of the fresh catalyst Pd-C5. The surface (left) and the cross-section (right) are presented from a wider angel (top) and a close-up (bottom).

The cross-section (right, top) shows parts of the surface with a metal crust and parts without. The close-up of the cross-section (right, bottom) emphasized the thickness of the palladium layer on the spots where metal was deposited on the surface of the pellet. For further details see Figure 1.

Pd-C6 (3.65 wt.% Pd):

Surface

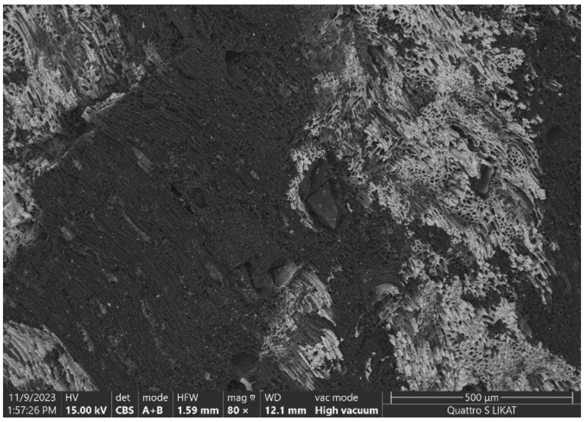

Cross-section

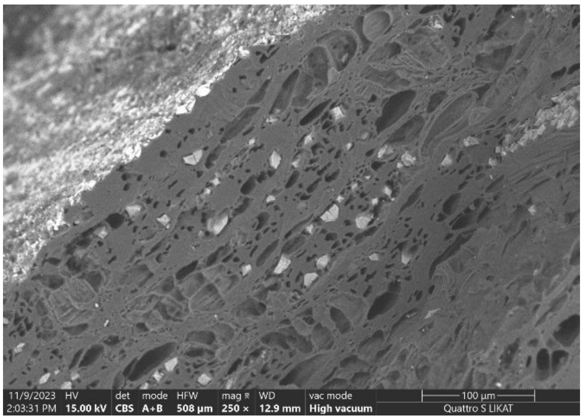

EDX analysis of Pd

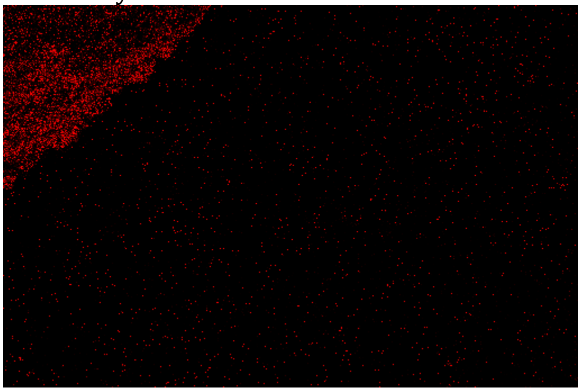

EDX analysis of Ca

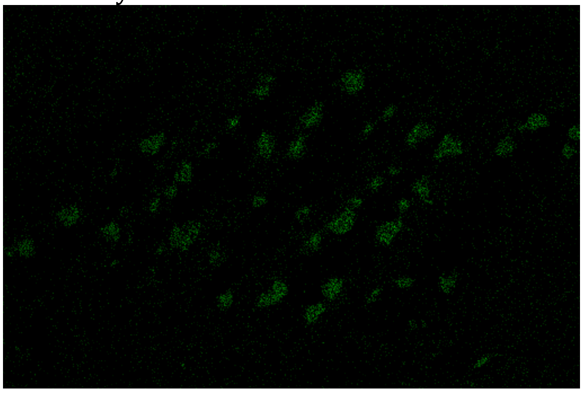

Figure S21. Backscattered electron images of the fresh catalyst Pd-C6 (top) and the corresponding EDX analysis of the cross-section (bottom). Pd is shown in red and Ca in green.

EDX analysis identifies the particles in the cross section as calcium species which is in agreement with the elementary analysis (chapter 4.1, Table S20).

Pd-C9 (2.76 wt.% Pd):

Surface

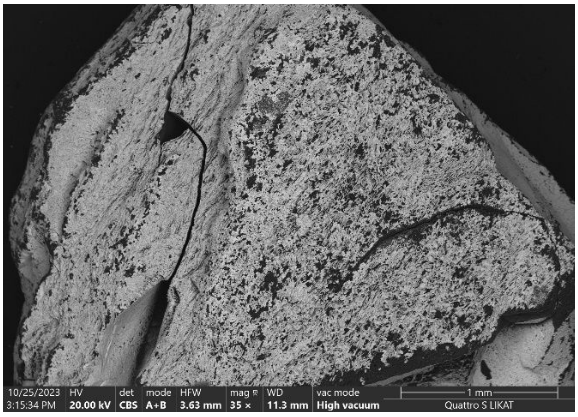

Cross-section

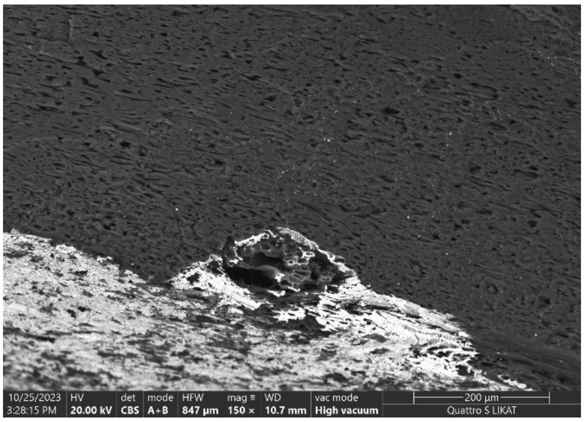

Figure S22. Backscattered electron images of the fresh catalyst Pd-C9.

Pd-C10 (3.19 wt.% Pd):

Surface

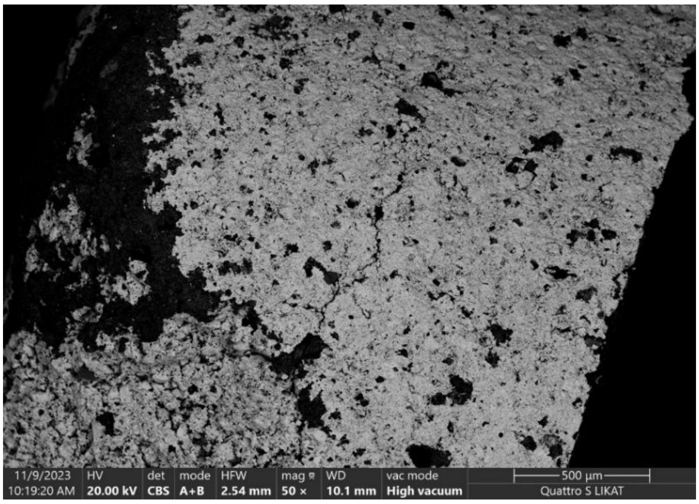

Figure S23. Backscattered electron images of the fresh catalyst Pd-C10.

Pd-C11 (0.38 wt.% Pd):

Surface

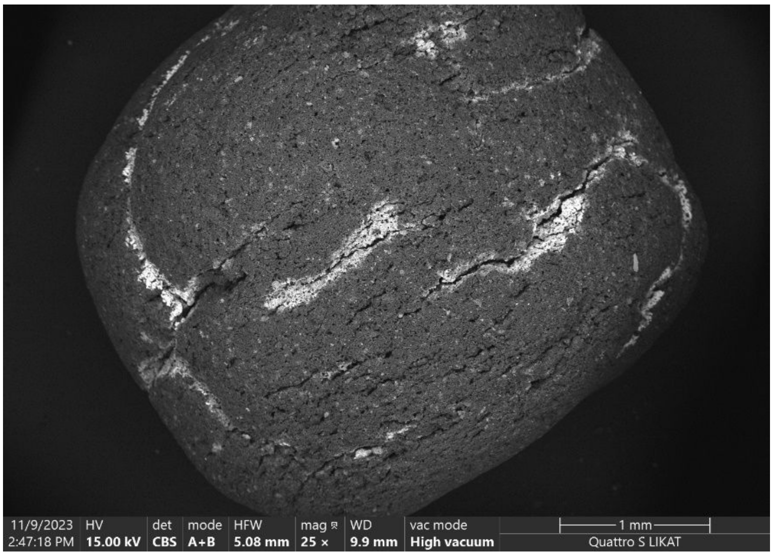

Figure S24. Backscattered electron images of the fresh catalyst Pd-C11.

Particle Size Analysis for Different Pd Loadings Based on SEM

The addition of oxalic acid in the impregnation step made an even metal coating of the pellet support possible even at low palladium loadings. The effect is shown in Table 4. and the corresponding Plot G. We utilized SEM images of the catalysts at different palladium loading to estimate the crust thickness (from the cross-section of the pellet catalysts) as well as the particle size (from the surface of the pellet catalysts) on a macroscopic scale. An overview over the analyzed spots is given in Table S24, the corresponding SEM images are shown in Figure S27-S31. An analysis of a possible correlation between the metal loading and the activity is given in Figure S32.

Table S24. Palladium on carbon catalysts with different palladium loadings. Summary of the analyzed crust and particle sizes for different metal loadings analyzed with SEM.

**Entry**	**Name**	**Pd_{theo} [wt.%]**	**Pd_{meas} [wt.%]**	**Shell Thickness [nm]**
1	3.90Pd-C1-OA	5.00	3.90	4002-4868
2	3.19Pd-C1-OA	4.00	3.19	781-799
3	2.62Pd-C1-OA	3.00	2.62	513-626
4	1.60Pd-C1-OA	2.00	1.60	461
5	0.82Pd-C1-OA	1.00	0.82	n.d.

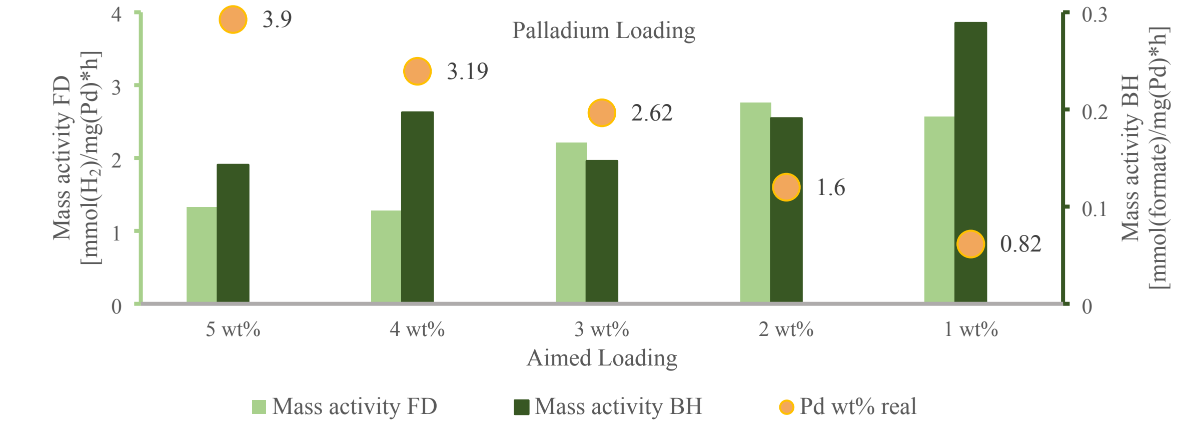

Catalyst synthesis: Na_2PdCl_4 (10 mL H_2O) was added dropwise to the carbon pellet AKROS C1 (808 mg, 50 mL H_2O) at 60 °C. The solution was stirred until it cleared up. The solvent was evaporated. The catalyst was washed, dried and reduced with H_2 (2 h, 200 °C). Theoretical metal content (Pd_{theo}) and measured content (Pd_{meas}) are listed. Catalytic screening: BH: 5.4 mmol $KHCO_3$, 20 mg Pd/C, 1.5 mL H_2O, 30 bar H_2, 60 °C, 18 h; FD: 250 mmol $KHCO_2$, 7.5 mmol K_2CO_3, 106 mg catalyst, 25 mL H_2O, 60 °C, 3 h.

Following, selected backscattered electron images of the cross-section (for thickness of the crust) and surface (for particles size analysis) are shown for the five catalysts from Table S24:

3.90Pd-C1-OA (5.00 wt.% Pd_{theo}; 3.90 wt.% Pd_{meas}):

Cross-section:

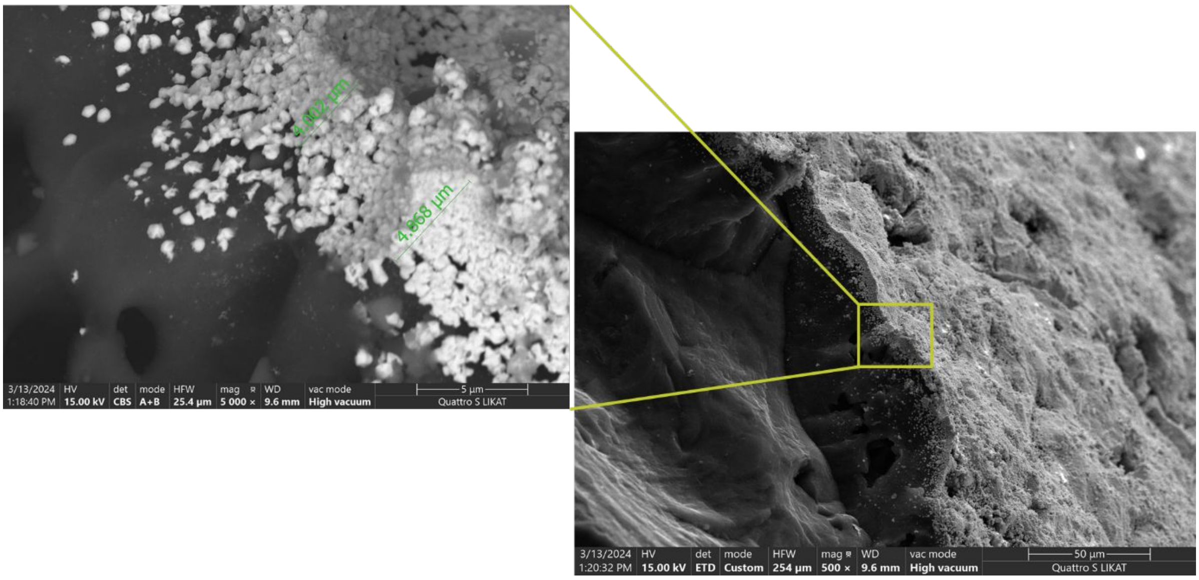

Surface:

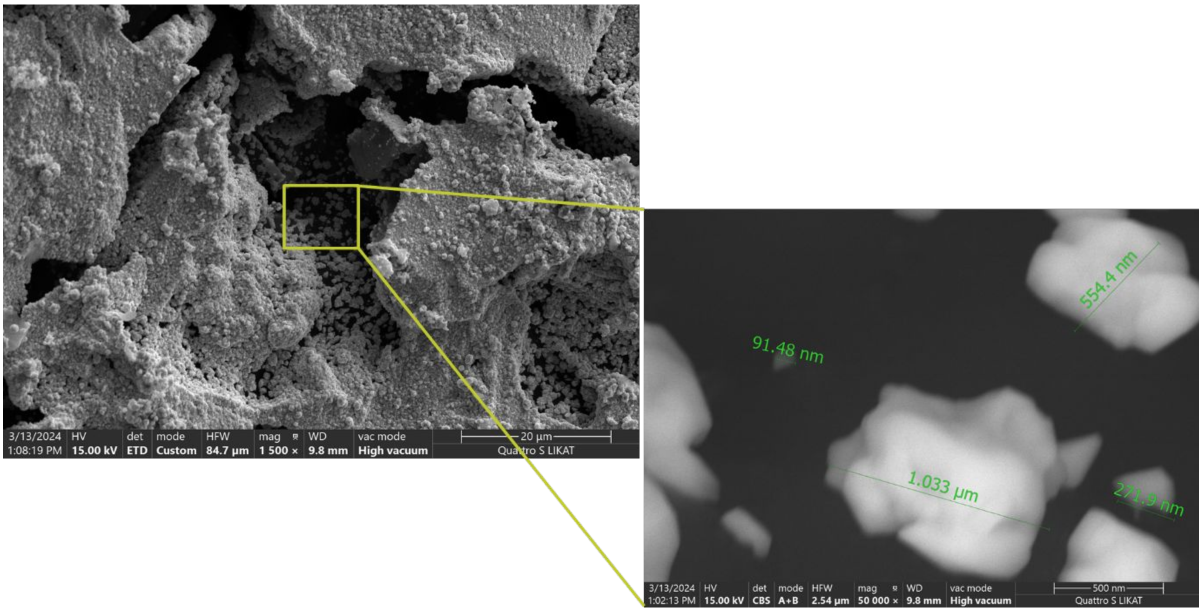

Figure S25. Backscattered electron images of the fresh catalyst 3.90Pd-C1-OA.

3.19Pd-C1-OA (4.00 wt.% Pd_{theo}; 3.19 wt.% Pd_{meas}):

Cross-section:

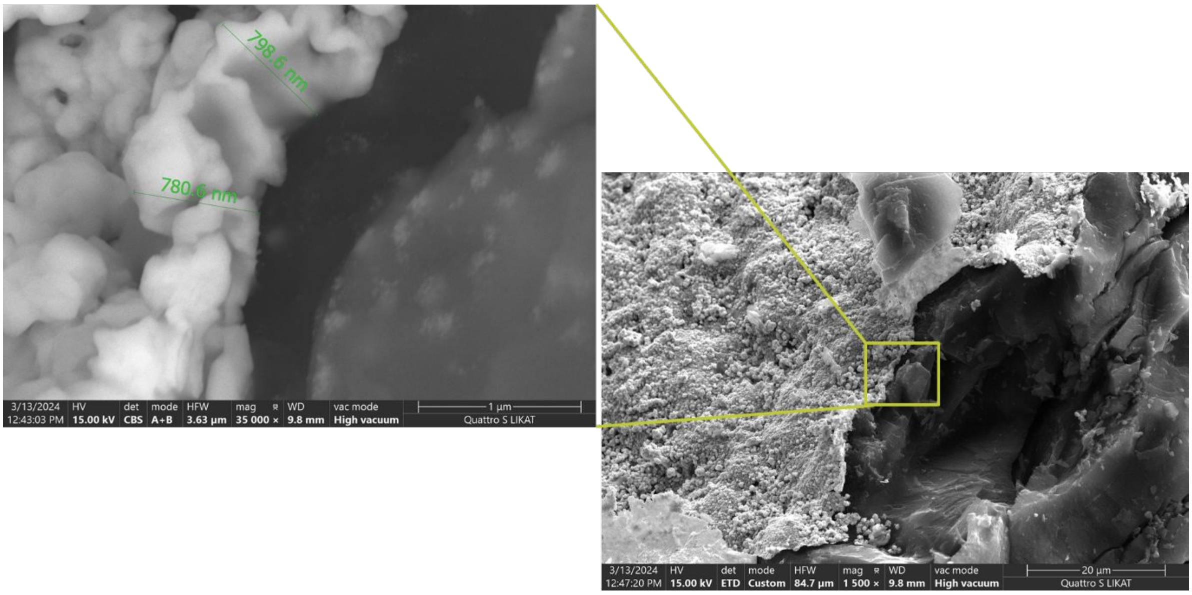

Surface:

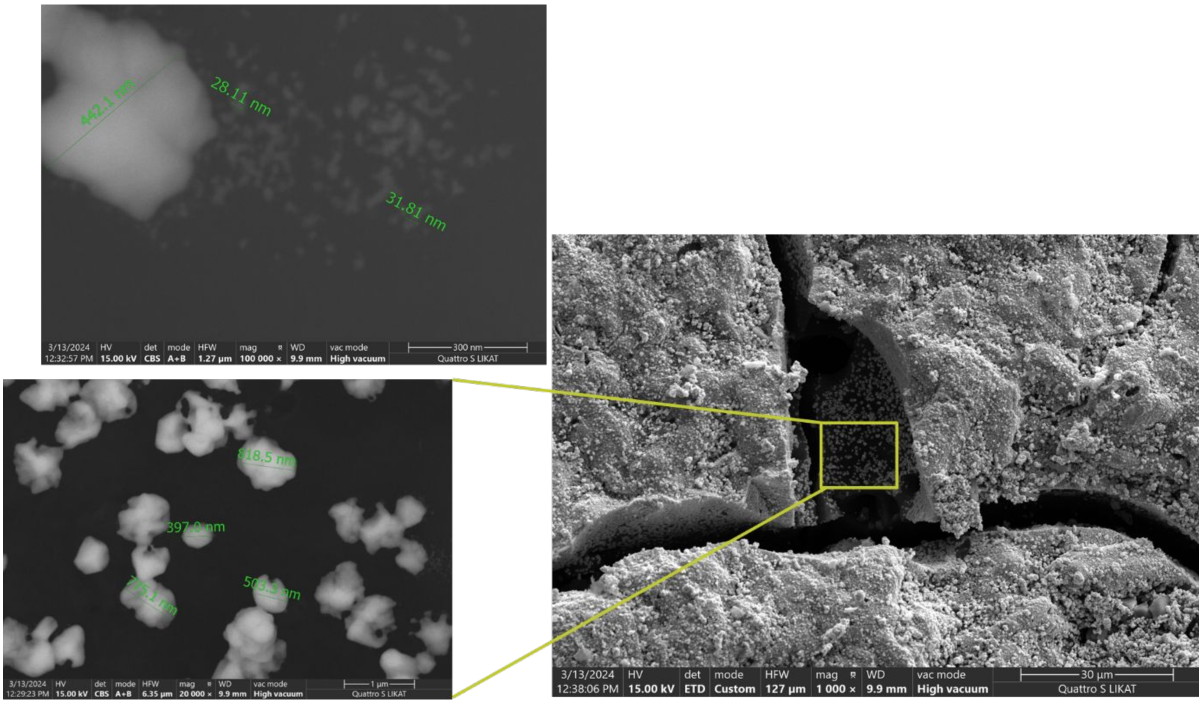

Figure S26. Backscattered electron images of the fresh catalyst 3.19Pd-C1-OA.

2.62Pd-C1-OA (3.00 wt.% Pd_{theo}; 2.62 wt.% Pdmeas):

Cross-section:

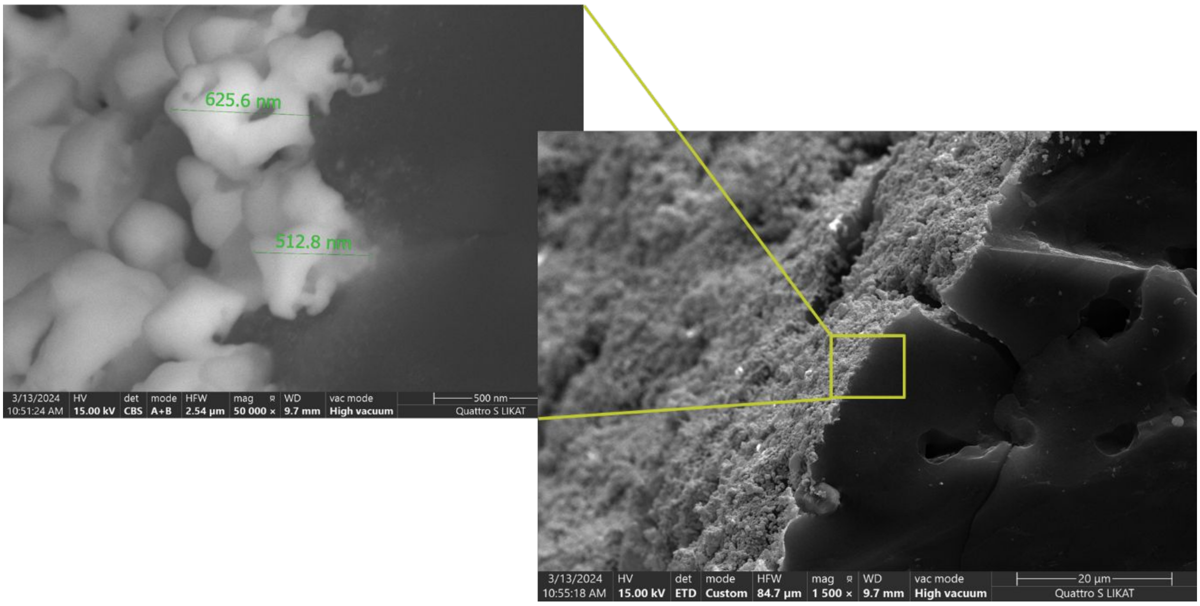

Surface:

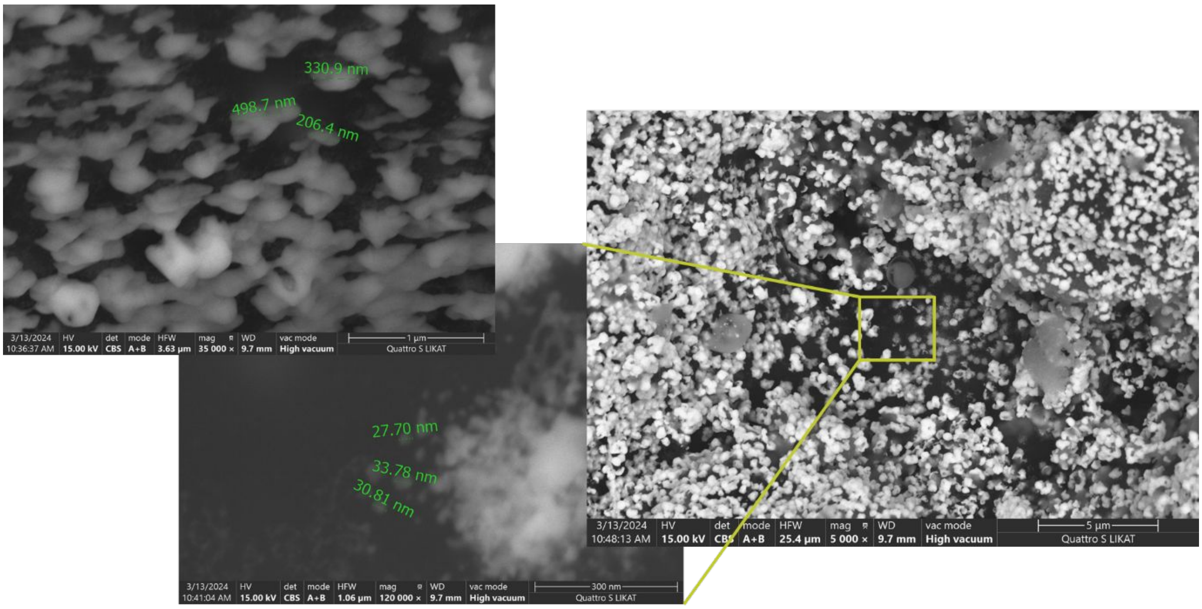

Figure S27. Backscattered electron images of the fresh catalyst 2.62Pd-C1-OA.

1.60Pd-C1-OA (2.00 wt.% Pd_{theo}; 1.60 wt.% Pd_{meas}):

Cross-section:

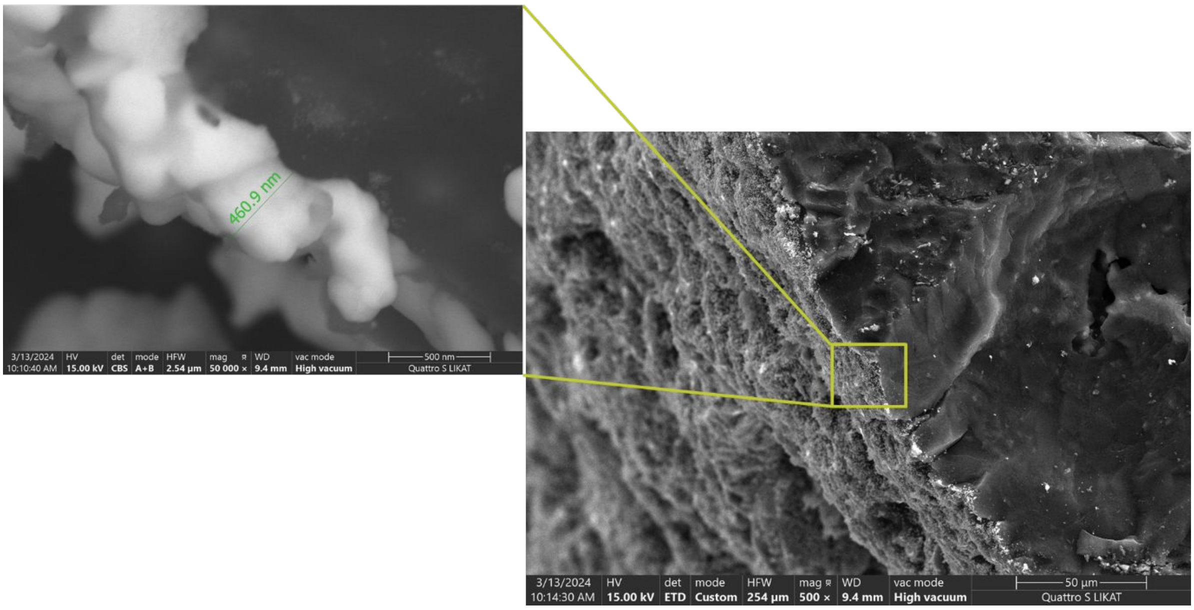

Surface:

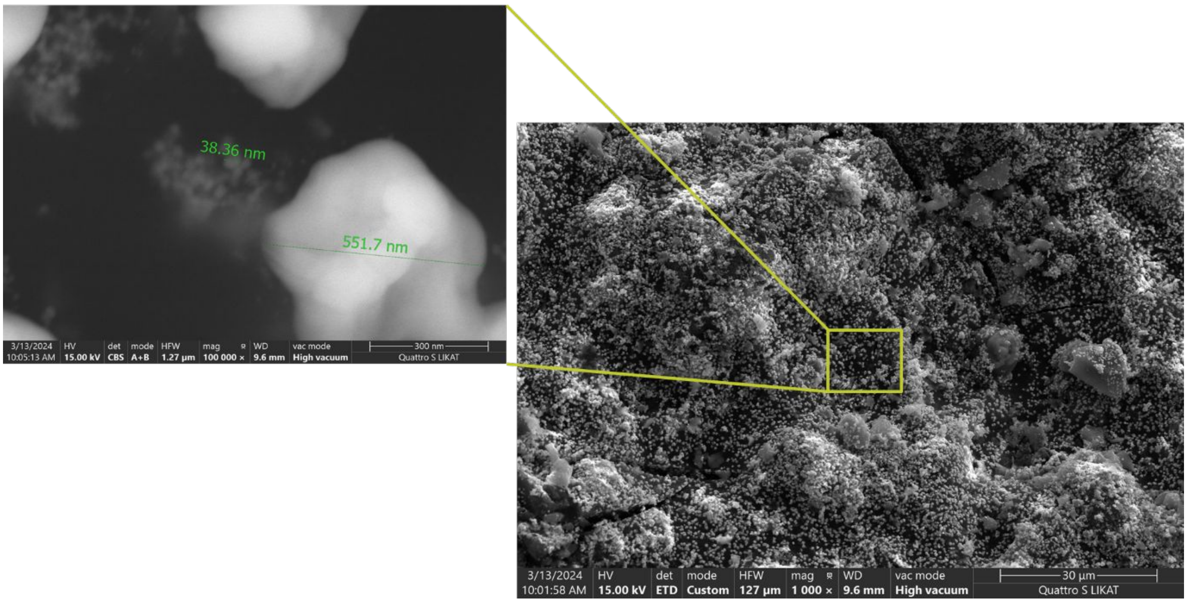

Surface

EDX analysis of Pd

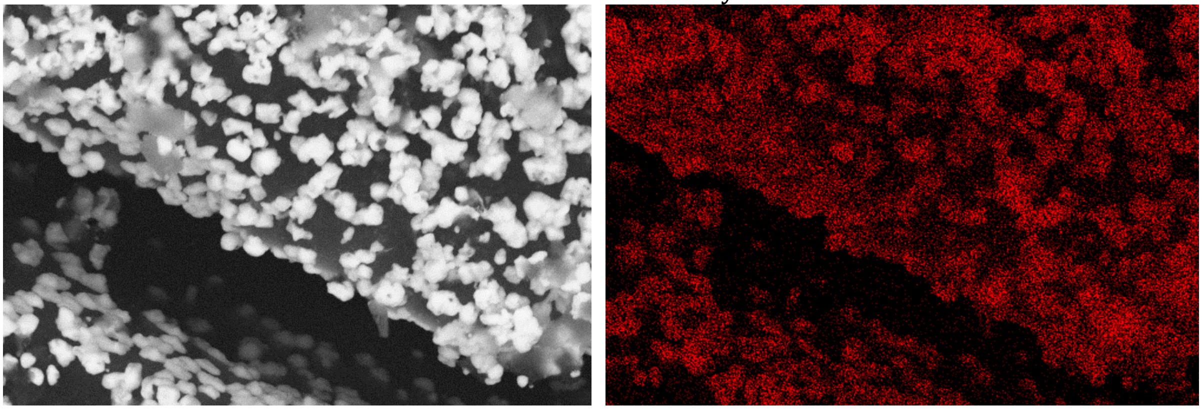

Figure S28. Backscattered electron images of the fresh catalyst 1.60Pd-C1-OA and the corresponding EDX analysis of the surface (bottom). The refenrece is shown in grey and Pd in red.

0.82Pd-C1-OA (1.00 wt.% Pd_{theo}; 0.82 wt.% Pd_{meas}):

Cross-section:

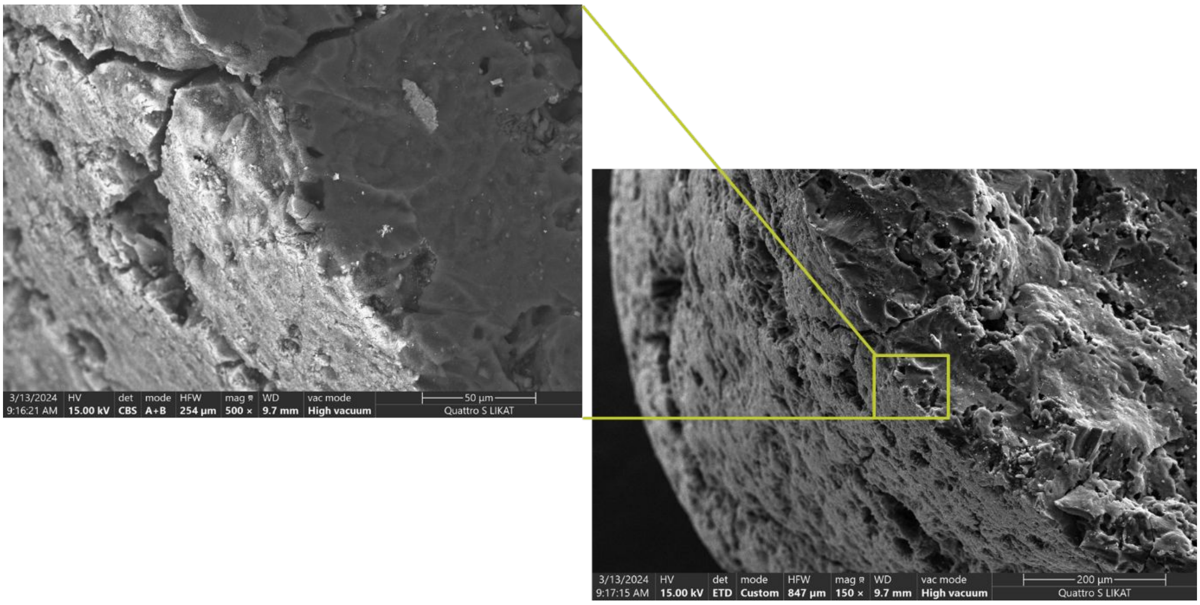

Surface:

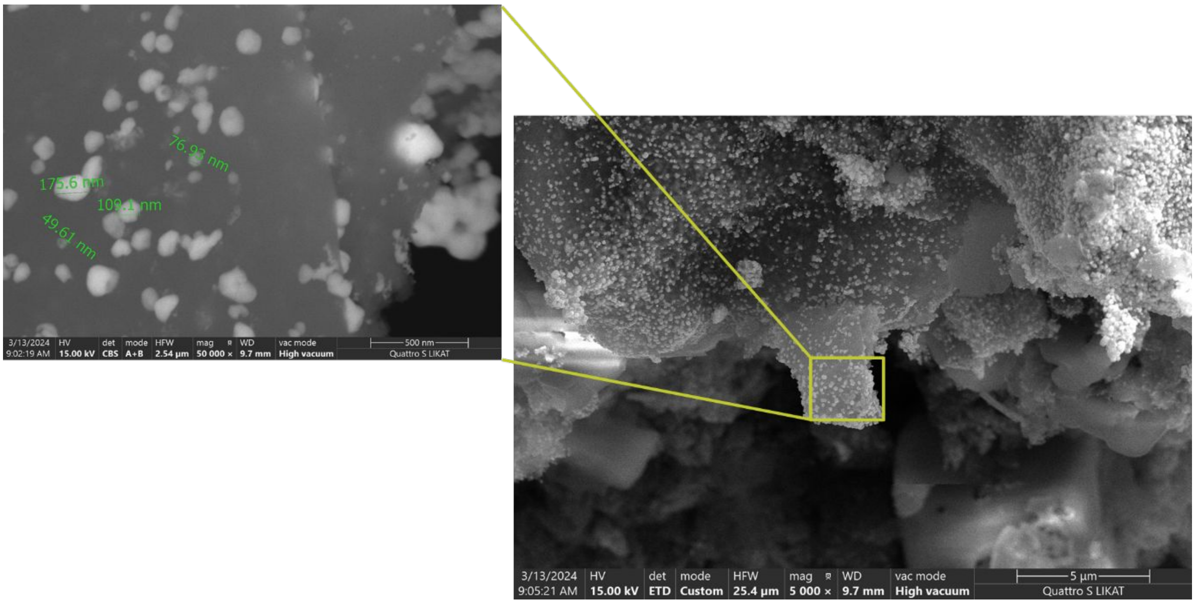

Figure S29. Backscattered electron images of the fresh catalyst 0.82Pd-C1-OA.

There is no clear correlation between metal loading and activity of the catalyst.

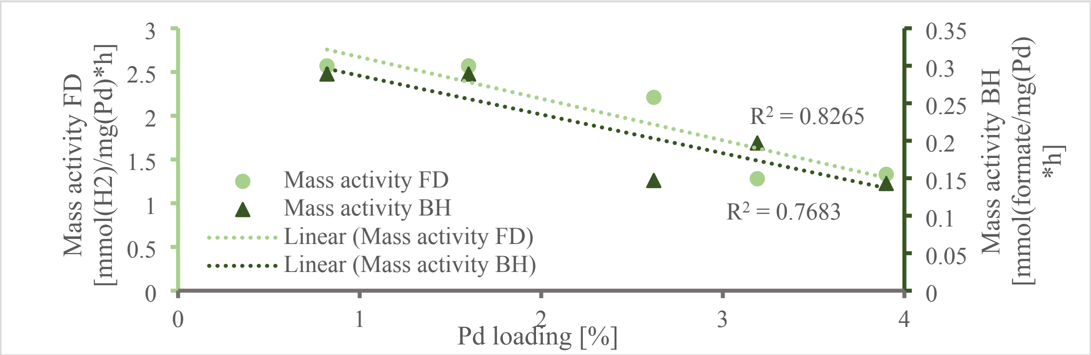

Figure S30. Metal loading vs activity of the catalysts.

SEM Analysis of the Final Catalyst Pd-C1-OA-O2

The final catalyst has been analyzed with SEM. The fresh sample is shown below:

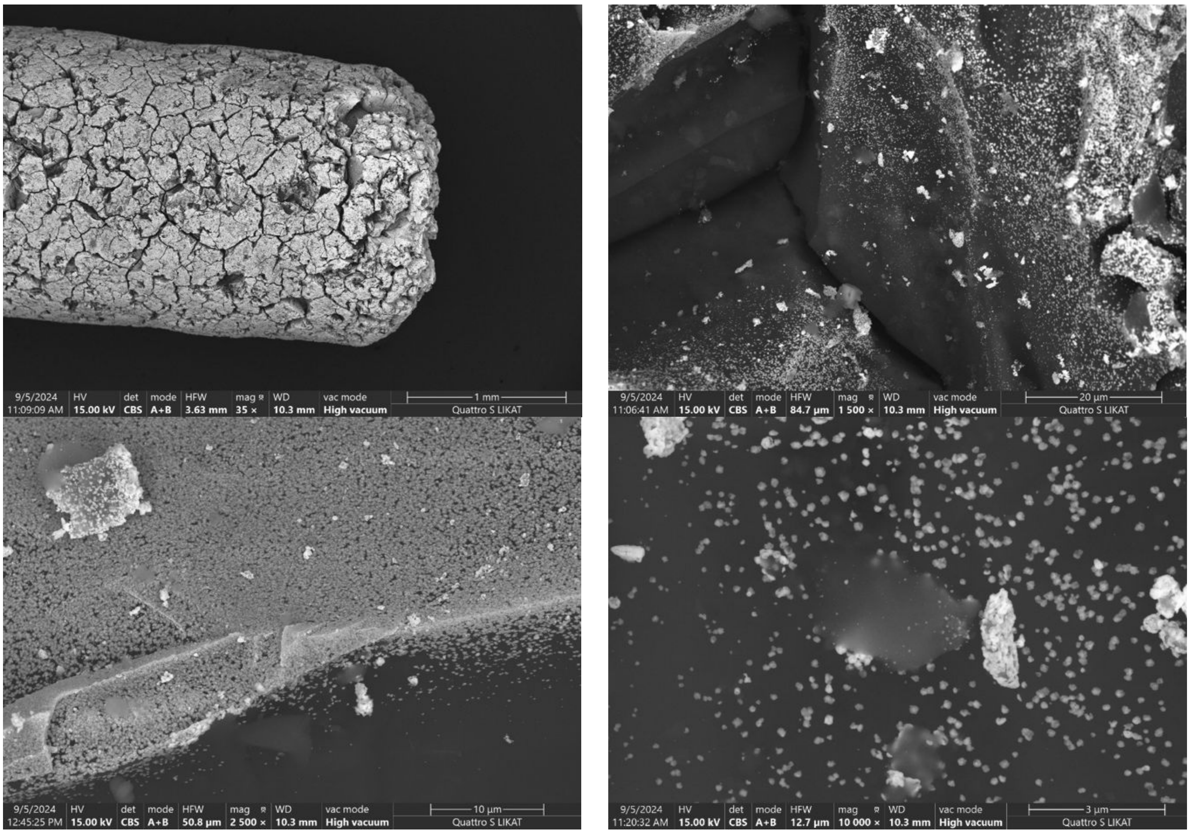

Figure S31. Backscattered electron images for the fresh catalyst Pd-C1-OA-O_2.

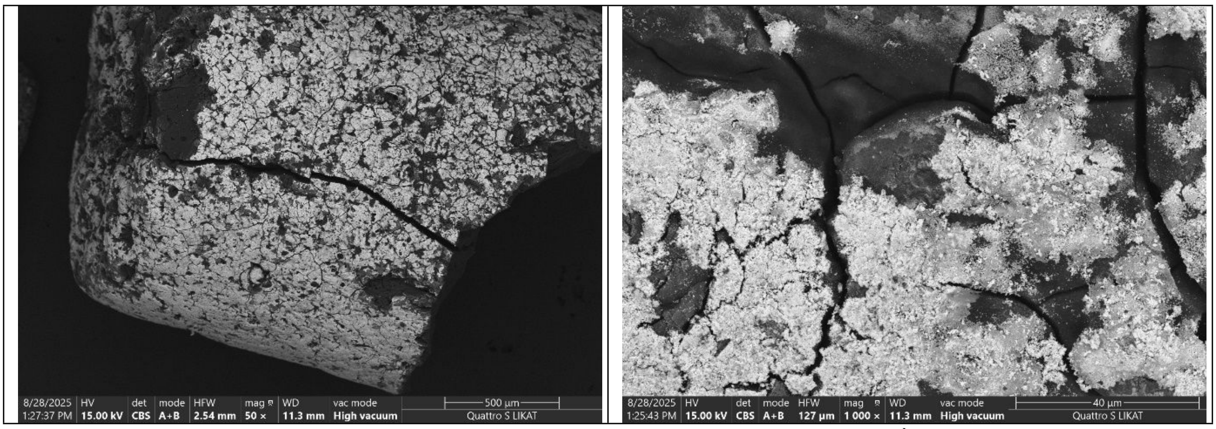

Figure S32. Backscattered electron images for the used catalyst Pd-C1-OA-O_2 after the 9th run FD.

4.5 BET Data for Carbon Supports and Catalysts

BET data for selected pellet carbon supports and their corresponding catalysts shown in Table 2. The data is sorted according to the activity of the catalysts in the (de)hydrogenation. The data was collected from the catalyst in the form of pellets.

Table S25. BET data for catalysts and carbon support.

**Entry**	**Catalyst name / carbon support**	**Single point surface area at P/Po [m²/g]**	**BET Surface Area [m²/g]**	**t-Plot Micropore Area [m²/g]**	**t-Plot External Surface Area [m²/g]**	**t-Plot micropore volume [m³/g]**
1	AKROS C1	1147	1191	940	251	0.58
2	Pd-C1	1120	1166	908	258	0.56
3	Pd-C2	1031	1050	1022	28	0.45
4	Pd-C3	825	856	831	24	0.47
5	Pd-C4	717	730	708	22	0.35
6	Pd-C5	1025	1035	1016	19	0.44
7	Pd-C9	1155	1183	1157	26	0.52
8	Pd-C11	797	812	786	26	0.35
9	Pd-C10	732	753	716	37	0.43

4.6 XPS Data for the Catalysts

XPS analysis has been performed at multiple stages of the optimization of the synthesis. The data was collected from the catalyst in the form of pellets

XPS Data for Catalysts from the Carbon Support Screening

The chemical state of the palladium metal has been analyzed for selected catalysts from the carbon support screening presented in Table 2. The results are shown below:

<u>XPS plot of Pd-C1:</u>

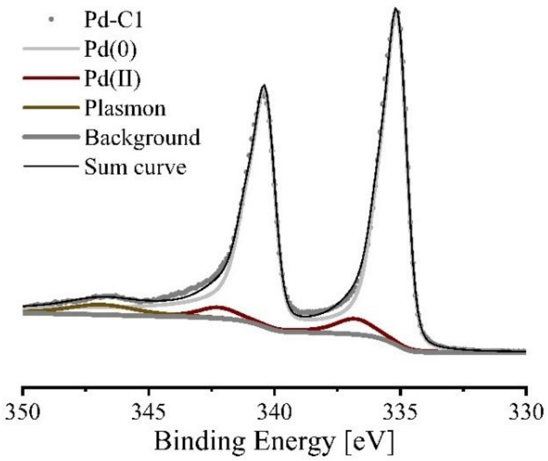

Figure S33. XPS plot of the Pd3d region.

<u>XPS plot of Pd-C2:</u>

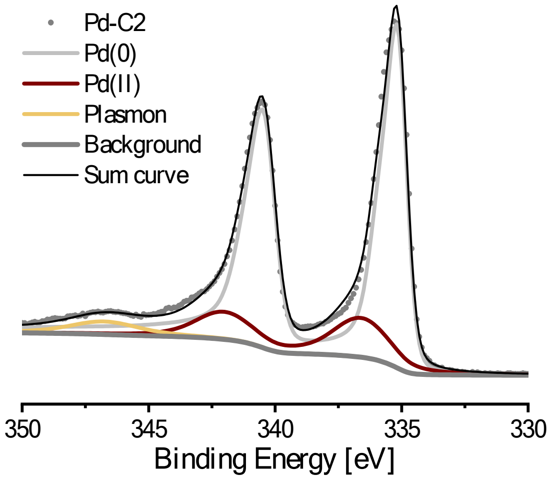

Figure S34. XPS plot of the Pd3d region.

XPS plot of Pd-C3:

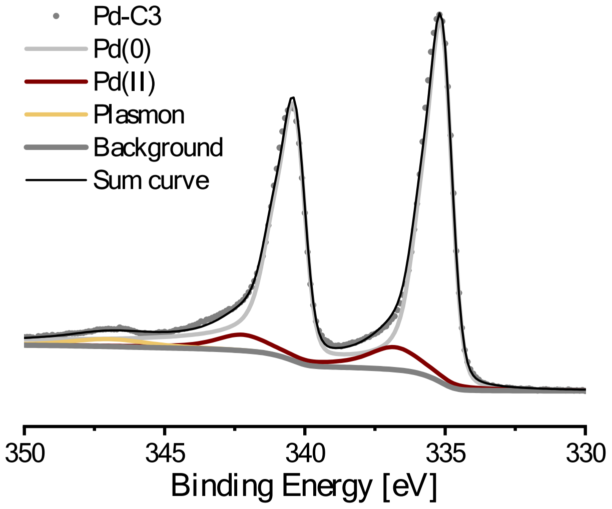

Figure S35. XPS plot of the Pd3d region.

XPS plot of Pd-C4:

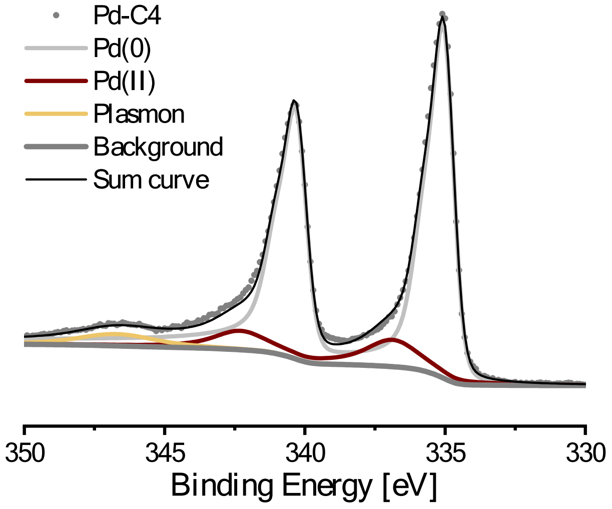

Figure S36. XPS plot of the Pd3d region.

XPS plot of Pd-C5:

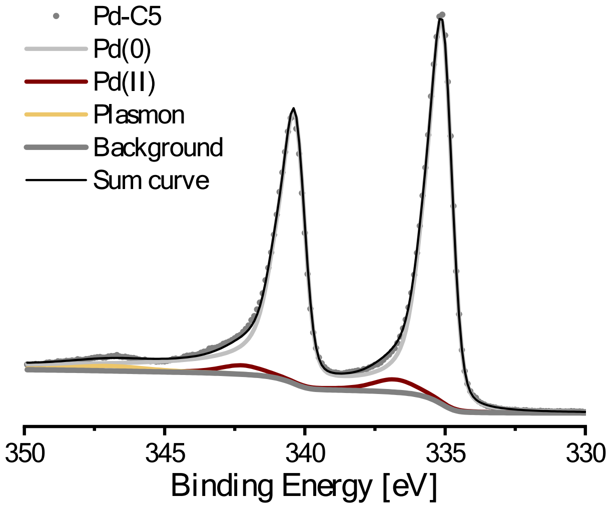

Figure S37. XPS plot of the Pd3d region.

XPS plot of Pd-C6:

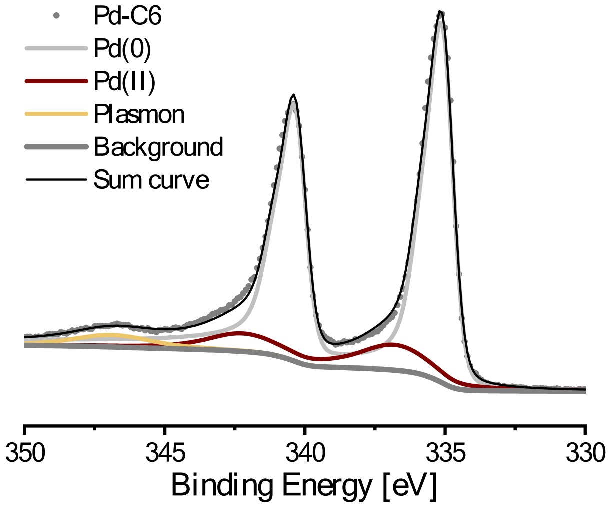

Figure S38. XPS plot of the Pd3d region.

XPS plot of Pd-C9:

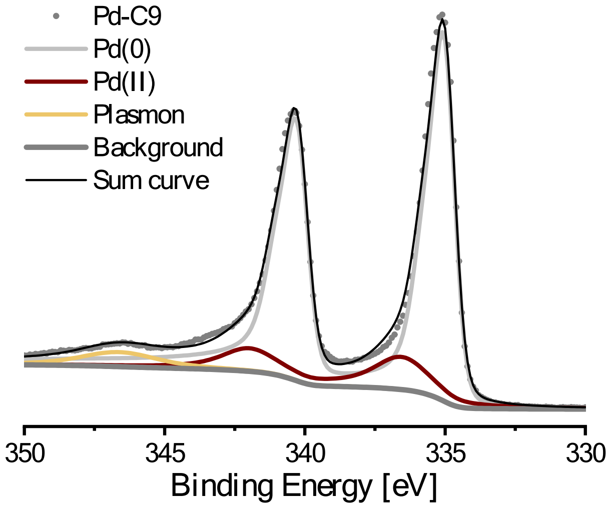

Figure S39. XPS plot of the Pd3d region.

XPS plot of Pd-C10:

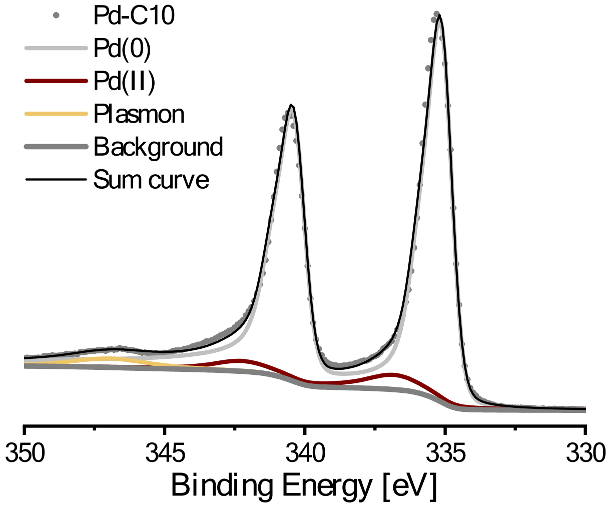

Figure S40. XPS plot of the Pd3d region.

XPS plot of Pd-C11:

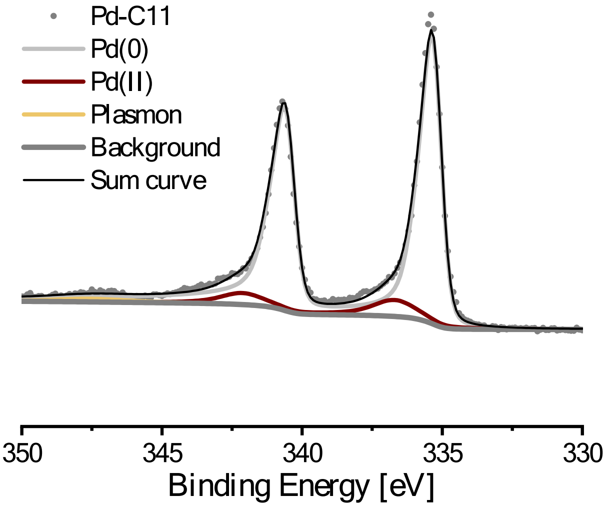

Figure S41. XPS plot of the Pd3d region.

XPS Data for Catalysts from the Metal Loading Variation

The theoretical metal loading was varied in between 5-1 wt%. The XPS analysis of the resulting catalyst is shown below:

XPS plot of 3.90Pd-C1-OA:

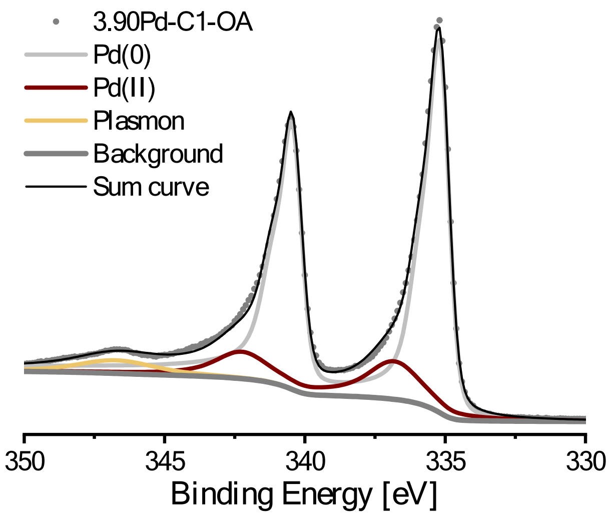

Figure S42. XPS plot of the Pd3d region.

XPS plot of 3.19Pd-C1-OA:

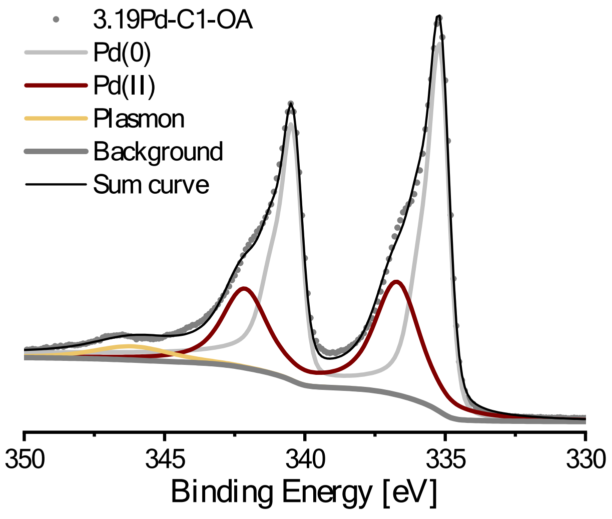

Figure S43. XPS plot of the Pd3d region.

XPS plot of 2.62Pd-C1-OA:

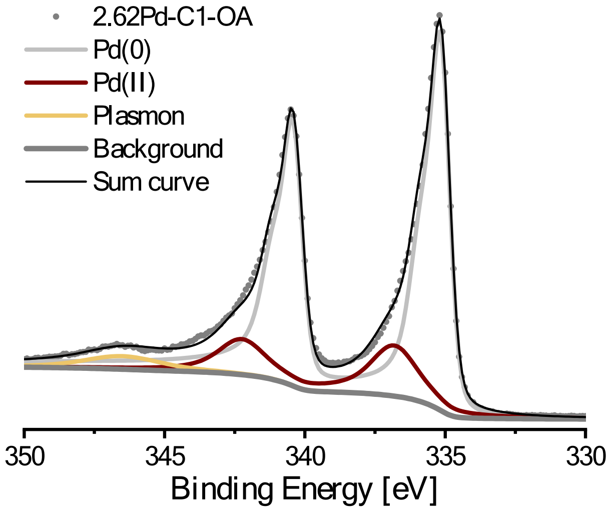

Figure S44. XPS plot of the Pd3d region.

XPS plot of 1.60Pd-C1-OA:

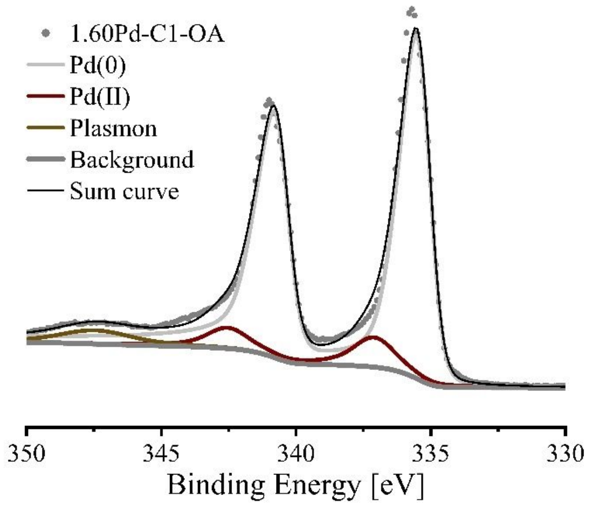

Figure S45. XPS plot of the Pd3d region.

XPS plot of 0.82Pd-C1-OA:

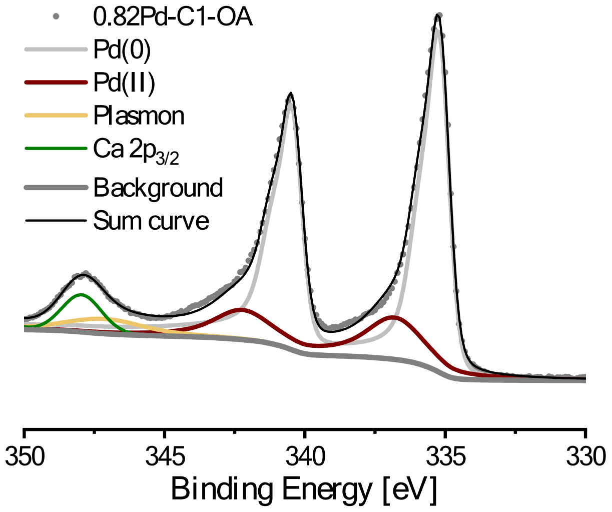

Figure S46. XPS plot of the Pd3d region.

XPS Data for the Final Catalysts Pd-C1-OA-O_2

XPS analysis of the fresh catalyst:

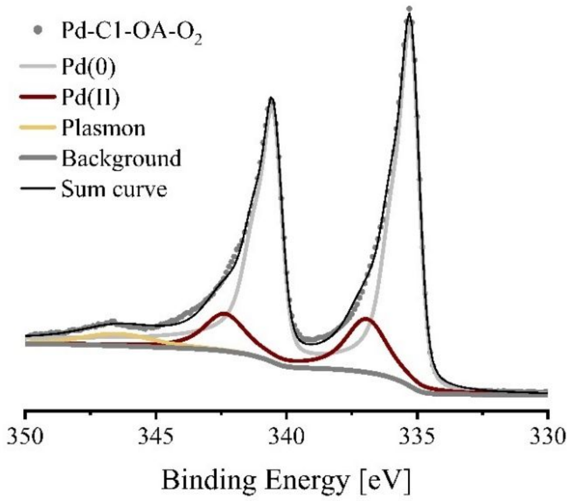

Figure S47. XPS plot of the Pd3d region of Pd-C1-OA-O_2 (fresh).

XPS analysis of the used catalyst:

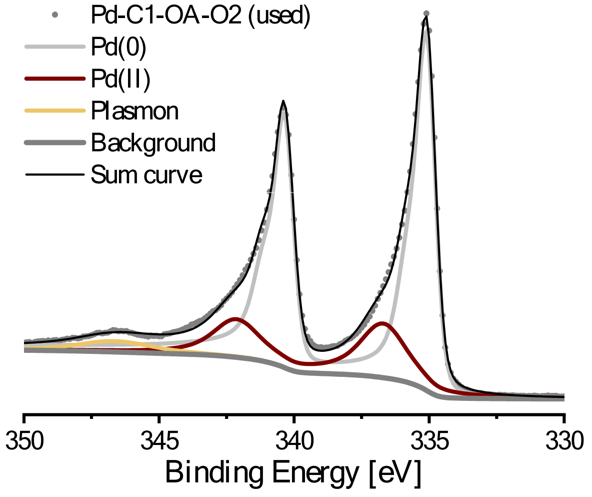

Figure S48. XPS plot of the Pd3d region of Pd-C1-OA-O_2 (used: 10h FD in fixed bed reactor).

Table S26. Ratio of Pd(0)/Pd(II) from XPS analysis for the fresh and used catalysts.

Entry	Catalyst	Pd(0) [%]	Pd(II) [%]
1	Fresh	77	23
2	After first run	74	26
3	After 9th run	83	17

Sputter Experiments

Additionally, we performed sputter experiments for selected catalysts from the optimization.

Sputter experiment of Pd-C1:

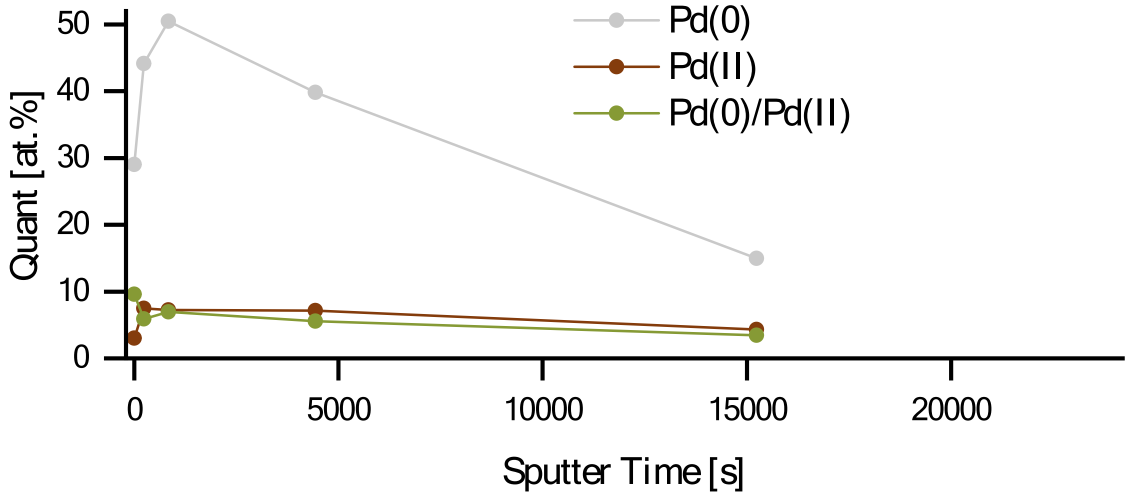

Figure S49. Results of the sputter experiment.

Sputter experiment of 1.60Pd-C1-OA:

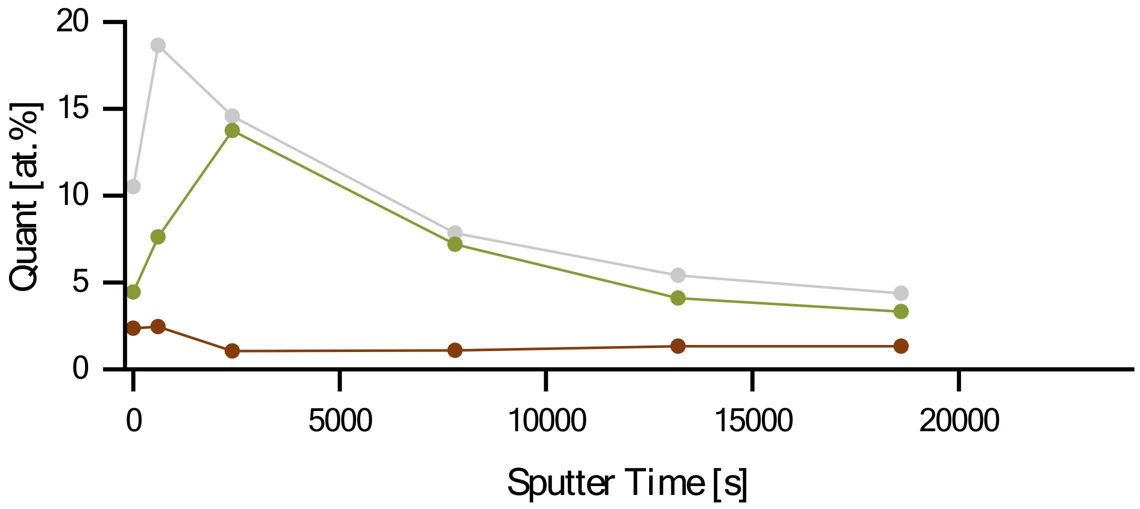

Figure S50. Results of the sputter experiment.

Sputter experiment of Pd-C1-OA-O_2:

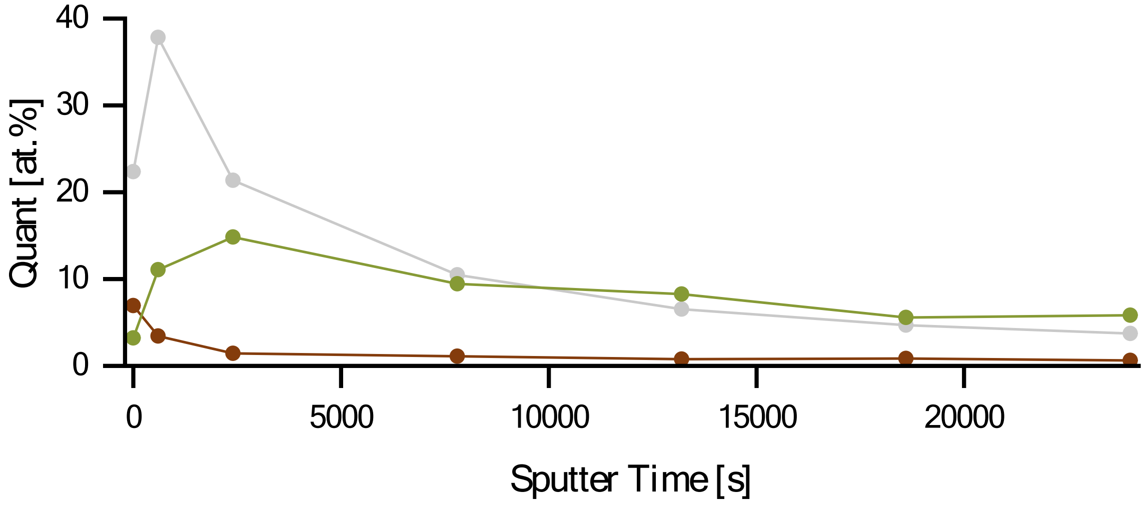

Figure S51. Results of the sputter experiment.

4.7 STEM Data of Different Catalysts

Different generations of the catalyst were analyzed *via* STEM to get a better understanding of the morphology of the palladium on the carbon support surface beyond the resolution and detection limits of SEM and XRD. For analysis of the heterogeneous pellets, the surface was scraped off the surface and the obtained powder was measured.

STEM data of Pd-C1:

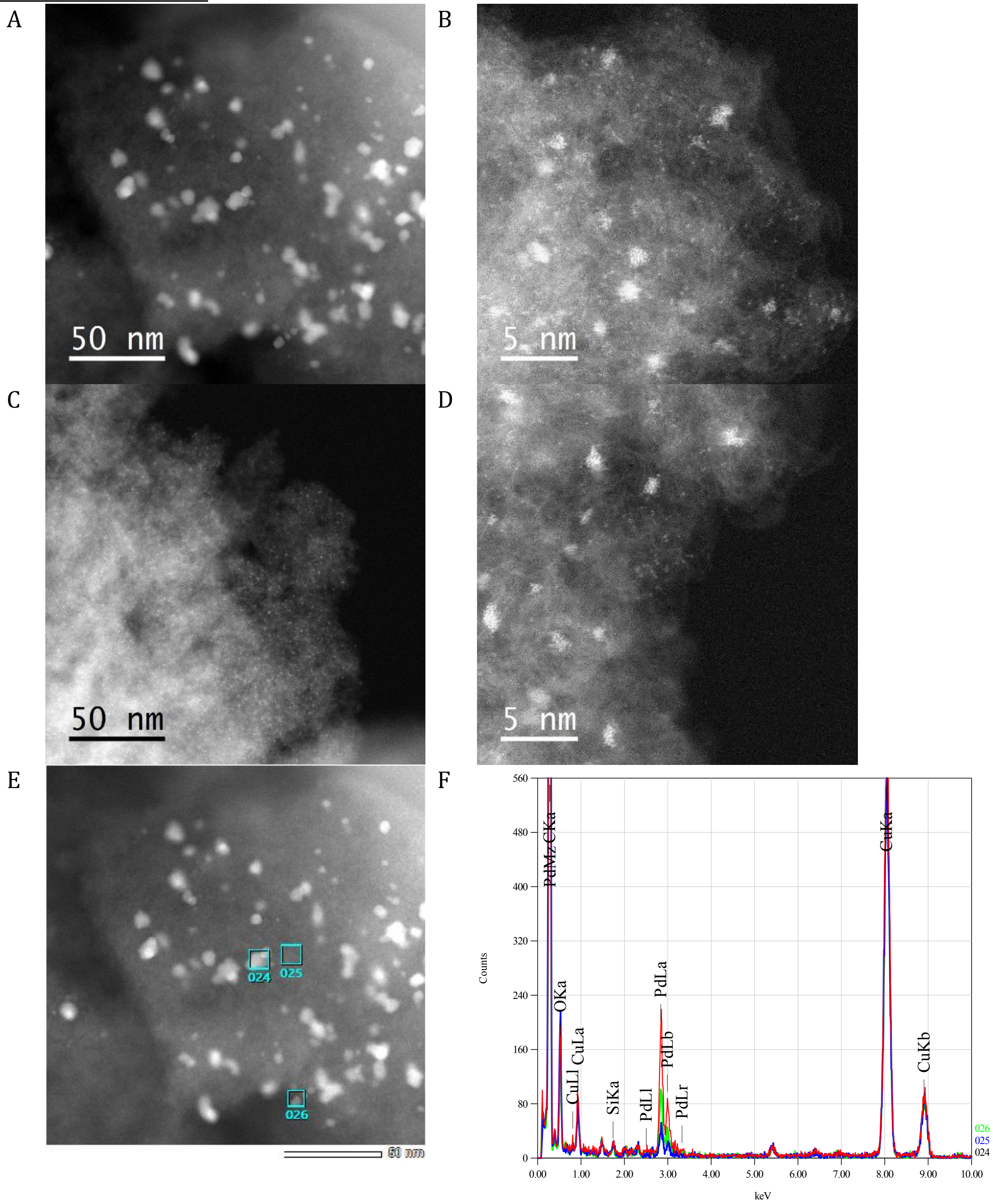

Figure S52. HAADF-STEM images of Pd-C1 (A-E) and an EDS spectrum (F) corresponding to G. Palladium loading of the sample is 3.33 wt.%.

Palladium in the magnification range investigated here seems to be predominantly distributed in the form of single atoms, clusters and small particles not forming crystallites. Very few large Pd

crystals and seldom medium sized particles are present. Oxygen is present almost everywhere on the support, potentially surface adherent water. A few crystals corresponding to. SiPO particles are present (not shown here), which agrees with the elementary composition of the support and catalysts (see ICP, XRF).

STEM data of Pd-C1-400:

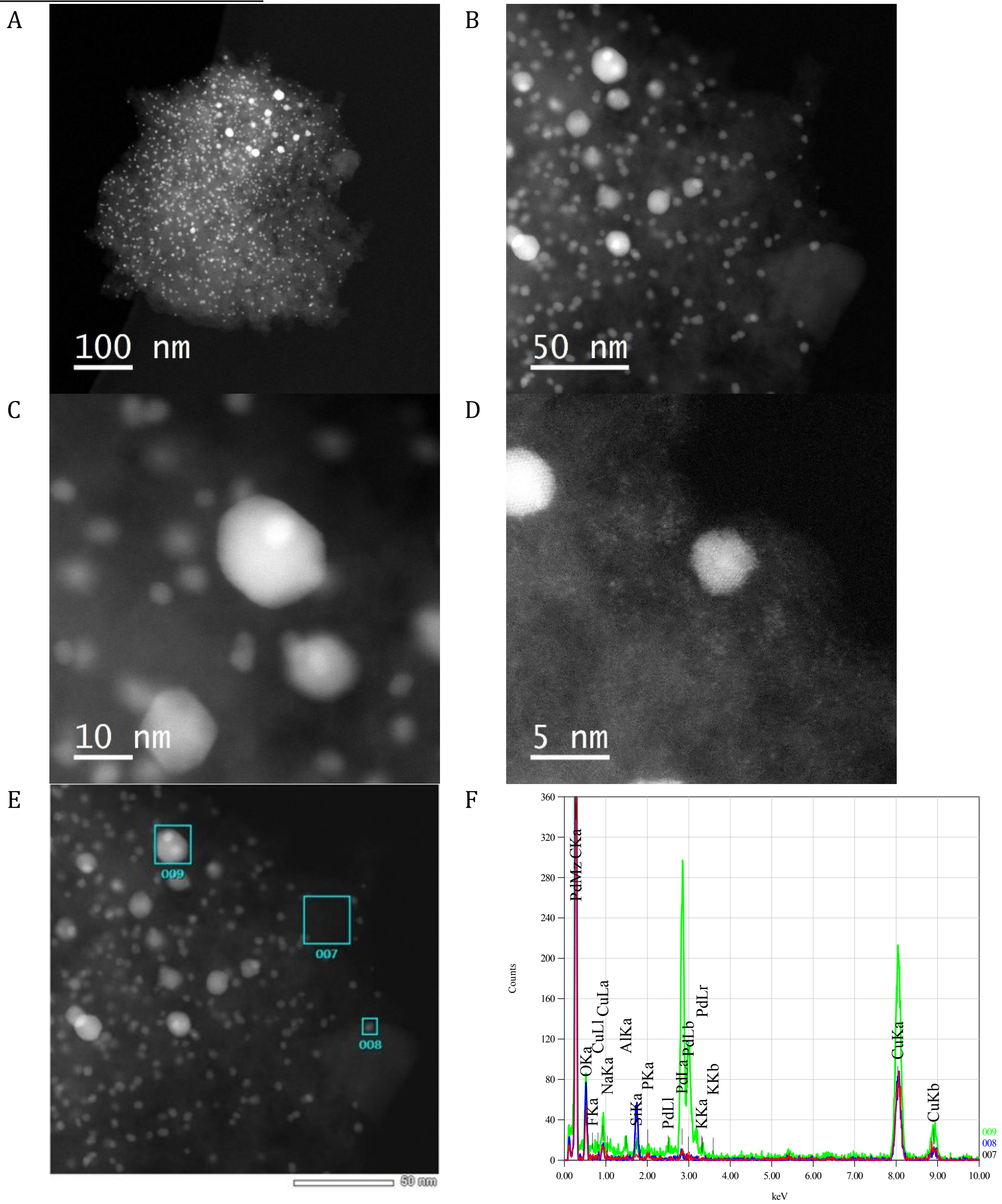

Figure S53. HAADF-STEM images of Pd-C1-400 (A-E) and an EDS spectrum (F) corresponding to E. Palladium loading of the sample is 4.23 wt.%.

The Pd particles are bigger compared to Pd-C1. Pd single atoms are also present, but the metal is mostly in form of 5-10 nm crystalline particles. This phenomenon can be attributed to the higher reduction temperature which caused particle growth. A few large Pd particles are also present. Oxygen is present almost everywhere on the support, potentially surface adherent water. Again, SiPO is present, sometime in conjunction with K, Fe, Al, Na.

STEM data of 1.60Pd-C1-OA:

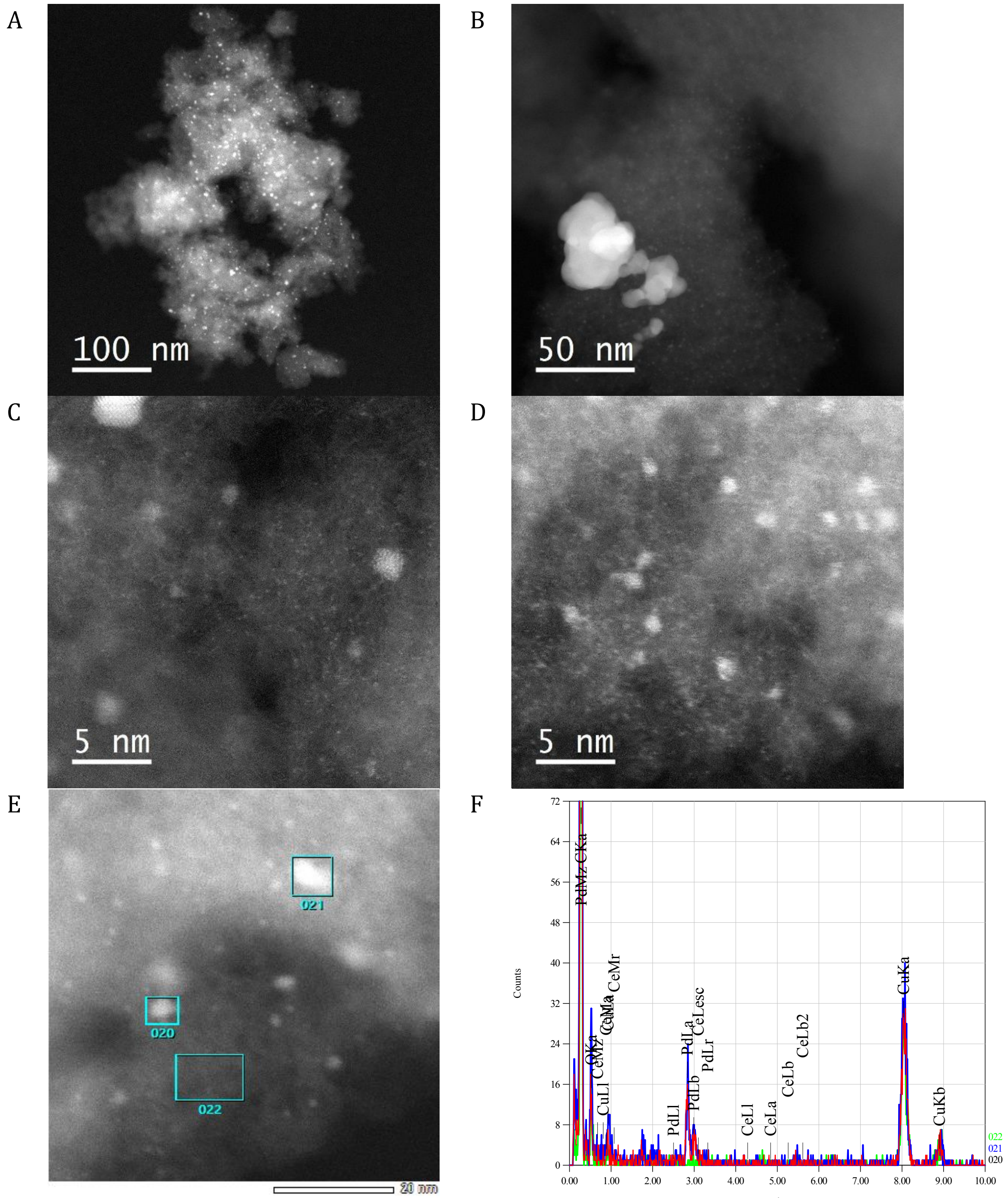

Figure S54. HAADF-STEM images of 1.60Pd-C1-OA (A-E) and an EDS spectrum (F) corresponding to E. Palladium loading of the sample is 1.60 wt.%.

Pd particle sizes found range from single atoms to tens of nm, comparable to the original catalysts Pd-C1. Apparently, the additive oxalic acid didn't influence the microscopic distribution of the palladium on the carbon support. Nevertheless, differences become obvious on a macroscopic scale. The oxalic acid ensures an even distribution of the metal even at lower loadings (see SEM). SiO_2, Al_2O_3 also found intermixed with support, which is in agreement with the elementary composition of the carbon support (see XRF). Contaminants seem to comprise Ce oxide and also small amounts of Au particles. Oxigen is present almost everywhere on the support, potentially surface adherent water.

STEM data of fresh Pd-C1-OA-O_2:

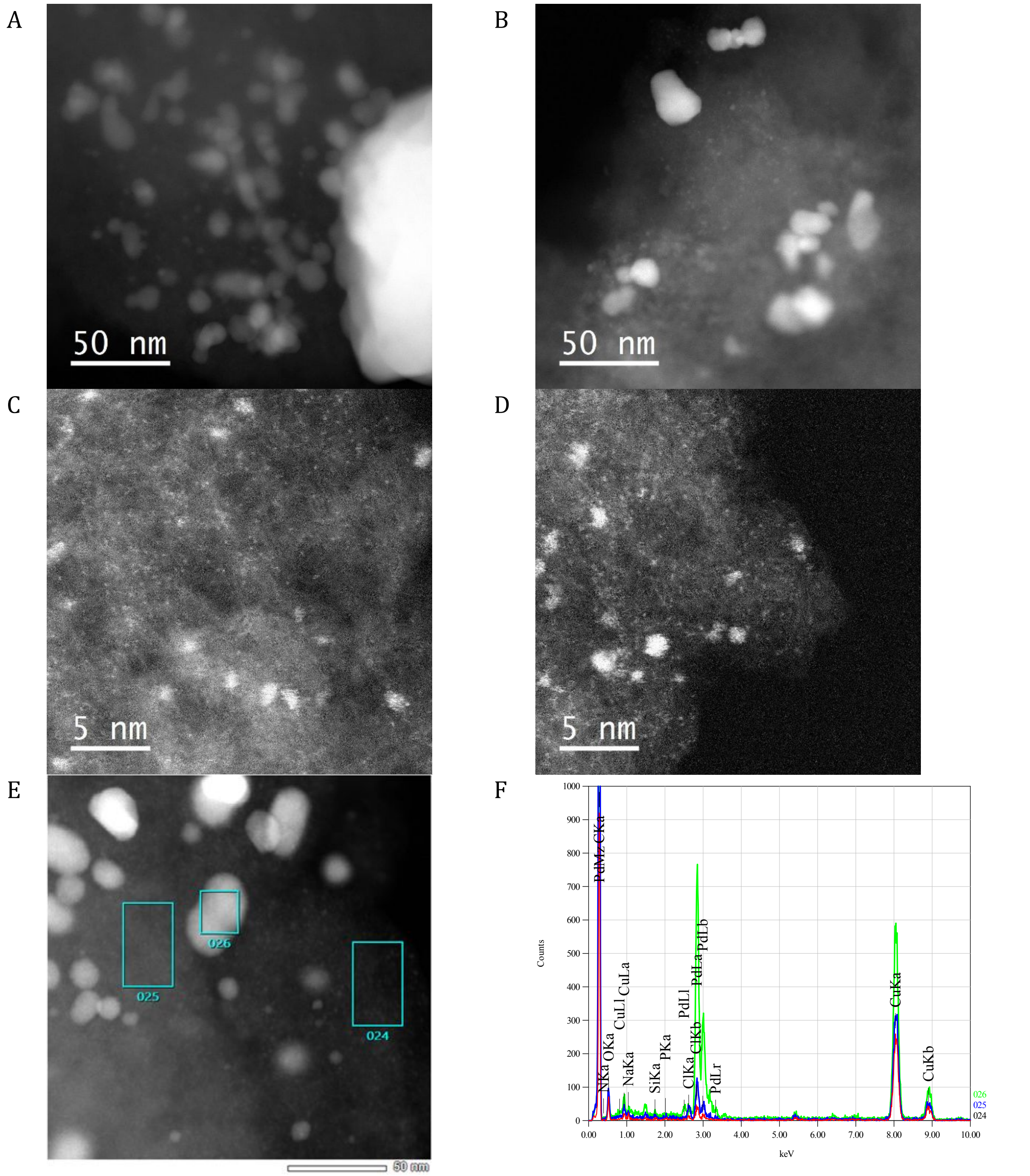

Figure S55. HAADF-STEM images of Pd-C1-OA-O_2 (A-E) and an EDS spectrum (F) corresponding to E. Palladium loading of the sample is 1.81 wt.%.

Pd particle sizes range from ca. 200 nm down to clusters and single atoms. This is similar to the original catalyst Pd-C1. There seems to be a gap between the very large fraction of palladium and one of about 20 nm. In general, the calcination step does not seem to have a drastic effect on the particle size of palladium. Oxygen is present almost everywhere on support, potentially surface adherent water Si, P, O present in form of crystallites.

STEM data of used (FD) Pd-C1-OA-O_2:

The catalyst was used for 18 h in the dehydrogenation of bicarbonate in a batch reactor. Loss of palladium can be attributed to mechanical breaking due to the stirring bar.

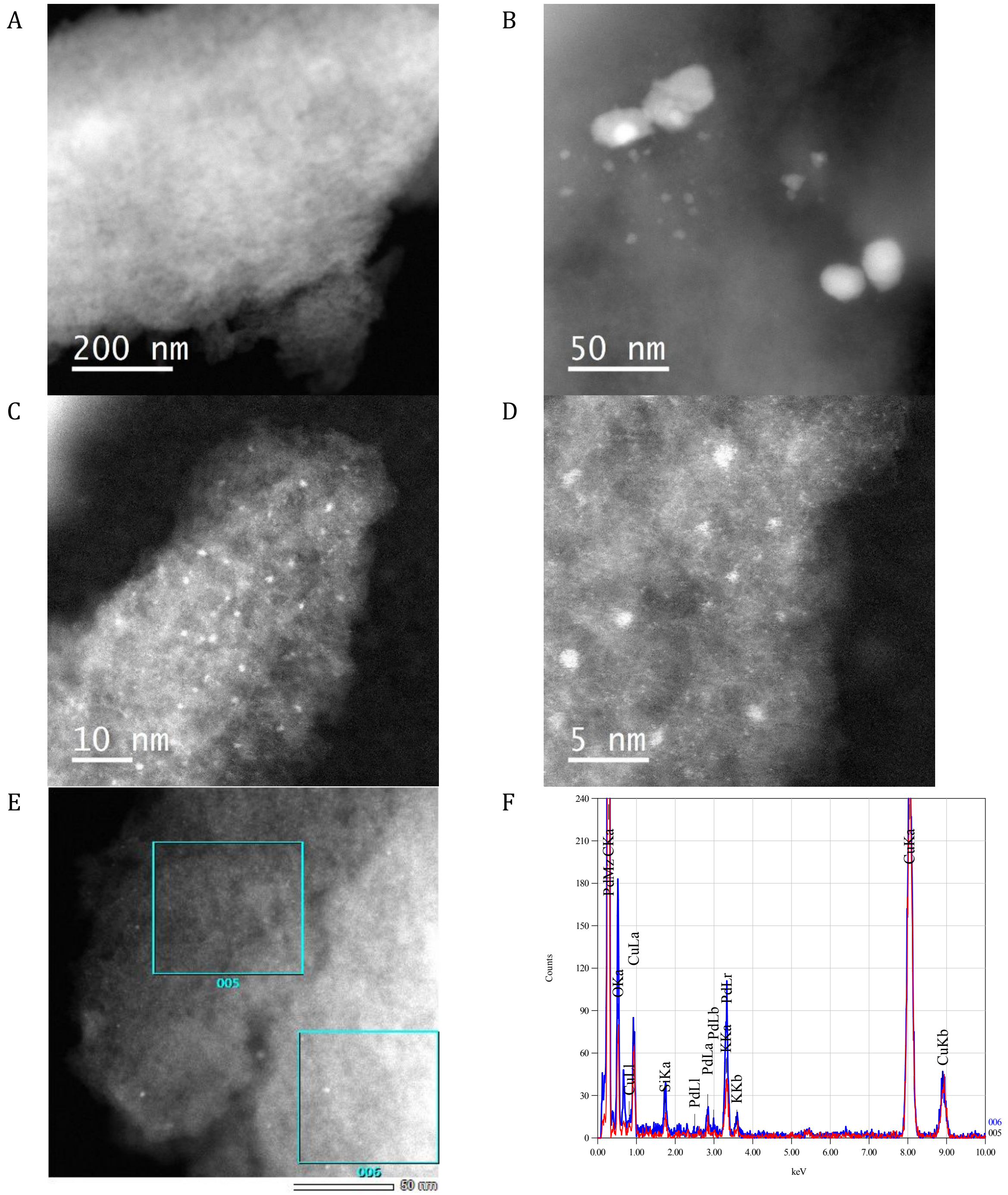

Figure S56. HAADF-STEM images of the used catalyst Pd-C1-OA-O_2 (A-E) and an EDS spectrum (F) corresponding to E. Palladium loading of the sample is 0.79 wt.%.

The palladium particle sizes ranges from ca. 50 nm (very few) down to mostly clusters and single atoms Other then the lower palladium loading, no real difference to the fresh catalyst can be detected. Oxygen is present almost everywhere on support, potentially surface adherent water. Si (ca. 0.5 at%), K (ca. 0.65 at%) was found as contaminents, seldom Al. No sulfur was detected. Poisoning of the catalyst by sulfur can therefore be ruled out.

STEM data of used (BH) Pd-C1-OA-O_2:

The catalyst was used for 18 h in the hydrogenation of bicarbonate in a batch reactor. Loss of palladium can be attributed to mechanical breaking due to the stirring bar.

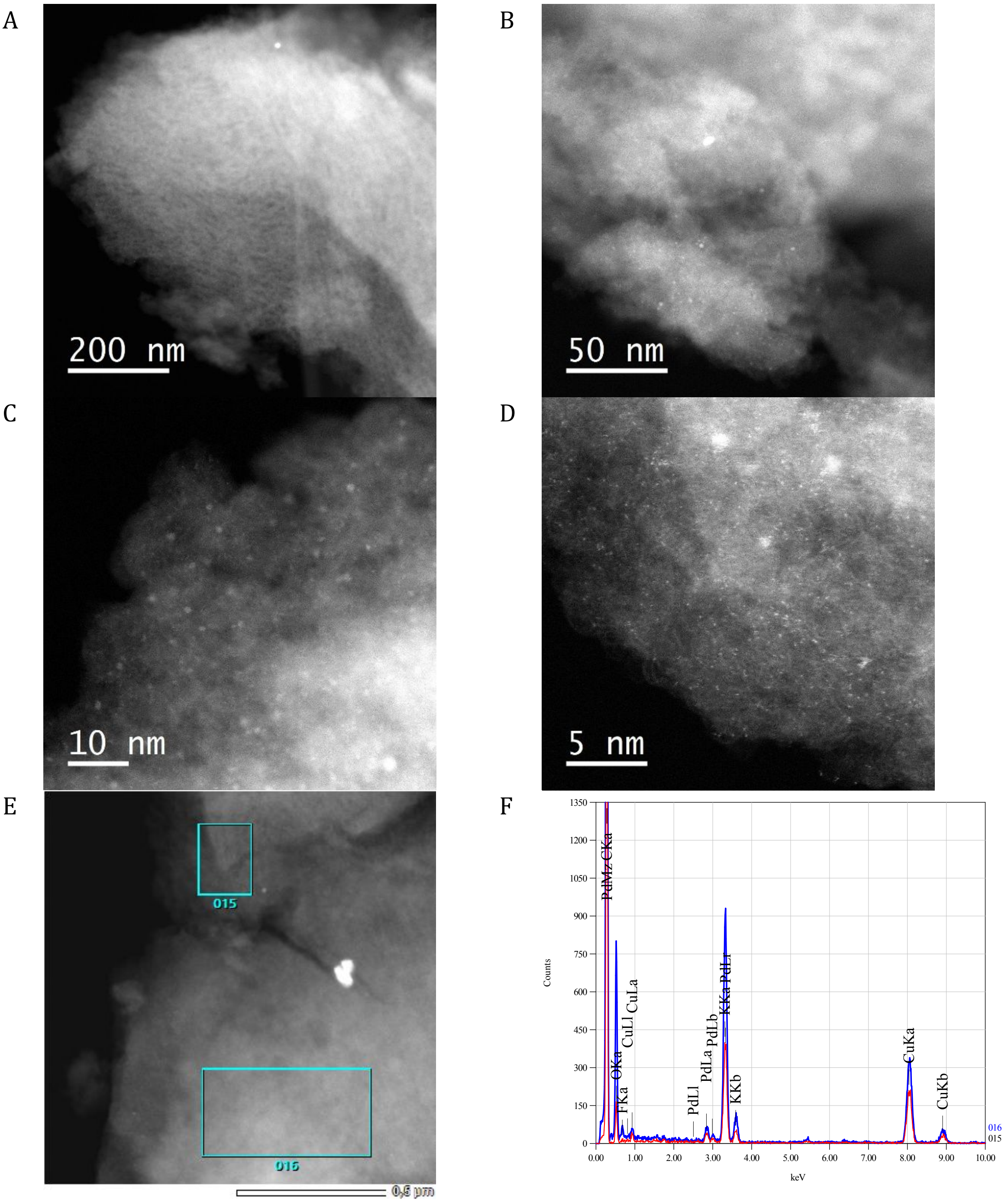

Figure S57. HAADF-STEM images of used Pd-C1-OA-O_2 (A-E) and an EDS spectrum (F) corresponding to E. Palladium loading of the sample is 0.80 wt.%.

The palladium particle sizes ranges from ca. 50 nm (very few) down to mostly clusters and single atoms Other then the lower palladium loading, no real difference to the fresh catalyst can be detected. Oxygen is present almost everywhere on support, potentially surface adherent water. Silicium and potassium (ca. 2.55 at%) was found as contaminents. The potassium content was higher then in the used catatlyst from the dehydrogenation. A high potassium content could cause an intoxication of the surface. No sulfur was detected. Poisoning of the catalyst by sulfur can therefore be ruled out.

4.8 CO Chemisorption for the Catalysts

Table S27. CO Chemisorption of four fresh and one used catalyst

Sample	Pd-C1	3.90Pd-C1-OA	1.60Pd-C1-OA	Pd-C1-OA-O_2	Pd-C1-OA-O_2 used
Surface area [m^2/g_{Sample}]	0.7999	2.2829	0.9218	1.4158	0.7356
Surface area [m^2/g_{Pd}]	24.7640	58.5370	57.6109	89.0435	55.3093
Active particle diameter [nm]	20.1570	8.5274	8.6645	5.6059	9.0250
Cubmic particle diameter [nm]	16.7975	7.1062	7.2204	4.6716	7.5209
Metal dispersion [%]	5.5595	13.1415	12.93336	19.9902	12.4169

To understand the superior activity of the final catalysts PD-C1-OA-O_2 we provide a correlation between the metal dispersion, the ration of Pd(0)/Pd(II) and the catalytic activity in Figure S66:

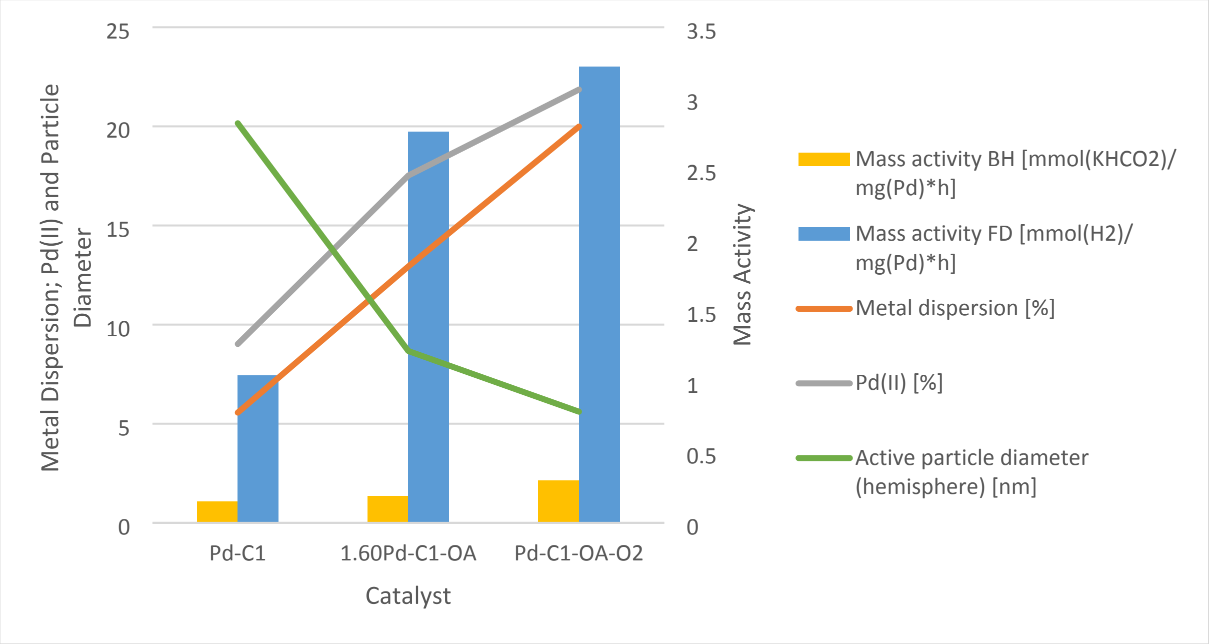

Figure S58. Correlation between the metal dispersion, the ration of Pd(0)/Pd(II) and the catalytic activity.

4.9 Zeta Potential and IEP for the Carbon Supports

Table S28. Zeta Potential and IEP for different carbon supports.

Sample	Isoelectric point	Pd loading [wt.%]
AKROS C1	1.952	3.33
COC 4x8	2.087	2.78
AFA-4-1050-4	1.976	1.74
EcoSorb CK1	2.725	3.18
WOS 4x8	1.62	3.65
EcoSorb CE55	2.173	2.70
EcoSorb CE70	1.986	4.91
EcoSorb CK4	2.843	2.79
COLPA 60	1.923	3.19
AFA-4-dot-S	3.119	0.38
Depotac 100	1.998	0.11

4.10 DRIFT Data for the Final Catalysts Pd-C1-OA-O_2

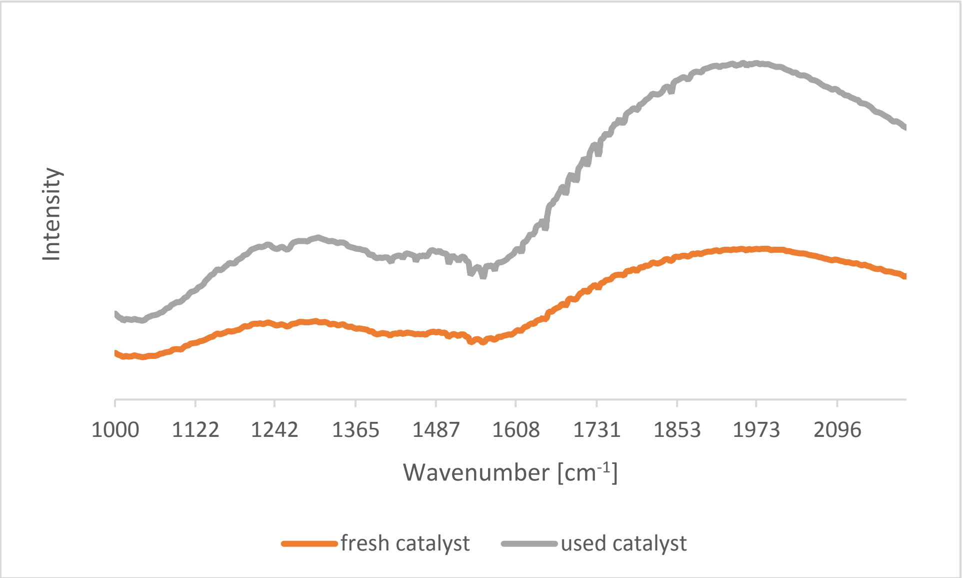

Figure S59. DRIFTS spectra of fresh and used catalysts after the 11th run.

5. Reaction Mechanisms for Pd/C Catalysts

Based on our analytical data, especially the importance of the correct ratio of Pd(0) and Pd(II) we suggest a mechanism analogue to the ones suggested for bimetallic systems, by the groups of Yamashita (PdAg; DOI: 10.1021/acsaem.0c00744) and Shishido (PdAu; DOI: 10.1021/acssuschemeng.8b04698). Instead of mixing two metals to provide electron-rich and electron lean species, our catalysts provides a good ratio of one metal in different oxidation states. In the case of our catalysts, the reaction generally occurs at the interface between Pd(0) and Pd(II), emphasizing the importance of the optimal Pd(0)/Pd(II) ratio.

FD: The dehydrogenation of the hydrogen carrier begins with the adsorption of a lone electron pair of oxygen on the electron-deficient Pd(II) sites (grey). This is followed by a nucleophilic attack by the lone pair of a water molecule on the carbon atom of the formate, accompanied by the elimination of a hydrogen atom. Next, the C-H bond of the formate is broken at Pd(0) sites, and bicarbonate is released. Finally, two hydrogen atoms combine to form a hydrogen molecule. In this mechanism, the rate-determining step is considered to be either the nucleophilic attack by water on the carbon atom of the formate or the cleavage of the C-H bond.

BH: Hydrogen dissociates heterolytically at Pd(0) sites, while a lone electron pair on the oxygen atom of bicarbonate is adsorbed on electron-poor Pd(II) sites. Then, a carbon atom of the bicarbonate undergoes a nucleophilic attack by a hydrogen atom from a nearby electron-rich Pd(0) site, forming an intermediate. OH^- is eliminated from this intermediate, and formate desorbs. Finally, H_2O forms and desorbs from the catalyst. For this mechanism, the nucleophilic attack of an H atom on the C atom of bicarbonate is considered the rate-determining step.

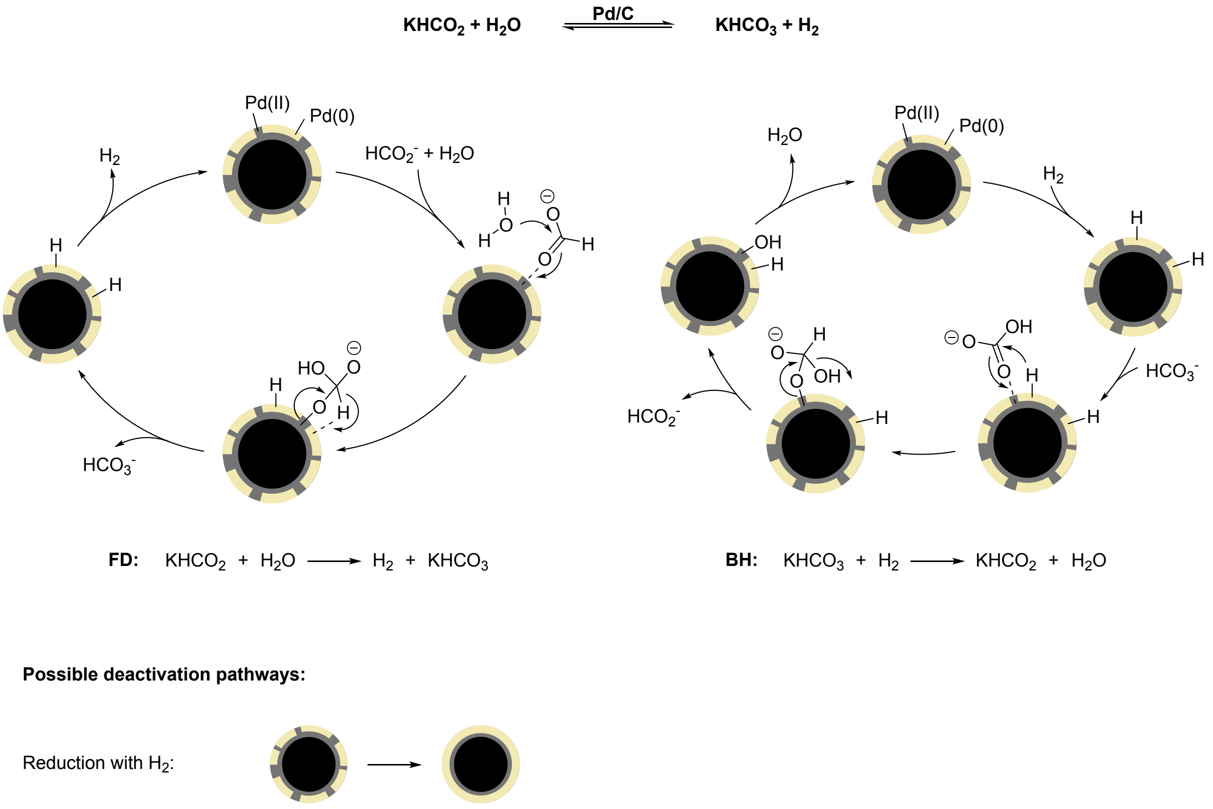

Figure S60. Proposed reaction mechanism for the reversible dehydrogenation of formate (FD; left) and the hydrogenation of bicarbonate (BH; right). Possible deactivation mechanisms discussed in this paper are presented on the bottom.

6. References

(1) Boddien, A.; Gärtner, F.; Federsel, C.; Sponholz, P.; Mellmann, D.; Jackstell, R.; Junge, H.; Beller, M. CO_2-“Neutral” Hydrogen Storage Based on Bicarbonates and Formates. *Angew. Chem. Int. Ed.* **2011**, *50* (28), 6411–6414. https://doi.org/10.1002/anie.201101995.

(2) Papp, G.; Csorba, J.; Laurenczy, G.; Joó, F. A Charge/Discharge Device for Chemical Hydrogen Storage and Generation. *Angew. Chem.* **2011**, *123* (44), 10617–10619. https://doi.org/10.1002/ange.201104951.

(3) Hull, J. F.; Himeda, Y.; Wang, W.-H.; Hashiguchi, B.; Periana, R.; Szalda, D. J.; Muckerman, J. T.; Fujita, E. Reversible Hydrogen Storage Using CO2 and a Proton-Switchable Iridium Catalyst in Aqueous Media under Mild Temperatures and Pressures. *Nat. Chem.* **2012**, *4* (5), 383–388. https://doi.org/10.1038/nchem.1295.

(4) Sasson. A METHOD FOR STORAGE AND RELEASE OF HYDROGEN. EP 3 065 865 B1, 2022.

(5) Bi, Q.; Lin, J.; Liu, Y.; Du, X.; Wang, J.; He, H.; Cao, Y. An Aqueous Rechargeable Formate-Based Hydrogen Battery Driven by Heterogeneous Pd Catalysis. *Angew. Chem. Int. Ed.* **2014**, *53* (49), 13583–13587. https://doi.org/10.1002/anie.201409500.

(6) Enthaler, S.; Brück, A.; Kammer, A.; Junge, H.; Irran, E.; Gülak, S. Exploring the Reactivity of Nickel Pincer Complexes in the Decomposition of Formic Acid to CO_2/H_2 and the Hydrogenation of $NaHCO_3$ to HCOONa. *ChemCatChem* **2015**, *7* (1), 65–69. https://doi.org/10.1002/cctc.201402716.

(7) Horváth, H.; Papp, G.; Kovács, H.; Kathó, Á.; Joó, F. Iridium(I) NHC-Phosphine Complex-Catalyzed Hydrogen Generation and Storage in Aqueous Formate/Bicarbonate Solutions Using a Flow Reactor - Effective Response to Changes in Hydrogen Demand. *Int. J. Hydrog. Energy* **2019**, *44* (53), 28527–28532. https://doi.org/10.1016/j.ijhydene.2018.12.119.

(8) Kothandaraman, J.; Czaun, M.; Goeppert, A.; Haiges, R.; Jones, J.; May, R. B.; Prakash, G. K. S.; Olah, G. A. Amine-Free Reversible Hydrogen Storage in Formate Salts Catalyzed by Ruthenium Pincer Complex without pH Control or Solvent Change. *ChemSusChem* **2015**, *8* (8), 1442–1451. https://doi.org/10.1002/cssc.201403458.

(9) Sordakis, K.; Dalebrook, A. F.; Laurenczy, G. A Viable Hydrogen Storage and Release System Based on Cesium Formate and Bicarbonate Salts: Mechanistic Insights into the Hydrogen Release Step. *ChemCatChem* **2015**, *7* (15), 2332–2339. https://doi.org/10.1002/cctc.201500359.

(10) Koh, K.; Jeon, M.; Chevrier, D. M.; Zhang, P.; Yoon, C. W.; Asefa, T. Novel Nanoporous N-Doped Carbon-Supported Ultrasmall Pd Nanoparticles: Efficient Catalysts for Hydrogen Storage and Release. *Applied Catalysis B: Environmental* **2017**, *203*, 820–828. https://doi.org/10.1016/j.apcatb.2016.10.080.

(11) Zhong, H.; Iguchi, M.; Chatterjee, M.; Ishizaka, T.; Kitta, M.; Xu, Q.; Kawanami, H. Interconversion between CO_2 and HCOOH under Basic Conditions Catalyzed by PdAu Nanoparticles Supported by Amine-Functionalized Reduced Graphene Oxide as a Dual Catalyst. *ACS Catal.* **2018**, *8* (6), 5355–5362. https://doi.org/10.1021/acscatal.8b00294.

(12) Xin, Z.; Zhang, J.; Sordakis, K.; Beller, M.; Du, C.; Laurenczy, G.; Li, Y. Towards Hydrogen Storage through an Efficient Ruthenium-Catalyzed Dehydrogenation of Formic Acid. *ChemSusChem* **2018**, *11* (13), 2077–2082. https://doi.org/10.1002/cssc.201800408.

(13) Wei, D.; Shi, X.; Sponholz, P.; Junge, H.; Beller, M. Manganese Promoted (Bi)Carbonate Hydrogenation and Formate Dehydrogenation: Toward a Circular Carbon and Hydrogen Economy. *ACS Cent. Sci.* **2022**, *8* (10), 1457–1463. https://doi.org/10.1021/acscentsci.2c00723.

(14) Sang, R.; Stein, C. A. M.; Schareina, T.; Hu, Y.; Léval, A.; Massa, J.; Turan, V.; Sponholz, P.; Wei, D.; Jackstell, R.; Junge, H.; Beller, M. Development of a Practical Formate/Bicarbonate Energy System. *Nat. Commun.* **2024**, *15* (1), 7268. https://doi.org/10.1038/s41467-024-51658-2.

(15) Wang, Z.-L.; Yan, J.-M.; Wang, H.-L.; Ping, Y.; Jiang, Q. Pd/C Synthesized with Citric Acid: An Efficient Catalyst for Hydrogen Generation from Formic Acid/Sodium Formate. *Sci. Rep.* **2012**, *2* (1), 598. https://doi.org/10.1038/srep00598.
